# Discovery of new nicotinamides as apoptotic VEGFR-2 inhibitors: virtual screening, synthesis, anti-proliferative, immunomodulatory, ADMET, toxicity, and molecular dynamic simulation studies

**DOI:** 10.1080/14756366.2022.2070744

**Published:** 2022-05-16

**Authors:** Reda G. Yousef, Albaraa Ibrahim, Mohamed M. Khalifa, Wagdy M. Eldehna, Ibraheem M. M. Gobaara, Ahmed B. M. Mehany, Eslam B. Elkaeed, Aisha A. Alsfouk, Ahmed M. Metwaly, Ibrahim H. Eissa

**Affiliations:** a Pharmaceutical Medicinal Chemistry & Drug Design Department, Faculty of Pharmacy (Boys), Al-Azhar University, Cairo, Egypt; b School of Biotechnology, Badr University in Cairo, Badr City, Egypt; c Department of Pharmaceutical Chemistry, Faculty of Pharmacy, Kafrelsheikh University, Kafrelsheikh, Egypt; d Zoology Department, Faculty of Science (Boys), Al-Azhar University, Cairo, Egypt; e Department of Pharmaceutical Sciences, College of Pharmacy, AlMaarefa University, Riyadh, Saudi Arabia; f Department of Pharmaceutical Sciences, College of Pharmacy, Princess Nourah Bint Abdulrahman University, Riyadh, Saudi Arabia; g Pharmacognosy and Medicinal Plants Department, Faculty of Pharmacy (Boys), Al-Azhar University, Cairo, Egypt; h Biopharmaceutical Products Research Department, Genetic Engineering and Biotechnology Research Institute, City of Scientific Research and Technological Applications (SRTA-City), Alexandria, Egypt

**Keywords:** Apoptosis, anticancer, immunomodulatory, VEGFR-2 inhibitors, molecular dynamic simulation

## Abstract

A library of modified VEGFR-2 inhibitors was designed as VEGFR-2 inhibitors. Virtual screening was conducted for the hypothetical library using *in silico* docking, ADMET, and toxicity studies. Four compounds exhibited high *in silico* affinity against VEGFR-2 and an acceptable range of the drug-likeness. These compounds were synthesised and subjected to *in vitro* cytotoxicity assay against two cancer cell lines besides VEGFR-2 inhibitory determination. Compound **D-1** showed cytotoxic activity against HCT-116 cells almost double that of sorafenib. Compounds **A-1**, **C-6**, and **D-1** showed good IC_50_ values against VEGFR-2. Compound **D-1** markedly increased the levels of caspase-8 and BAX expression and decreased the anti-apoptotic Bcl-2 level. Additionally, compound **D-1** caused cell cycle arrest at pre-G1 and G2-M phases in HCT-116 cells and induced apoptosis at both early and late apoptotic stages. Compound **D-1** decreased the level of TNF-α and IL6 and inhibited TNF-α and IL6. MD simulations studies were performed over 100 ns.

## 1. Introduction

During the journey of the search and discovery of novel potent anticancer agents, the anti-angiogenic class of drugs has gained much attention in the last few decades^1^. These agents were proved to hinder the uncontrolled development of new capillaries from the pre-existing blood vessels, a process known as angiogenesis^2–4^. However, understanding, as well as management of the angiogenic mechanism, is still a promising approach to tackling cancer development.

The role of growth factors in angiogenesis control has been emphasised. Among them, vascular endothelial growth factors (VEGFs) were evidenced to be the key players to regulate angiogenesis^5^. VEGFs exert their action upon binding to three different tyrosine kinase (TK) receptors, namely, VEGFR-1, VEGFR-2, and VEGFR-3^6^. Activation of these receptors regulates the angiogenic process *via* the development of the essential blood vessel networks needed to supply nutrition and oxygen for tumour growth^7^. VEGFR-2 tyrosine kinase plays a superior role over the other subtypes in promoting tumour angiogenesis. Focussing on its effect, VEGFR-2 initiates downstream signal transduction pathway *via* dimerisation followed by autophosphorylation of tyrosine receptor^8^. This pathway results finally in angiogenesis^9^. Thus, obstruction of the VEGF/VEGFR-2 signalling path or even decreasing its response is one of the topmost targets in anti-angiogenic drug discovery and cancer treatment^10^^,^^11^. Meanwhile, a considerable number of small molecules with diverse chemical structures have been clinically approved to antagonise this angiogenic pathway and, thus, serve as anticancer agents^12^. Unfortunately, the development of resistance to TK inhibitors besides their side effects were the main drawbacks of the currently known drugs. With the influence of these findings, the discovery of more effective and safer anticancer agents become a more approachable concept.

**Figure 1.**
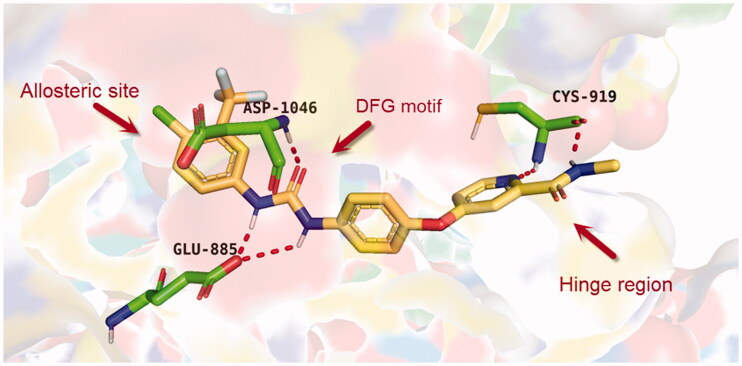
The three active site regions of the VEGFR-2 receptor with the native inhibitor, sorafenib.

Over the last few years, our research co-workers have developed a promising project concerning the discovery of new TK inhibitors, particularly with VEGFR-2 inhibitory activity^13–20^. In this regard, we have introduced several small molecules that were efficiently proved to possess strong VEGFR-2 inhibitory activities that were, in some cases, higher than that of the reference drugs. One of the most promising skeletons in our research project was the pyridine scaffold^21^. Pyridine was the backbone of several well-known VEGFR-2 inhibitors^22–24^. Taking sorafenib **1**, the pyridine-based FDA-approved VEGFR-2 inhibitor, as a lead compound, different studies were developed to discover new inhibitors with higher potency and lower side effects, as well. Investigation of the binding of sorafenib to the VEGFR-2 active site gave us a brief about the main three pockets in which sorafenib interacts to perform its action^25^. However, the first pocket of the VEGFR-2 active site is an ATP pocket to which the pyridine moiety of sorafenib binds^25^. The second pocket is the DFG motif of the enzyme that interacts with the urea part of sorafenib *via* different H-bonding interactions^26^. While the last pocket is an allosteric lipophilic site where the terminal substituted phenyl ring of sorafenib occupies^27^ (Figure 1).

### 1.1. Rationale and structure-based design

In continuation of our recent work regarding the discovery of potent pyridine-based VEGFR-2 inhibitors, our research team has performed a targeted computational screening study for different suggested pyridine-containing scaffolds in the hope of getting more potent congeners. However, four pyridine scaffolds were included in the current study. A set of derivatives was then proposed corresponding to each scaffold. The proposed derivatives were evaluated computationally using the molecular docking tool to get insights into their binding with the VEGFR-2 active site. The best member of each scaffold was detected depending on its free binding energy and the similarity of its binding pattern to that of the reference compound, sorafenib. The best members were then synthesised and biologically assessed for their VEGFR-2 inhibition as well as their cytotoxic effects.

The suggested scaffolds were precisely chosen to possess the four main pharmacophoric features of the VEGFR-2 reported inhibitors namely, a “hinge-binding” heteroaromatic head to bind to the receptor ATP pocket, a “spacer” that links the hinge-binding segment with the hydrogen-bonding moiety, a “hydrogen-bonding moiety” to occupy the DFG motif of the enzyme, and a hydrophobic “tail” directed towards the enzyme allosteric site. Guiding by our previous study, the “hinge-binding” head of the suggested structures was conserved to be a pyridine ring. Similarly, the “spacer” moiety was decided to remain a phenyl carbamoyl group. Conversely, four different hydrogen-bonding moieties, as well as four different hydrophobic tails, were incorporated into the suggested structures.

## 2. Results and discussion

### 2.1. Virtual screening

#### 2.1.1. Docking studies

A set of compounds corresponding to each suggested scaffold was computationally screened using molecular docking tools aiming to observe the way by which they interact with the VEGFR-2 TK active pocket. As so, VEGFR-2 TK crystal structure PDB ID: 4ASD with its native inhibitor, sorafenib, was adopted for this current study. However, six structures were evaluated regarding scaffold **A**, in addition to ten members for scaffold **B**, nine for scaffold **C**, and four compounds for scaffold **D**.

**Figure 2.**
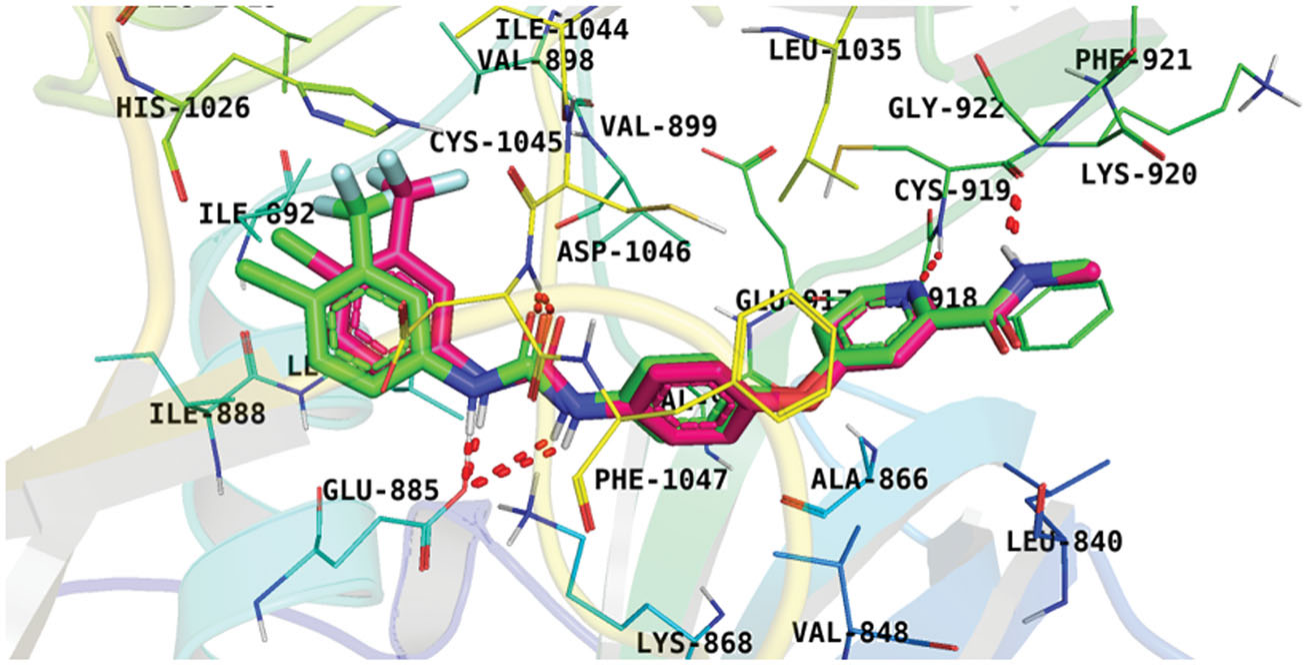
Superimposition of the native ligand (green) and the obtained pose (red) of the same ligand onto the VEGFR-2 TK active pocket.

The downloaded protein was then prepared for docking. Following, the protocol used has been validated through a redocking process of the native legend onto the active pocket. The applicability of the used protocol was, thus, established because of its capability to reproduce a binding pattern identical to that of the native ligand in the active pocket including Cys919 in the hinge region, Glu885 in the α-C helix, and Asp1046 in the DFG motif. Based on the later finding as well as the low result in RMSD (0.56 Å), the effectiveness of the suggested docking protocol was confirmed. (Figure 2).

**Figure 3.**
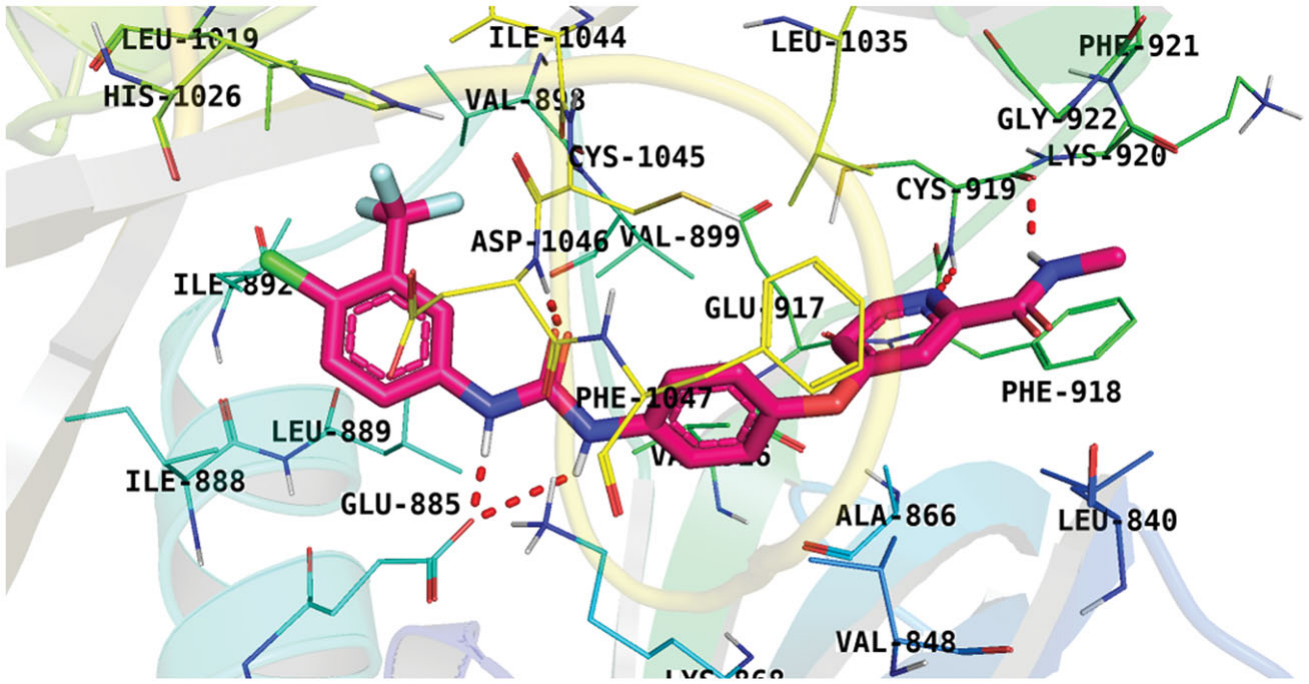
docking pose of sorafenib inside VEGFR-2 TK pocket.

Tables 1–4 demonstrated the suggested scaffolds **A**, **B**, **C**, and **D** and the proposed derivatives. The free energy of binding, as well as the essential amino acid residues that participated in the binding process, were also illustrated. Upon investigation of the proposed binding mode of sorafenib (affinity value of −20.77 kcal/mol) we can observe that it interacted with the VEGFR-2 active site *via* the formation of five H-bonding interactions. The Sorafenib’s urea moiety was directed towards the receptor DFG motif and was stabilised by three H-bonds, two with Glu885 and one with Asp1046. Regarding the hinge region, sorafenib has bound through its substituted pyridine moiety with Cys919 by two H-bonds. In addition, two hydrophobic interactions with Phe918 and Phe1047 potentiated sorafenib stabilisation in the active site (Figure 3).

**Table 1.** The suggested scaffold **A** and the screened derivatives.

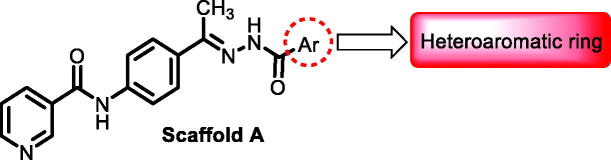			
Comp. ID	Ar	ΔG	Residues (H-bond)
**A-1**	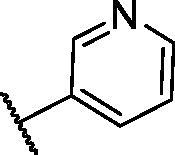	−19.82	ASP1046, Glu885, Cys919
**A-2**	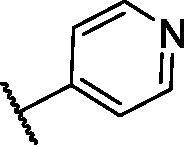	−16.29	ASP1046, Glu885
**A-3**	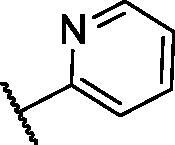	−14.42	ASP1046, Glu885
**A-4**	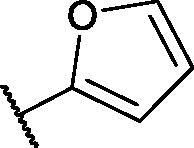	−17.19	ASP1046, Glu885
**A-5**	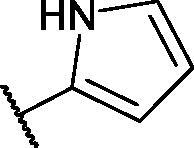	−12.74	ASP1046, Glu885
**A-6**	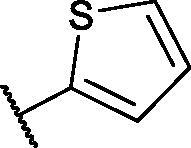	−10.73	ASP1046, Glu885

**Table 2.** The suggested scaffold **B** and the screened derivatives.

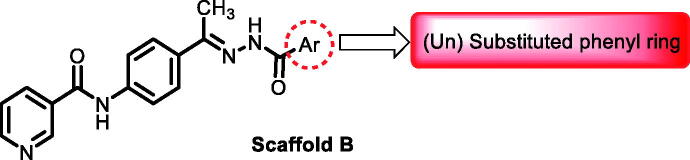			
Comp. ID	Ar	ΔG	Residues (H-bond)
**B-1**	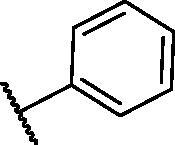	−21.62	ASP1046, Glu885, Cys919
**B-2**	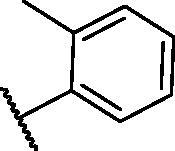	−19.87	ASP1046, Glu885, Cys919
**B-3**	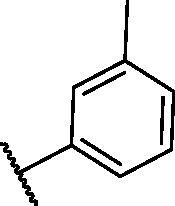	−19.73	ASP1046, Glu885, Cys919
**B-4**	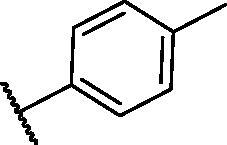	−15.22	ASP1046, Glu885
**B-5**	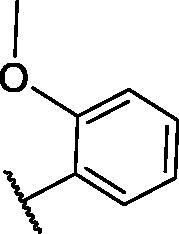	−15.71	Glu885
**B-6**	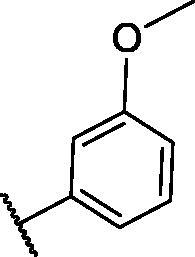	−14.59	ASP1046, Glu885
**B-7**	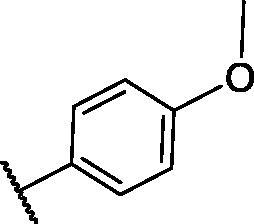	−17.73	ASP1046, Glu885, Cys919
**B-8**	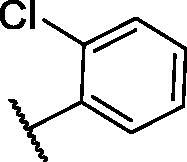	−10.43	ASP1046, Glu885, Cys919
**B-9**	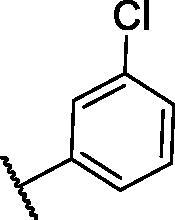	−9.65	ASP1046, Glu885, Cys919
**B-10**	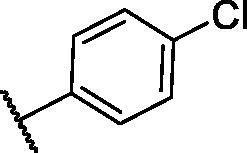	−16.33	ASP1046, Glu885, Cys919

**Table 3.** The suggested scaffold **C** and the screened derivatives.

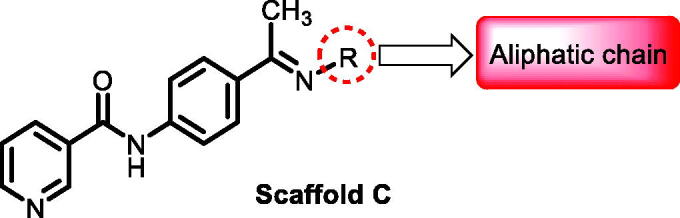			
Comp. ID	*R*	ΔG	Residues (H-bond)
**C-1**	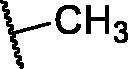	−8.61	ASP1046, Cys919
**C-2**	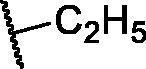	−7.39	ASP1046, Cys919
**C-3**	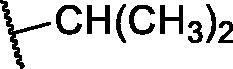	−9.44	ASP1046, Cys919
**C-4**	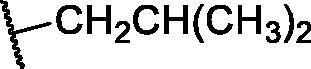	−7.22	–
**C-5**	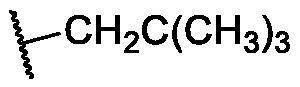	−11.21	–
**C-6**	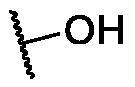	−19.09	ASP1046, Glu885, Cys919, Lys868
**C-7**	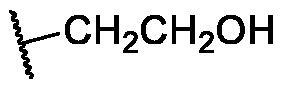	−15.21	ASP1046, Cys919
**C-8**	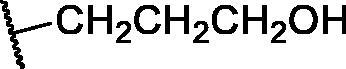	−11.33	ASP1046, Cys919
**C-9**	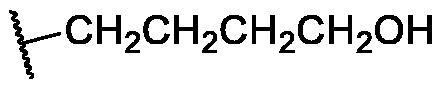	−12.79	ASP1046, Cys919

**Table 4.** The suggested scaffold **D** and the screened derivatives.

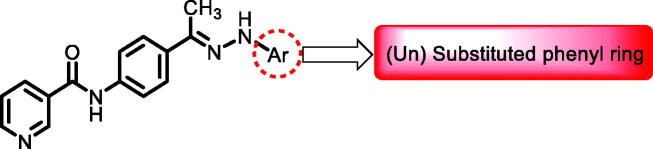			
Comp. ID	Ar	ΔG	Residues (H-bond)
**D-1**	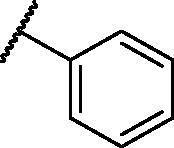	−18.21	ASP1046, Glu885, Cys919
**D-2**	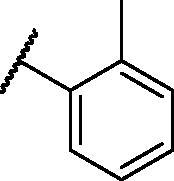	−17.29	ASP1046, Glu885, Cys919
**D-3**	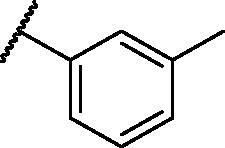	−17.01	ASP1046, Glu885, Cys919
**D-4**	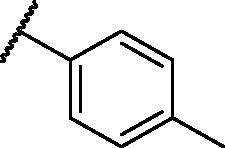	−16.41	ASP1046, Glu885, Cys919

Examination of the suggested structures revealed that most of them occupied the same orientation achieved by sorafenib. However, the noticed variation of the binding patterns between the structures and the active site besides the different free binding energies allowed us to detect the most preferred poses. The best structure of each series was then synthesised and biologically evaluated.

**Figure 4.**
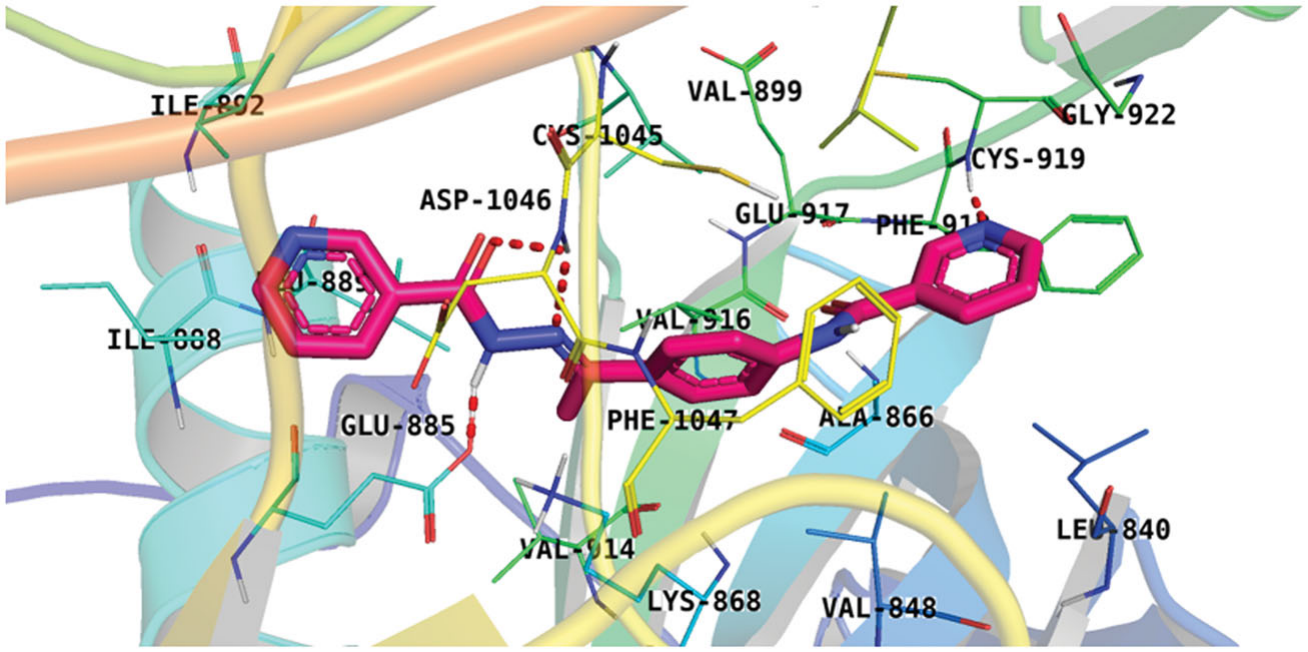
Docking pose of **A-1** inside VEGFR-2 TK pocket.

With respect to scaffold A, compound **A-1** displayed the highest binding energy Table 1. Moreover, it bound to the VEGFR-2 active pocket in a manner that was identical to that of sorafenib. A detailed investigation showed that one of its pyridine moieties occupied the hinge region with the formation of an H-bond with Cys919. Also, the hydrazineyl moiety interacted with the DFG motif of the enzyme by three H-bonds with Glu885 and Asp1045. Additionally, two pi interactions were formed between the compound and Phe918 and Phe1047 active site residues (Figure 4).

**Figure 5.**
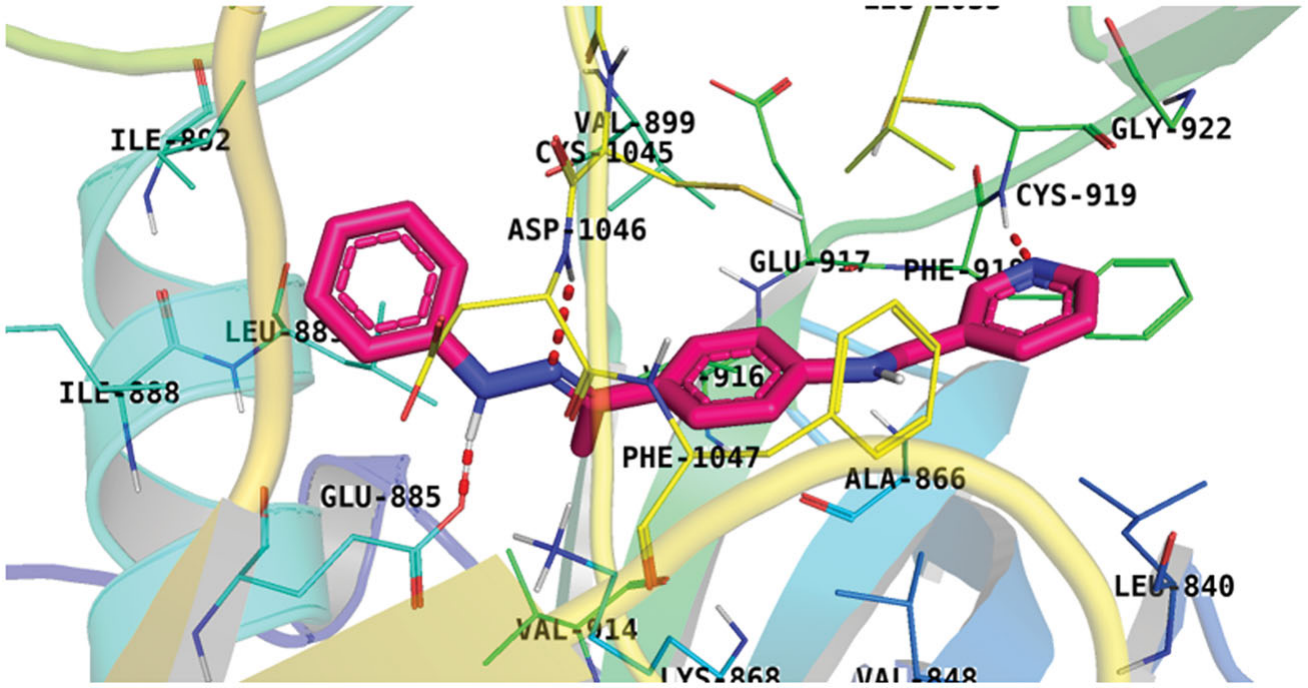
Docking pose of **B-1** inside VEGFR-2 TK pocket.

For scaffold **B**, the highest binding energy was observed for congener **B-1**. Although members **B-2,3** and **B-7** to **B-10** possessed the same binding pattern of sorafenib, the elevated binding energy of **B-1** gave it the advantage over other members (Table 2 and Figure 5) explaining the similarity of the binding mode of **B-1** to that of sorafenib.

**Figure 6.**
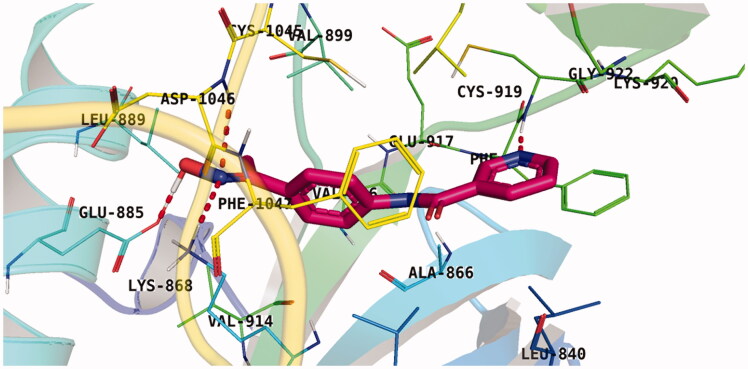
Docking pose of **C-6** inside VEGFR-2 TK pocket.

Regarding scaffold **C**, in which the hydrophobic tails are all aliphatic chains, compound **C-6** was distinguished among other members in that it had the same binding pattern as the reference drug in addition to an extra H-bond interaction with Lys868 residue Figure 6. However, its high affinity to the active site was also confirmed by its elevated free binding energy among the rest of the derivatives (Table 3).

**Figure 7.**
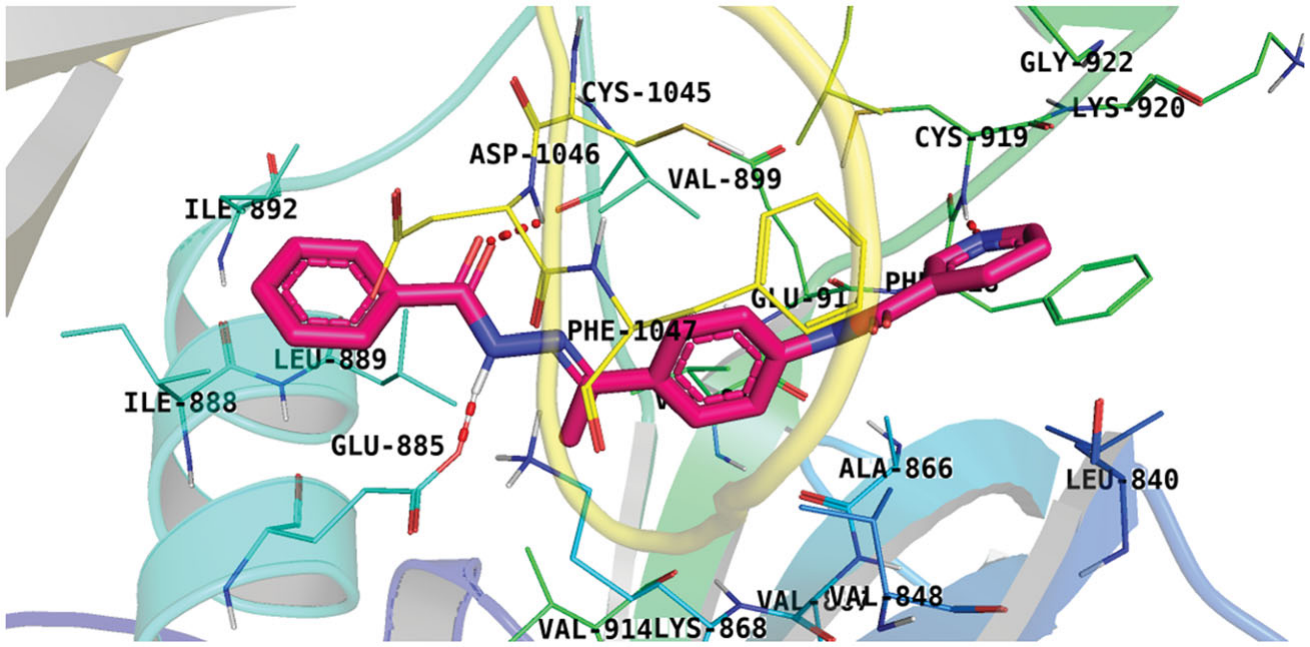
Docking pose of **D-1** inside VEGFR-2 TK pocket.

Lastly in this regard, derivatives of scaffold **D** all displayed the same orientation and binding pattern inside the active pocket. They are all bound to the receptor by three H-bonds with Asp1046, Glu885, and Cys919 residues. They only differ in the values of the free binding energy. Accordingly, compound **D-1**, with the highest energy, was identified to be the most potent one (Table 4 and Figure 7).

#### 2.1.2. *In silico* ADME study

**Figure 8.**
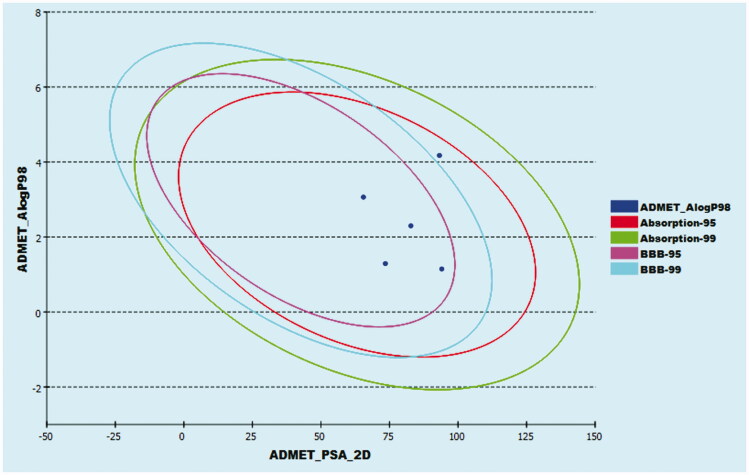
The ADME plot of the synthesised compounds.

The pharmacokinetic properties of the four selected structures were then investigated using Discovery Studio 4.0 ADME protocol. Sorafenib was co investigated as well. The four members were predicted to have good absorption percentages with low to medium BBB penetrating ability. On the other hand, all congeners exhibited good solubility levels with a cytochrome P2D6 non-inhibitory effect (Figure 8 and Table 5).

**Table 5.** ADME results of the designed congeners.

Comp.	BBB level^a^	Solubility level^b^	Absorption level^c^	CYP2D6 prediction^d^	PPB prediction^e^
**A-1**	3	3	0	×	√
**B-1**	3	3	0	×	√
**C-6**	3	3	0	×	√
**D-1**	2	3	0	×	√
Sorafenib	4	1	0	×	√

^a^BBB penetrating levels in which 0 = very high, 1 = high, 2 = medium, 3 = low, and 4 = very low.

^b^Solubility level in which 1 = very low, 2 = low, 3 = good, and 4 = optimal.

^c^Absorption level in which 0 = good, 1 = moderate, 2 = poor, and 3 = very poor.

^d^CYP2D6 is the cytochrome P2D6. The compound might be CYP2D6 inhibitor (√) or non-inhibitor (×).

^e^PPB is the plasma protein binding that might be below than 90% (×) or above than 90% (√).

#### 2.1.3. Toxicity studies

The four selected congeners as well as sorafenib were investigated for their toxicity profile using a model constructed in Discovery studio software version 4.0^28^. Minimal toxicity prediction was observed for the four members. Moreover, the four members were neither carcinogenic nor mutagenic. The carcinogenic potency TD_50_ values ranged from 19.559 to 108.919 g/kg body weight which was more than that of sorafenib (14.244 g/kg body weight). In addition, all values of maximum tolerated dose, rat oral LD_50, and_ rat chronic LOAE of the tested compound were higher than of the reference drug. Furthermore, such compounds were expected to be non-irritant for skin with mild irritants for the eye (Table 6). The four compounds were predicted to have minimum toxicity. All compounds were predicted to be non-carcinogenic and non-mutagenic with

**Table 6.** Toxicity study of the designed compounds.

Comp.	FDA rodent carcinogenicity (Mouse- Female)^a^	Ames prediction^b^	Carcinogenic potency TD_50_ (Rat)^c^	Rat maximum tolerated dose (Feed)^d^	Rat oral LD_50_^d^	Rat chronic LOAEL^d^	Skin irritancy	Ocular irritancy
**A-1**	×	×	19.559	0.103462	2.546	0.331	None	Mild
**B-1**	×	×	108.919	0.114862	1.593	0.515	None	Mild
**C-6**	×	×	75.514	0.254838	1.981	0.283	None	Mild
**D-1**	×	×	65.876	0.197452	1.605	0.259	None	Mild
**Sorafenib**	×	×	14.244	0.088543	0.823	0.005	None	Mild

^a^Non-carcinogen (×) or carcinogen (√).

^b^Non-mutagen (×) or mutagen (√).

^c^Unit: mg/kg body weight/day.

^d^Unit: g/kg body weight.

### 2.2. Chemistry

**Scheme 1.**
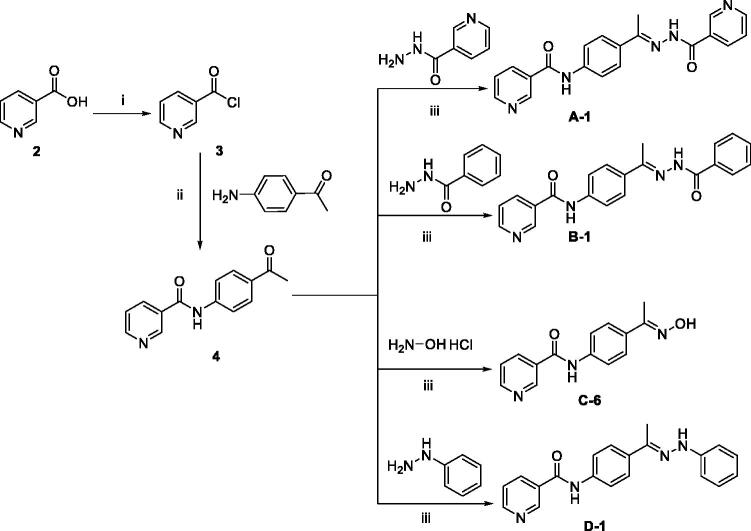
General procedure for the synthesis of target compounds **A-1**, **B-1, C-6,** and **D-1**; Reagents and conditions: (i) Thionyl chloride (SOCl_2_), dichloroethane, reflux, 2 h, (ii) 4-aminoacetophenone, triethylamine (TEA), acetonitrile, stirring, rt, (iii) absolute ethanol, few drops g. acetic acid, reflux, 6 h.

The pyridine-based derivatives **A-1, B-1, C-6,** and **D-1** were synthesised according to the reactions illustrated in Scheme 1. Nicotinic acid **2** underwent a chlorination reaction with thionyl chloride to give nicotinoyl chloride **3**^21^. Nicotinoyl chloride **3** was then reacted with 4-aminoacetophenone to afford *N*-(4-acetylphenyl)nicotinamide **4**. Compound **4** was then condensed with different amino-containing derivatives namely, nicotinohydrazide, benzohydrazide, hydroxylamine, and phenylhydrazine to give the final derivatives **A-1, B-1, C-6, and D-1**, respectively.

Spectral analyses for the synthesised compounds confirmed their structures. The ^1^H NMR of the new derivatives revealed the presence of a single signal at a range of *δ* 2.15–2.39 ppm corresponding to the CH_3_ group found in all derivatives. Additionally, ^1^H NMR of congeners **A-1**, **B-1, and D-1** exhibited the appearance of two NH proton singlet signals for each member at a range of 9.25–10.95 ppm. While the presence of an OH signal at *δ* 11.20 ppm confirmed the structure of compound **C-6**. On the other hand, ^13 ^C NMR spectra of each derivative showed a characteristic upfield peak ranging from *δ* 11.89 to 14.87 ppm corresponding to the methyl group carbon (Scheme 1).

### 2.3. Biological testing

#### 2.3.1. *In vitro* anti-proliferative activity against HepG-2 and HCT-116

The *in vitro* anti-proliferative effects of the four synthesised members were assessed against two cancer cell lines namely, hepatocellular carcinoma (HepG-2) and colorectal carcinoma (HCT-116) cell lines, by the standard MTT test^29^. HepG-2 and HCT-116 cell lines were precisely selected relying on their VEGF overexpression. Sorafenib, a potent TK inhibitor anticancer drug, was also assayed as a positive control. The cytotoxicity results were established in Table 7.

**Table 7.** *In vitro* cytotoxic activities of the assessed compounds against HCT-116 and HepG-2 cell lines and their inhibitory effects VEGFR-2 TK.

Comp. ID	*In vitro* cytotoxicity IC_50_ (µM)		VEGFR-2 protein concentration (nM)
	HCT-116	HepG-2	
A-1	34.9 ± 0.084	18.07 ± 0.052	22.05
B-1	19.6 ± 0.052	17.4 ± 0.05	79.99
C-6	21.8 ± 0.057	15.9 ± 0.045	15.65
D-1	3.08 ± 0.002	4.09 ± 0.005	23.13
Sorafenib	7.28 ± 0.58	5.28 ± 0.21	24.93

Results of the performed test revealed that compound **D-1** possessed a supreme cytotoxic effect against the tested cell lines. Its cytotoxic activity against HCT-116 cells (IC_50_ = 3.08 µM) was almost double that of sorafenib (IC_50_ = 7.28 µM), while, the compound’s activity against HepG-2 (IC_50_ = 4.09 µM) was about 1.2-fold more than sorafenib (IC_50_ = 5.28 µM). Compounds **A-1, B-1,** and **C-6** exhibited moderate cytotoxic activities compared to sorafenib with IC_50_ values ranging from 19.6 to 34.9 µM for HCT-116 cells and 15.9–18.07 µM regarding HepG-2 cells.

Comparing the *in vitro* anti-proliferative activity of compound **D-1** (the most active member) with the previously published lead compounds^21^, indicated that compound **D-1** showed a higher cytotoxic effect against HCT-116 and HepG-2. The cytotoxicity of our published compounds were ranging from 1.94 to 31.70 µM against HCT-116 and from 2.23 to 31.45 µM against HepG-2. The produced activity by the current compounds is higher than most of the previously published ones except compound **A-1**.

#### 2.3.2. Assessment of VEGFR-2 inhibition

The designed congeners were, also, subject to an *in vitro* investigation against VEGFR-2 in HCT-116 cells. Sorafenib was used as a reference drug. HCT-116 cells were treated with the synthesised compounds in concentrations equal to their cytotoxic ones. The VEGFR-2 inhibitory IC_50_ values of the synthesised compounds were summarised in Table 7. Compounds **A-1**, **C-6**, and **D-1** were noticed to possess low IC_50_ values (22.05, 15.65, and 23.13 nM, respectively) which were close to that of sorafenib (24.93 µM) referring to their high activities. While member **B-1** showed a moderate activity with an IC_50_ value of 79.99 nM.

It is worth mentioning that compounds **A-1** and **C-6** showed good VEGFR-2 inhibitory activities but showed fewer anti-proliferative activities. the decreased anti-proliferative activities of these compounds may be attributed to the low hydrophobicity since compound **A-1** has a pyridine moiety that has less hydrophobic characters compared to the phenyl ring of compound **D-1**. Additionally, compound **C-6** has a terminal hydroxyl amine which increases the hydrophilicity of this compound.

#### 2.3.3. Apoptotic markers analysis

##### 2.3.3.1. Assessment of caspase-8, Bax and Bcl-2 expression

Since one of the most important pathways for the anticancer agent to exert its effects is the induction of apoptosis, the most active compound **D-1** was herein tested against apoptosis-related genes including proapoptotic genes, caspase-8, and BAX, and the anti-apoptotic gene Bcl2^30^. Compound **D-1** markedly increased the levels of caspase-8 and BAX expression by 10.41-fold and 9.52-fold, respectively. On the other side, it significantly decreased the anti-apoptotic Bcl-2 level to 0.23-fold (Table 8). Upregulation of the proapoptotic genes in addition to the downregulation of the anti-apoptotic ones, thus, confirmed the apoptotic behaviour of the designed compound.

**Table 8.** Caspase-8 concentrations, BAX, Bcl-2 expression levels in treated HCT-116 cells with the tested compound.

Comp. ID.	BAX (Pg/mL)	Bcl-2 (Pg/mL)	Caspase-8 (Pg/mL)
**D-1**	401.79	1.274	523.61
Control	42.19	5.603	50.317

##### 2.3.3.2. Cell cycle analysis

**Figure 9.**
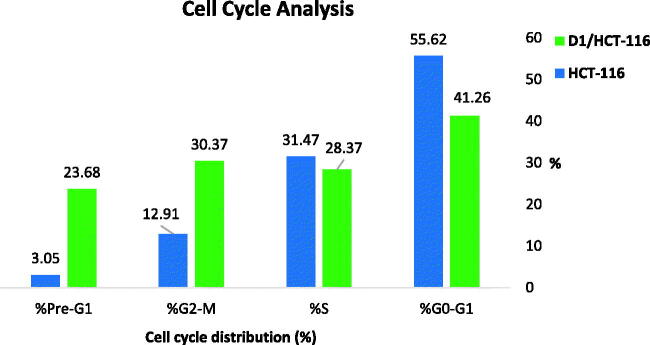
Graphical illustration for the effect of **D-1** on the different % of cell cycle phases in HCT-116 cells.

**Figure 10.**
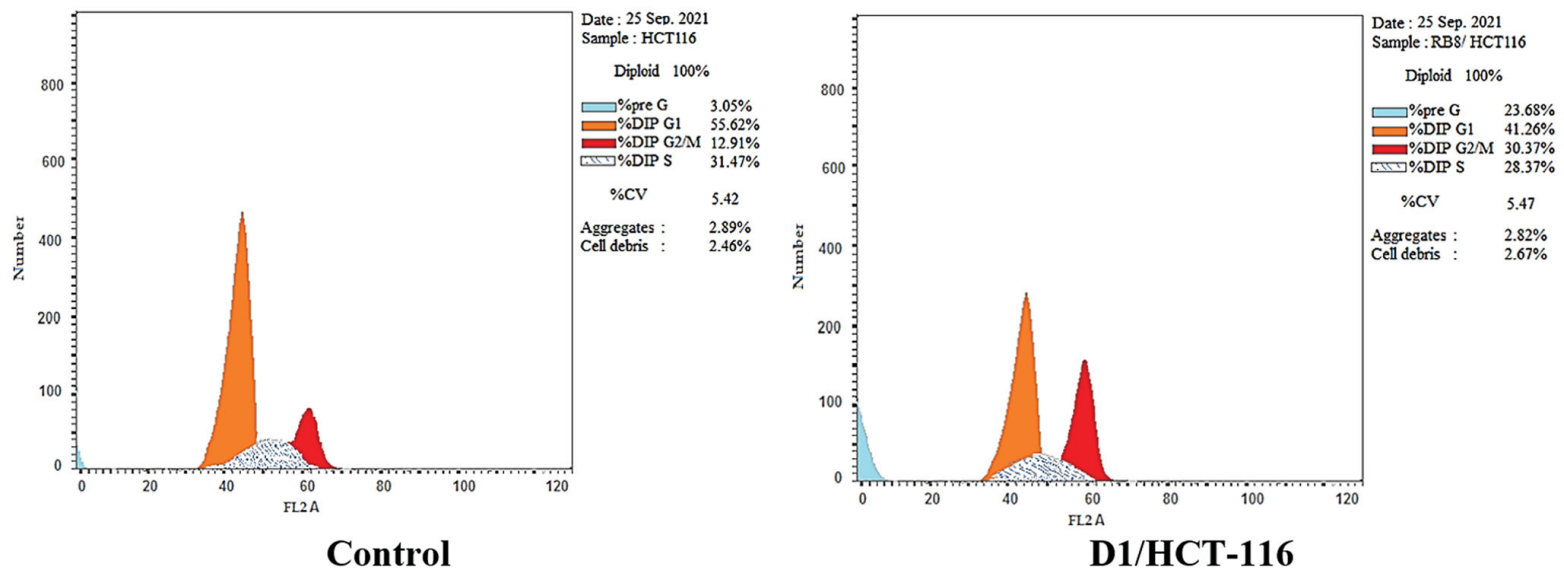
Cell cycle analysis of HCT-116 cells treated with compound **D-1**.

Aiming to obtain a further understanding of the mechanism that triggers the anticancer activity of **D-1**, the cell cycle progression was investigated using flow cytometry in HCT-116 cells. The effects of **D-1** on the cell cycle and the percentage of cells in each phase are demonstrated in Table 9 and Figures 9 and 10.

**Table 9.** Flow cytometry analysis for cell cycle distribution of HCT-116 cells treated with **D-1** in a concentration of 3.08 µM for 48 h.

Sample	%G0-G1	%S	%G2-M	%Pre-G1
D1/HCT-116	41.26	28.37	30.37	23.68
HCT-116	55.62	31.47	12.91	3.05

The obtained data indicated that **D-1** causes a dramatic elevation of the apoptotic cells at the pre-G1 phase (23.68%) compared to that of the control HCT-116 cells (3.05%). Additionally, accumulation of cells was also noticed at the G2-M phase for **D-1** to be 30.37% versus 12.91% accumulation for control HCT-116 cells. The later results were accompanied by a reduction of the S phase percentage of the treated cells (28.37%) in comparison to the untreated cells. Such findings indicate the high activity of compound **D-1** to arrest HCT-116 at both Pre-G1 and G2-M phases.

##### 2.3.3.3. Detection of apoptosis

**Figure 11.**
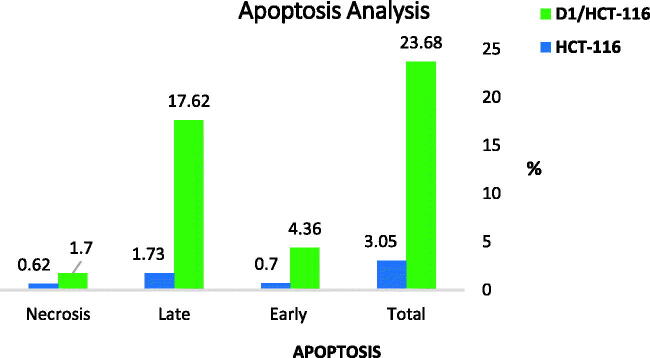
% of apoptotic and necrotic cells among control (HCT-116) cells and compound **D-1** treated cells.

**Figure 12.**
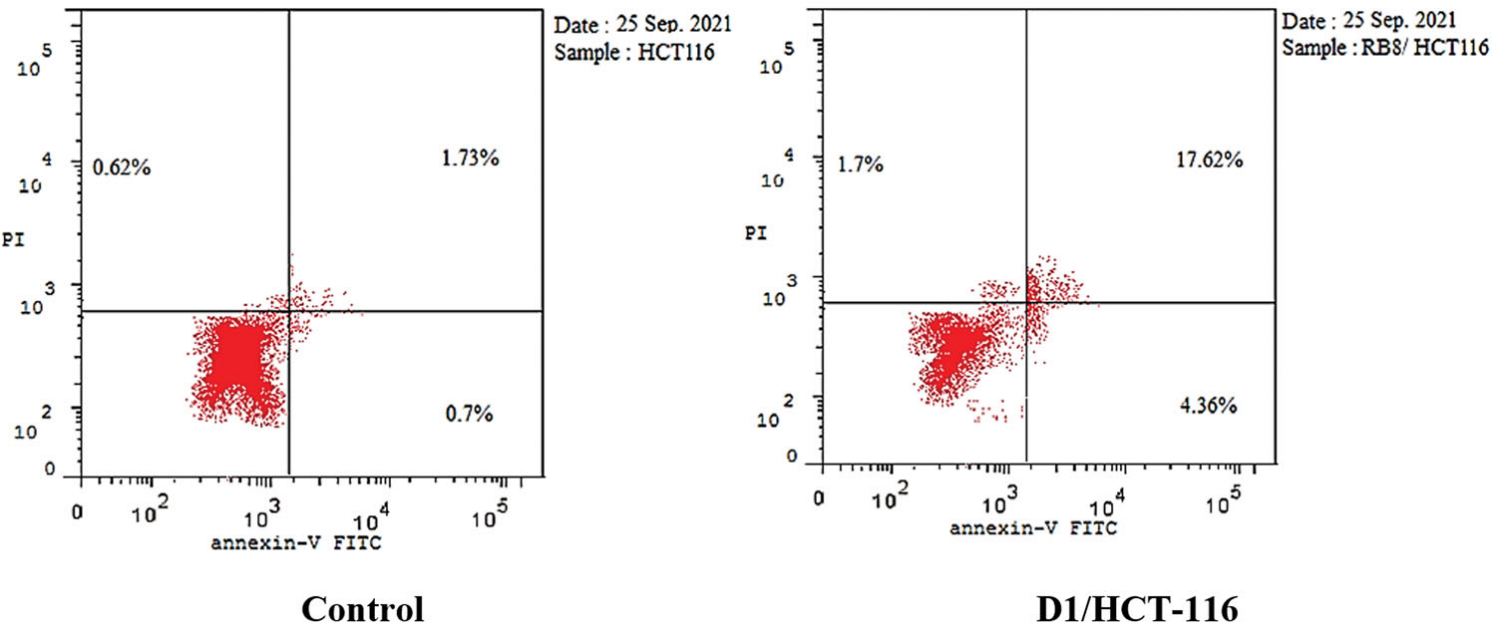
Compound **D-1** induces apoptosis in HCT-116 cells.

Since the induction of apoptosis is a key determinant in the drug’s therapeutic outcome, the ability of the best cytotoxic member **D-1** to induce apoptosis in colorectal carcinoma (HCT-116) cells was evaluated using Annexin-V/propidium iodide (PI) staining assay. Compound **D-1** was observed to induce apoptosis in both early and late apoptotic stages in a dose equivalent to its cytotoxic IC_50_ value. The percentage of early and late apoptotic populations elevated from 0.7% and 1.73% in the untreated cells to 4.36% and 17.62% in the **D-1** treated cells (Table 10 and Figures 11 and 12).

**Table 10.** Induced apoptosis in HCT-116 cells treated with **D-1** in a concentration of 3.08 µM for 48 h.

Sample	Apoptosis			Necrosis
	Total	Early	Late	
D1/HCT-116	23.68	4.36	17.62	1.7
HCT-116	3.05	0.7	1.73	0.62

#### 2.3.4. *In vitro* immunomodulatory assay

Compounds **C-6** and **D-1** were further assayed for their immunomodulatory effect on HCT-116 cells. Two immunity-related markers were measured namely, human tumour necrosis factor alpha (TNF-*α*), and interleukin 6 (IL6). Dexamethasone, a potent inhibitor of both TNF-*α* and IL6, was used as a positive control. The obtained results revealed that compound **D-1** strongly decreased the TNF-*α* and IL6 levels by 91.19% and 85.64%, respectively. While compound **C-6** inhibited TNF-*α* by 86.48% and IL6 by 75.21% (Table 11).

**Table 11.** TNF**-***α* and IL6 percent inhibition in treated HCT-116 cells with the tested compounds **C-6** (21.8 µM) and **D-1** (3.08 µM) for 48 h.

Comp.	% Inhibition of TNF-α	% Inhibition of IL6
**C-6**	86.48	75.21
**D-1**	91.19	85.64
Dexamethasone	82.47	93.15

### 2.4. Molecular dynamic simulation

Molecular dynamics (MD) simulations methods almost become a usual computational procedure in drug design as well as drug discovery^31^. The major two strength points of MD methods are first, its accurate ability to track both entropic and structural changes in the ligand as well as the target enzyme. Also, those changes were tracked for ligand and enzyme over a specific time every very-short period at an extremely high resolution of atomic level^32^. In consequence, MD methods can precisely calculate the changes that resulted from the ligand-protein binding in the kinetics and thermodynamics levels^33^. The mentioned strengths represented the MD as an efficient tool to identify the nature of the structure-function of the examined ligand-protein complex. It reveals essential factors such as the stability of the examined complex, ligand binding free energy, and kinetics^34^.

**Figure 13.**
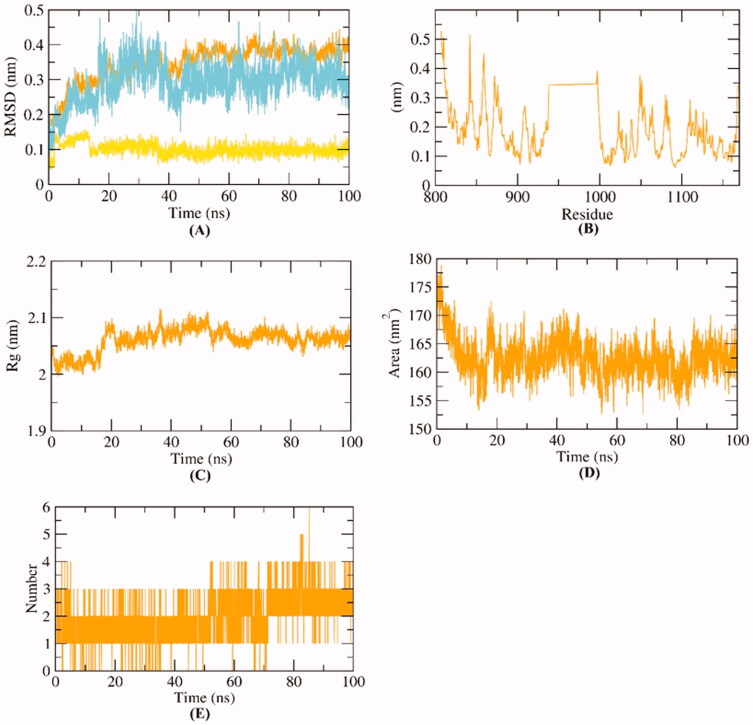
MD simulations experiment: (A) RMSD values of **D-1**-VEGFR-2 complex before and after binding, (B) RMSF of **D-1**-VEGFR-2 complex (C) R_g_ of **D-1**-VEGFR-2 complex (D) SASA of **D-1**-VEGFR-2 complex, (E) H- bonding between **D-1**-VEGFR-2 complex.

We here in reported several MD simulations studies for compound **D-1**-VEGFR-2 complex. At first, the conformational changes that occurred in the **D-1**-VEGFR-2 complex after binding were investigated for **D-1**, VEGFR-2, and **D-1**-VEGFR-2 complex through the calculation of RMSD values over 100 ns in atomic resolution (Figure 13A). It was observed that VEGFR-2, **D-1** and the **D-1**-VEGFR-2 complex exhibited low RMSD values without major fluctuations. Although the complex has slightly fluctuated till 40 ns∼, it got stabled later. Such results indicate the great stability of the **D-1**-VEGFR-2 complex. Secondly, the flexibility of VEGFR-2 was examined in terms of RMSF. The results (Figure 13B) indicated that **D-1** binding to VEGFR-2 didn’t cause dramatic changes in the flexibility. Followingly, the compactness of the **D-1**-VEGFR-2 complex was indicated by exhibiting low values of radius of gyration (Rg) indicating a lower degree of fluctuation and greater compactness of the **D-1**-VEGFR-2 complex (Figure 13C). Figure 13D shows the calculated values of solvent accessible surface area (SASA). SASA examined the interaction between **D-1**-VEGFR-2 and solvents was over 100 ns to analyse the degree of conformational changes in VEGFR-2 after D-1 binding. Interestingly, VEGFR-2 featured a reduction of SASA values at the end of simulations than the starting period indicating a lower degree of conformational changes and more stability. The hydrogen bonding in the **D-1**-VEGFR-2 complex was computed over 100 ns and the highest number of hydrogen bonds between VEGFR-2 and D-1was four (Figure 13E).

#### 2.4.1. Molecular mechanics poisson–boltzmann surface area (MM-PBSA)

**Figure 14.**
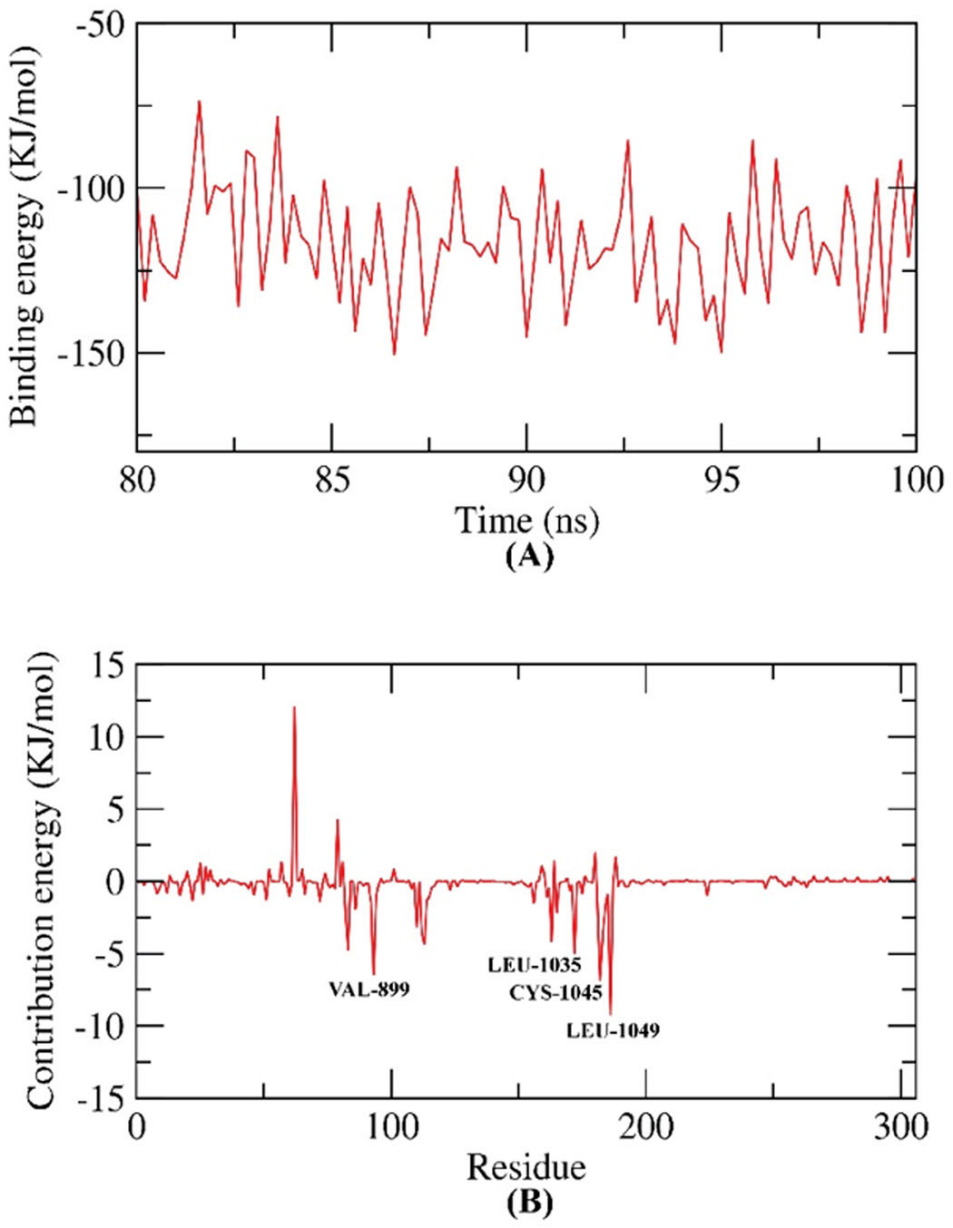
MM-PBSA study of **D-1-**VEGFR-2 complex.

The Molecular Mechanics Poisson–Boltzmann surface area (MM-PBSA) assay was applied to estimate the free binding energy of the **D-1**-VEGFR-2 complex on a dynamic base. The MM-PBSA has several advantages over other analysis methods that are used for the same purpose as thermodynamic integration and the free energy perturbation. These advantages are being simpler, faster, and producing uniform results^35^. The binding free energy of the **D-1**-VEGFR-2 complex was estimated at the final stable 20 ns of the MD experiment run with a time interval of 100 ps. The MM/PBSA method, as well as MmPbSaStat.py script, were utilised to investigate the average free binding energy in addition to its standard deviation/error (SD). Compound **D-1** exhibited a low binding free energy average of −124 KJ/mol with the VEGFR-2 (Figure 14A). The binding energy of the examined **D-1**-VEGFR-2 complex was stable through the 20 ns of examination.

#### 2.4.2. Free energy decomposition

Followingly, the total binding free energy of the **D-1**-VEGFR-2 complex was analysed (decomposed) to figure out the different components of the obtained binding energy. In addition, to explore the participation of each amino acid residue of the VEGFR-2 in the binding with compound **D-1**. This experiment revealed the basic amino acid residues with an advantageous contribution to the binding. The following amino acid residues (VAL-899, LEU-1035, CYS-1045, and LEU-1049) of VEGFR-2 contributed higher than −7 KJ/mol binding energy and believed as vital residues in the binding with compound **D-1** (Figure 14B).

## 3. Conclusion

In a recent study, the ability of four compounds to inhibit the VEGFR-2 enzyme was emphasised. The tested compounds were selected and synthesised after a computational screening of four corresponding series. Cytotoxic evaluation of the synthesised members was performed against HepG-2 and HCT-116 cell lines. *In vitro* VEGFR-2 inhibitory assay was, additionally, carried out for the four compounds. Congener **D-1** was the most potent cytotoxic member with IC_50_ values of 4.09 µM against HepG-2 cells and 3.08 µM regarding HCT-116 cells. Compound **D-1** inhibited VEGFR-2 enzyme at a concentration of 23.13 nM. The capability of compound **D-1** to induce apoptosis was then assayed. It caused a significant elevation of both caspase-8 and BAX expression with 10.41-fold and 9.52-fold, respectively, in comparison to the reference drug. While it decreased Bcl-2 level to 0.23-fold. Compound **D-1** arrested cell growth in HCT-116 cells at G2-M and pre-G1 phases *via* accumulation of cells by 30.37% with induction of apoptosis. The MD simulation revealed that compound **D-1** has the greatest potential to fit in the active site of VEGFR-2.

## 4. Materials and methods

### 4.1. Virtual screening

#### 4.1.1. Docking studies

Computational virtual screening for the four sets of compounds was performed using MOE.14 software against VEGFR-2 TK (PDB ID: 4ASD, resolution: 2.05 Å)^19^ using as shown in Supplementary data.

#### 4.1.2. ADME studies

ADME studies were carried out using Discovery studio 4.0 following the reported procedure^36^^,^^37^ (Supplementary data).

#### 4.1.3. Toxicity studies

The toxicity parameters of the four congeners were calculated using Discovery studio 4.0 as described in Supplementary data.

### 4.2. Chemistry

#### 4.2.1. General

All the reagents, chemicals, and apparatus were described in Supplementary data. Compounds **3 and 4** were prepared according to the reported procedures^21^.

#### 4.2.2. General procedure for preparation of the target compounds A-1, B-1, C-6, and D-1

A mixture of *N*-(4-acetylphenyl)nicotinamide **4** (0.24 g, 0.001 mol) and the appropriate amino-containing derivatives namely, nicotinohydrazide, benzohydrazide, hydroxylamine, and phenylhydrazine (0.001 mol) was refluxed in absolute ethanol (25 ml) in the presence of few drops glacial acetic acids for 6 h. Following, the mixture was cooled to room temperature then, the formed precipitate was filtered, dried, and recrystallized from ethanol.

##### 4.2.2.1. N-(4–(1-(2-Nicotinoylhydrazineylidene)ethyl)phenyl)nicotinamide A-1

Yield: 76%; Melting point: 240–242 °C; IR υ_max_/cm^−1^: 3348, 1648, 1597; ^1^H NMR (DMSO-*d*6, 400 MHz) *δ* ppm: 2.39 (s, 3H), 7.54–7.60 (m, 2H), 7.90 (m, 4H), 8.23 (d, *J* = 7.6 Hz, 1H), 8.31 (d, *J* = 7.6 Hz, 1H), 8.77 (d, *J* = 7.6 Hz, 2H), 9.05 (s, 1H), 9.13 (s, 1H), 10.61 (s, 1H), 10.95 (s, 1H); ^13 ^C NMR (DMSO-*d*6) *δ* (ppm): 14.43, 120.29 (2C), 123.98 (2C), 127.17 (2C), 130.94, 133.78, 135.99 (2C), 136.16 (2C), 140.67, 147.75 (2C), 152.45, 152.68 (2C), 164.66.

##### 4.2.2.2. N-(4–(1-(2-Benzoylhydrazineylidene)ethyl)phenyl)nicotinamide B-1

Yield: 73%; Melting point: 258–260 °C; IR υ_max_/cm^−1^: 3269, 1667, 1599; ^1^H NMR (DMSO-*d*6, 400 MHz) *δ* ppm: 2.39 (s, 3H), 7.53 (d, *J* = 7.6 Hz, 2H), 7.59 (d, *J* = 8.0 Hz, 2H), 7.90 (m, 6H), 8.34 (d, *J* = 8.0 Hz, 1H), 8.79 (s, 1H), 9.05 (s, 1H), 10.64 (s, 1H), 10.79 (s, 1H); ^13 ^C NMR (DMSO-*d*6) *δ* (ppm): 14.87, 120.30 (2C), 123.99 (2C), 127.48, 128.33, 128.81, 129.84, 130.96, 131.95, 133.95, 134.61, 136.00 (2C), 140.55, 149.20 (2C), 152.67, 155.73, 164.67.

##### 4.2.2.3. N-(4–(1-(Hydroxyimino)ethyl)phenyl)nicotinamide C-6

Yield: 69%; Melting point: 243–245 °C; IR υ_max_/cm^−1^: 3450, 3298, 3187, 1676, 1599; ^1^H NMR (DMSO-*d*6, 400 MHz) *δ* ppm: 2.15 (s, 3H), 7.67 (d, *J* = 8.8 Hz, 2H), 7.87 (d, *J* = 8.8 Hz, 2H), 8.07 (dd, *J* = 7.6, 7.6 Hz, 1H), 8.96 (d, *J* = 8.4 Hz, 1H), 9.02 (d, *J* = 5.2 Hz, 1H), 9.47 (s, 1H), 10.40 (s, 1H), 11.20 (s, 1H); ^13 ^C NMR (DMSO-*d*6) *δ* (ppm): 11.89, 120.60 (2C), 126.42 (2C), 126.67, 133.13, 133.30, 139.36, 143.19, 143.78, 146.19, 152.87, 162.02.

##### 4.2.2.4. N-(4–(1-(2-Phenylhydrazineylidene)ethyl)phenyl)nicotinamide D-1

Yield: 70%; Melting point: 235–237 °C; IR υ_max_/cm^−1^: 3248, 1648, 1597; ^1^H NMR (DMSO-*d*6, 400 MHz) *δ* ppm: 2.27 (s, 3H), 6.76 (t, *J* = 6.8 Hz, 1H), 7.21–7.28 (m, 4H), 7.58 (dd, *J* = 7.6, 8.0 Hz, 1H), 7.83 (m, 4H), 8.32 (d, *J* = 8.0 Hz, 1H), 8.78 (d, 1H), 9.14 (s, 1H), 9.25 (s, 1H), 10.54 (s, 1H); ^13 ^C NMR (DMSO-*d*6) *δ* (ppm): 13.18, 113.27 (2C), 119.23, 120.50 (2C), 123.98, 125.98 (2C), 129.37 (2C), 130.78, 135.35, 135.96, 138.77, 140.76, 146.62, 149.17, 152.57, 164.46.

### 4.3. Biological testing

#### 4.3.1. *In vitro* anti-proliferative activity against HepG-2 and HCT-116

The anti-proliferative activity of the four members was estimated by the MTT protocol as described^38–41^ (Supplementary data).

#### 4.3.2. Assessment of VEGFR-2 inhibition

The four selected members were further assessed to measure their inhibitory activities against the VEGFR-2 enzyme following the protocol shown in Supplementary data.

#### 3.3.3. Apoptotic markers analysis

##### 4.3.3.1. Assessment of the expression of caspase-8, Bax and Bcl-2

Estimation of caspase-3 levels after treatment of cells with compound **D-1** was performed according to the protocol described by M. Andersson et al.^42^. While Bax and Bcl-2 cellular levels were evaluated for **D-1** in HCT-116 cells according to the reported protocol^43^ (Supplementary data).

##### 4.3.3.2. Cell cycle analysis

Based on the protocol referred to by Léonce *et al*., the flow cytometric analysis for congener **D-1** was done^19^^,^^44–46^ (Supplementary data).

##### 4.3.3.3. Detection of apoptosis

Annexin‐V‐FITC assay for compound **D-1** on HCT-116 cells according to the reported procedure^15^^,^^47–49^ (Supplementary data).

#### 4.3.4. *In vitro* immunomodulatory assay

The level of TNF-α and IL6 in cell culture supernatants was assessed by the ELISA technique according to the reported procedure^50^^,^^51^.

### 4.4. Molecular dynamic (MD) simulation and MM/PBSA

MD simulation experiments and MM/PBSA (Molecular Mechanics/Poisson Boltzmann Surface Area) were carried out using GROMACS as reported in Supplementary data^52–55^.

## Supplementary Material

Supplemental Material

## References

[1] Liang P, Ballou B, Lv X, et al. Monotherapy and combination therapy using anti‐angiogenic nanoagents to fight cancer. Adv Mater 2021;33:1389. 10.1002/adma.202005155 33684242

[2] Folkman J. Angiogenesis in cancer, vascular, rheumatoid and other disease. Nat Med 1995;1:27–30. 7584949 10.1038/nm0195-27

[3] Kerbel RS. Tumor angiogenesis: past, present and the near future. Carcinogenesis 2000;21:505–15. 10688871 10.1093/carcin/21.3.505

[4] El-Adl K, El-Helby A-GA, Ayyad RR, et al. Design, synthesis, and anti-proliferative evaluation of new quinazolin-4(3H)-ones as potential VEGFR-2 inhibitors. Bioorg Med Chem 2021;29:115872 33214036 10.1016/j.bmc.2020.115872

[5] Eissa IH, El-Helby A-GA, Mahdy HA, et al. Discovery of new quinazolin-4(3H)-ones as VEGFR-2 inhibitors: design, synthesis, and anti-proliferative evaluation. Bioorg Chem 2020;105:104380. 33128967 10.1016/j.bioorg.2020.104380

[6] Veeravagu A, Hsu AR, Cai W, et al. Vascular endothelial growth factor and vascular endothelial growth factor receptor inhibitors as anti-angiogenic agents in cancer therapy. Recent Pat Anticancer Drug Discov 2007;2:59–71. 18221053 10.2174/157489207779561426

[7] Mahdy HA, Ibrahim MK, Metwaly AM, et al. Design, synthesis, molecular modeling, in vivo studies and anticancer evaluation of quinazolin-4(3H)-one derivatives as potential VEGFR-2 inhibitors and apoptosis inducers. Bioorg Chem 2020;94:103422. 31812261 10.1016/j.bioorg.2019.103422

[8] Kang D, Pang X, Lian W, et al. Discovery of VEGFR2 inhibitors by integrating naïve Bayesian classification, molecular docking and drug screening approaches. RSC Adv 2018;8:5286–97. 35542432 10.1039/c7ra12259d PMC9078101

[9] Zhong H-T, Yu Y-Z, Velasco C. Molecular insights probing Bismurrayaquinone A as an angiogenesis inhibitor via inhibition of VEGFR-2 Kinase domain. Citeseer 2011;1(4):95–100.

[10] Lugano R, Ramachandran M, Dimberg A. Tumor angiogenesis: causes, consequences, challenges and opportunities. Cell Mol Life Sci 2020;77:1745–70. 31690961 10.1007/s00018-019-03351-7 PMC7190605

[11] Hunter T. The role of tyrosine phosphorylation in cell growth. In: The Harvey Lectures Series 94, 1998–1999. Vol. 94; 2000:81. 11070953

[12] Claesson‐Welsh L, Welsh M. VEGFA and tumour angiogenesis. J Intern Med 2013;273:114–27. 23216836 10.1111/joim.12019

[13] Eissa IH, Ibrahim MK, Metwaly AM, et al. Design, molecular docking, *in vitro*, and *in vivo* studies of new quinazolin-4(3H)-ones as VEGFR-2 inhibitors with potential activity against hepatocellular carcinoma. Bioorg Chem 2021;107:104532. 33334586 10.1016/j.bioorg.2020.104532

[14] El-Metwally SA, Abou-El-Regal MM, Eissa IH, et al. Discovery of thieno[2,3-d]pyrimidine-based derivatives as potent VEGFR-2 kinase inhibitors and anti-cancer agents. Bioorg Chem 2021;112:104947. 33964580 10.1016/j.bioorg.2021.104947

[15] Alanazi MM, Mahdy HA, Alsaif NA, et al. New bis([1,2,4]triazolo)[4,3-a:3',4'-c]quinoxaline derivatives as VEGFR-2 inhibitors and apoptosis inducers: Design, synthesis, in silico studies, and anticancer evaluation. Bioorg. Chem 2021;112:104949. 34023640 10.1016/j.bioorg.2021.104949

[16] El-Adl K, Sakr HM, Yousef RG, et al. Discovery of new quinoxaline-2(1H)-one-based anticancer agents targeting VEGFR-2 as inhibitors: design, synthesis, and anti-proliferative evaluation. Bioorg Chem 2021;114:105105. 34175720 10.1016/j.bioorg.2021.105105

[17] Yousef RG, Sakr HM, Eissa IH, et al. New quinoxaline-2 (1 H)-ones as potential VEGFR-2 inhibitors: design, synthesis, molecular docking, ADMET profile and anti-proliferative evaluations. N J Chem 2021;45:16949–64.

[18] Parmar DR, Soni JY, Guduru R, et al. Discovery of new anticancer thiourea-azetidine hybrids: design, synthesis, in vitro antiproliferative, SAR, in silico molecular docking against VEGFR-2, ADMET, toxicity, and DFT studies. Bioorg Chem 2021;115:105206. 34339975 10.1016/j.bioorg.2021.105206

[19] Alanazi MM, Eissa IH, Alsaif NA, et al. Design, synthesis, docking, ADMET studies, and anticancer evaluation of new 3-methylquinoxaline derivatives as VEGFR-2 inhibitors and apoptosis inducers. J Enzyme Inhib Med Chem 2021;36:1760–82. 34340610 10.1080/14756366.2021.1956488 PMC8344243

[20] Alanazi MM, Elwan A, Alsaif NA, et al. Discovery of new 3-methylquinoxalines as potential anti-cancer agents and apoptosis inducers targeting VEGFR-2: design, synthesis, and in silico studies. J Enzyme Inhib Med Chem 2021;36:1732–50. 34325596 10.1080/14756366.2021.1945591 PMC8330740

[21] Ran F, Li W, Qin Y, et al. Inhibition of vascular smooth muscle and cancer cell proliferation by new VEGFR inhibitors and their immunomodulator effect: design, synthesis, and biological evaluation. Oxid Med Cell Longev 2021;2021:1–21. 10.1155/2021/8321400 PMC8568530 34745424

[22] Oguro Y, Cary DR, Miyamoto N, et al. Design, synthesis, and evaluation of novel VEGFR2 kinase inhibitors: discovery of [1,2,4]triazolo[1,5-a]pyridine derivatives with slow dissociation kinetics. Bioorg Med Chem 2013;21:4714–29. 23755884 10.1016/j.bmc.2013.04.042

[23] AbdelHaleem A, Mansour AO, AbdelKader M, Arafa RK. Selective VEGFR-2 inhibitors: synthesis of pyridine derivatives, cytotoxicity and apoptosis induction profiling. Bioorg Chem 2020;103:104222. 32889383 10.1016/j.bioorg.2020.104222

[24] Gu W, Dai Y, Qiang H, et al. Discovery of novel 2-substituted-4-(2-fluorophenoxy) pyridine derivatives possessing pyrazolone and triazole moieties as dual c-Met/VEGFR-2 receptor tyrosine kinase inhibitors. Bioorg Chem 2017;72:116–22. 28411406 10.1016/j.bioorg.2017.04.001

[25] Lee K, Jeong K-W, Lee Y, et al. Pharmacophore modeling and virtual screening studies for new VEGFR-2 kinase inhibitors. Eur J Med Chem 2010;45:5420–7. 20869793 10.1016/j.ejmech.2010.09.002

[26] Machado VA, Peixoto D, Costa R, et al. Synthesis, antiangiogenesis evaluation and molecular docking studies of 1-aryl-3-[(thieno[3,2-b]pyridin-7-ylthio)phenyl]ureas: discovery of a new substitution pattern for type II VEGFR-2 Tyr kinase inhibitors. Biorg. Med. Chem 2015;23:6497–509. 10.1016/j.bmc.2015.08.010 26344591

[27] Garofalo A, Goossens L, Six P, et al. Impact of aryloxy-linked quinazolines: a novel series of selective VEGFR-2 receptor tyrosine kinase inhibitors. Bioorg Med Chem Lett 2011;21:2106–12. 21353546 10.1016/j.bmcl.2011.01.137

[28] Xia X, Maliski EG, Gallant P, Rogers D. Classification of kinase inhibitors using a Bayesian model. J Med Chem 2004;47:4463–70. 15317458 10.1021/jm0303195

[29] Mosmann T. Rapid colorimetric assay for cellular growth and survival: application to proliferation and cytotoxicity assays. J Immunol Methods 1983;65:55–63. 6606682 10.1016/0022-1759(83)90303-4

[30] Cai SX, Nguyen B, Jia S, et al. Discovery of substituted N-phenyl nicotinamides as potent inducers of apoptosis using a cell- and caspase-based high throughput screening assay. J Med Chem 2003;46:2474–81. 12773051 10.1021/jm0205200

[31] Sousa SF, Fernandes PA, Ramos MJ. Protein-ligand docking: current status and future challenges. Proteins: Struct Funct Bioinform 2006;65:15–26. 10.1002/prot.21082 16862531

[32] Hollingsworth SA, Dror RO. Molecular dynamics simulation for all. Neuron 2018;99:1129–43. 30236283 10.1016/j.neuron.2018.08.011 PMC6209097

[33] Hansson T, Oostenbrink C, van Gunsteren W. Molecular dynamics simulations. Curr Opin Struct Biol 2002;12:190–6. 11959496 10.1016/s0959-440x(02)00308-1

[34] Durrant JD, McCammon JA. Molecular dynamics simulations and drug discovery. BMC Biol 2011;9:71–9. 22035460 10.1186/1741-7007-9-71 PMC3203851

[35] Ren J, Yuan X, Li J, et al. Assessing the performance of the g_mmpbsa tools to simulate the inhibition of oseltamivir to influenza virus neuraminidase by molecular mechanics Poisson–Boltzmann surface area methods. J Chinese Chem Soc 2020;67:46–53.

[36] El-Zahabi MA, Elbendary ER, Bamanie FH, et al. Design, synthesis, molecular modeling and anti-hyperglycemic evaluation of phthalimide-sulfonylurea hybrids as PPARγ and SUR agonists. Bioorganic Chem 2019;91:103115. 10.1016/j.bioorg.2019.103115 31310882

[37] Ibrahim MK, Eissa IH, Alesawy MS, et al. Design, synthesis, molecular modeling and anti-hyperglycemic evaluation of quinazolin-4(3H)-one derivatives as potential PPARγ and SUR agonists. Bioorg Med Chem 2017;25:4723–44. 28720328 10.1016/j.bmc.2017.07.015

[38] Gerlier D, Thomasset N. Use of MTT colorimetric assay to measure cell activation. J Immunol Methods 1986;94:57–63. 3782817 10.1016/0022-1759(86)90215-2

[39] Denizot F, Lang R. Rapid colorimetric assay for cell growth and survival: modifications to the tetrazolium dye procedure giving improved sensitivity and reliability. J. Immunol. Methods 1986;89:271–7. 3486233 10.1016/0022-1759(86)90368-6

[40] Thabrew MI, HUGHES RD, MCFARLANE IG. Screening of hepatoprotective plant components using a HepG2 cell cytotoxicity assay. J. Pharm. Pharmacol 2011;49:1132–5. 10.1111/j.2042-7158.1997.tb06055.x 9401951

[41] Al-Sanea MM, Al-Ansary GH, Elsayed ZM, et al. Development of 3-methyl/3-(morpholinomethyl)benzofuran derivatives as novel antitumor agents towards non-small cell lung cancer cells. J Enzyme Inhib Med Chem 2021;36:987–99. 33985397 10.1080/14756366.2021.1915302 PMC8128204

[42] Andersson M, Sjöstrand J, Petersen A, et al. Caspase and proteasome activity during staurosporin-induced apoptosis in lens epithelial cells. Invest Ophthalmol Vis Sci 2000;41:2623–32. 10937575

[43] Emily H-YC, Wei MC, Weiler S, et al. BCL-2, BCL-XL sequester BH3 domain-only molecules preventing BAX-and BAK-mediated mitochondrial apoptosis. Mol Cell 2001;8:705–11. 11583631 10.1016/s1097-2765(01)00320-3

[44] Léonce S, Pérez V, Lambel S, et al. Induction of cyclin E and inhibition of DNA synthesis by the novel acronycine derivative S23906-1 precede the irreversible arrest of tumor cells in S phase leading to apoptosis. Mol Pharmacol 2001;60:1383–91. 11723246 10.1124/mol.60.6.1383

[45] Eldehna WM, El Hassab MA, Abo-Ashour MF, et al. Development of isatin-thiazolo[3,2-a]benzimidazole hybrids as novel CDK2 inhibitors with potent *in vitro* apoptotic anti-proliferative activity: synthesis, biological and molecular dynamics investigations Bioorg Chem 2021;110:104748. 33684714 10.1016/j.bioorg.2021.104748

[46] Abbass EM, Khalil AK, Mohamed MM, et al. Design, efficient synthesis, docking studies, and anticancer evaluation of new quinoxalines as potential intercalative Topo II inhibitors and apoptosis inducers. Bioorg Chem 2020;104:104255. 32927130 10.1016/j.bioorg.2020.104255

[47] Lo KK-W, Lee TK-M, Lau JS-Y, et al. Luminescent biological probes derived from ruthenium(II) estradiol polypyridine complexes. Inorg Chem 2008;47:200–8. 18067284 10.1021/ic701735q

[48] Hagras M, El Deeb MA, Elzahabi HS, et al. Discovery of new quinolines as potent colchicine binding site inhibitors: design, synthesis, docking studies, and anti-proliferative evaluation. J Enzyme Inhib Med Chem 2021;36:640–58. 33588683 10.1080/14756366.2021.1883598 PMC7889231

[49] Al-Warhi T, Abo-Ashour MF, Almahli H, et al. Novel [(N-alkyl-3-indolylmethylene)hydrazono]oxindoles arrest cell cycle and induce cell apoptosis by inhibiting CDK2 and Bcl-2: synthesis, biological evaluation and in silico studies. J Enzyme Inhib Med Chem 2020;35:1300–9. 32522063 10.1080/14756366.2020.1773814 PMC7717600

[50] Talaat RM. Soluble angiogenesis factors in sera of Egyptian patients with hepatitis C virus infection: correlation with disease severity. Viral Immunol 2010;23:151–7. 20373995 10.1089/vim.2009.0089

[51] El-Zahabi MA, Sakr H, El-Adl K, et al. Design, synthesis, and biological evaluation of new challenging thalidomide analogs as potential anticancer immunomodulatory agents. Bioorg Chem 2020;104:104218 32932121 10.1016/j.bioorg.2020.104218

[52] Suleimen YM, Jose RA, Suleimen RN, et al. Isolation and *in silico* anti-SARS-CoV-2 papain-like protease potentialities of two rare 2-phenoxychromone derivatives from Artemisia spp. Molecules 2022;27:1216. 35209006 10.3390/molecules27041216 PMC8879996

[53] Jo S, Kim T, Iyer VG, Im W. CHARMM-GUI: a web-based graphical user interface for CHARMM. J Comput Chem 2008;29:1859–65. 18351591 10.1002/jcc.20945

[54] Brooks BR, Brooks CL, III, Mackerell AD Jr, et al. CHARMM: the biomolecular simulation program. J Comput Chem 2009;30:1545–614. 19444816 10.1002/jcc.21287 PMC2810661

[55] Lee J, Cheng X, Swails JM, et al. CHARMM-GUI input generator for NAMD, GROMACS, AMBER, OpenMM, and CHARMM/OpenMM simulations using the CHARMM36 additive force field. J Chem Theory Comput 2016;12:405–13. 26631602 10.1021/acs.jctc.5b00935 PMC4712441

