# Supplementary material for: Discovery of new nicotinamides as apoptotic VEGFR-2 inhibitors: virtual screening, synthesis, anti-proliferative, immunomodulatory, ADMET, toxicity, and molecular dynamic simulation studies
Source: J Enzyme Inhib Med Chem. 2022 May 16;37(1):1389–403. doi: 10.1080/14756366.2022.2070744 (PMC9116259; doi:10.1080/14756366.2022.2070744)
Supplement: Supplemental Material [file IENZ_A_2070744_SM9148.pdf]

**Discovery of new nicotinamides as apoptotic VEGFR-2 inhibitors: virtual screening, synthesis, anti-proliferative, immunomodulatory, ADMET, toxicity, and molecular dynamic simulation studies**

Reda G.Yousef[a], Albaraa Ibrahim[a], Mohamed M. Khalifa[a], Wagdy M. Eldehna[b,c], Ibraheem M. M. Gobaara[d], Ahmed B. M. Mehany[d], Eslam B. Elkaeed[e],Aisha A. Alsfouk[f], Ahmed M. Metwaly[g,h]* Ibrahim. H. Eissa[a]*,

[a] Pharmaceutical Medicinal Chemistry & Drug Design Department, Faculty of Pharmacy (Boys), Al-Azhar University, Cairo11884, Egypt.

[b] School of Biotechnology, Badr University in Cairo, Badr City, Cairo 11829, Egypt

[c]Department of Pharmaceutical Chemistry, Faculty of Pharmacy, Kafrelsheikh University, P.O. Box 33516, Kafrelsheikh, Egypt.

[d] Zoology Department, Faculty of Science (Boys), Al-Azhar University, Cairo11884, Egypt.

[e] Department of Pharmaceutical Sciences, College of Pharmacy, AlMaarefa University, Riyadh 13713, Saudi Arabia.

[f] Department of Pharmaceutical Sciences, College of Pharmacy, Princess Nourah bint Abdulrahman University, P.O. Box 84428, Riyadh 11671, Saudi Arabia.

[g] Pharmacognosy and Medicinal Plants Department, Faculty of Pharmacy (Boys), Al-Azhar University, Cairo 11884, Egypt.

[h]Biopharmaceutical Products Research Department, Genetic Engineering and Biotechnology Research Institute, City of Scientific Research and Technological Applications (SRTA-City), Alexandria, Egypt

***Corresponding authors:**

**Ibrahim H. Eissa**

Medicinal Chemistry Department, Faculty of Pharmacy (Boys), Al-Azhar University, Cairo 11884, Egypt. **Email:** Ibrahimeissa@azhar.edu.eg

**Ahmed M. Metwaly**

Pharmacognosy and Medicinal Plants Department, Faculty of Pharmacy (Boys), Al-Azhar University, Cairo 11884, Egypt. **Email:** ametwaly@azhar.edu.eg

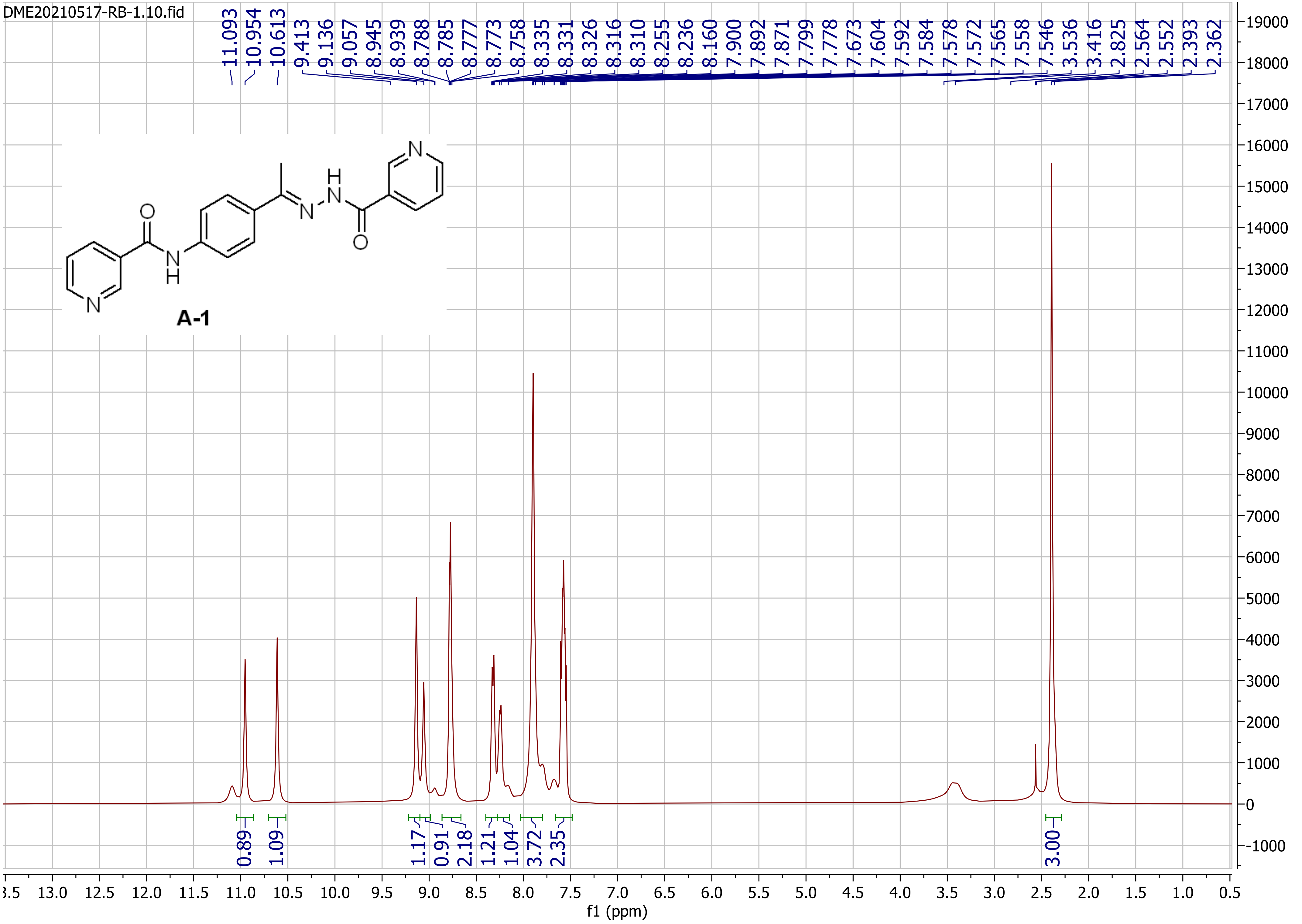
DME20210517-RB-1.10.fid
11.093
10.954
10.613
9.413
9.136
9.057
8.945
8.939
8.788
8.785
8.777
8.773
8.758
8.335
8.331
8.326
8.316
8.310
8.255
8.236
8.160
7.900
7.892
7.871
7.799
7.778
7.673
7.604
7.592
7.584
7.578
7.572
7.565
7.558
7.546
3.536
3.416
2.825
2.564
2.552
2.393
2.362
A-1
0.89
1.09
1.17
0.91
2.18
1.21
1.04
3.72
2.35
3.00
f1 (ppm)

Wagdy eldehna -RB4-carbon-Es

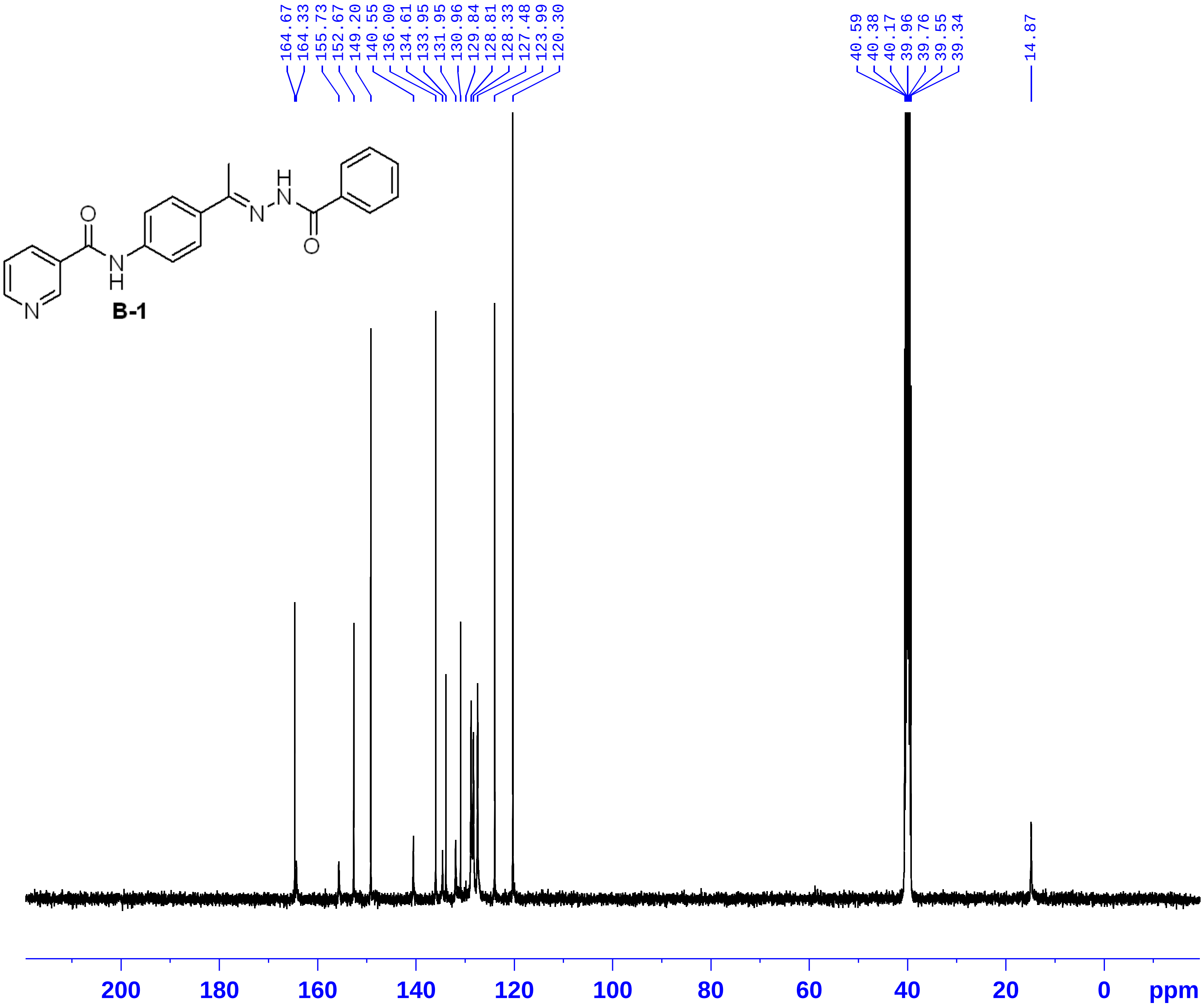


**B-1**

BRUKER

Faculty of Pharmacy

Current Data Parameters

| | |
|---|---|
| NAME | Wagdy eldehna -RB4-carbon-Es |
| EXPNO | 10 |
| PROCNO | 1 |

F2 - Acquisition Parameters

| | |
|---|---|
| Date_ | 20210627 |
| Time | 19.58 h |
| INSTRUM | spect |
| PROBHD | Z108618_0945 ( |
| PULPROG | zgpg30 |
| TD | 65536 |
| SOLVENT | DMSO |
| NS | 2200 |
| DS | 4 |
| SWH | 24038.461 Hz |
| FIDRES | 0.733596 Hz |
| AQ | 1.3631488 sec |
| RG | 197.77 |
| DW | 20.800 usec |
| DE | 6.50 usec |
| TE | 297.5 K |
| D1 | 2.00000000 sec |
| D11 | 0.03000000 sec |
| TD0 | 1 |
| SFO1 | 100.6404331 MHz |
| NUC1 | 13C |
| P1 | 10.00 usec |
| PLW1 | 47.00000000 W |
| SFO2 | 400.2016008 MHz |
| NUC2 | 1H |
| CPDPRG[2 | waltz16 |
| PCPD2 | 90.00 usec |
| PLW2 | 13.00000000 W |
| PLW12 | 0.29249999 W |
| PLW13 | 0.14713000 W |

F2 - Processing parameters

| | |
|---|---|
| SI | 32768 |
| SF | 100.6303700 MHz |
| WDW | EM |
| SSB | 0 |
| LB | 1.00 Hz |
| GB | 0 |
| PC | 1.40 |

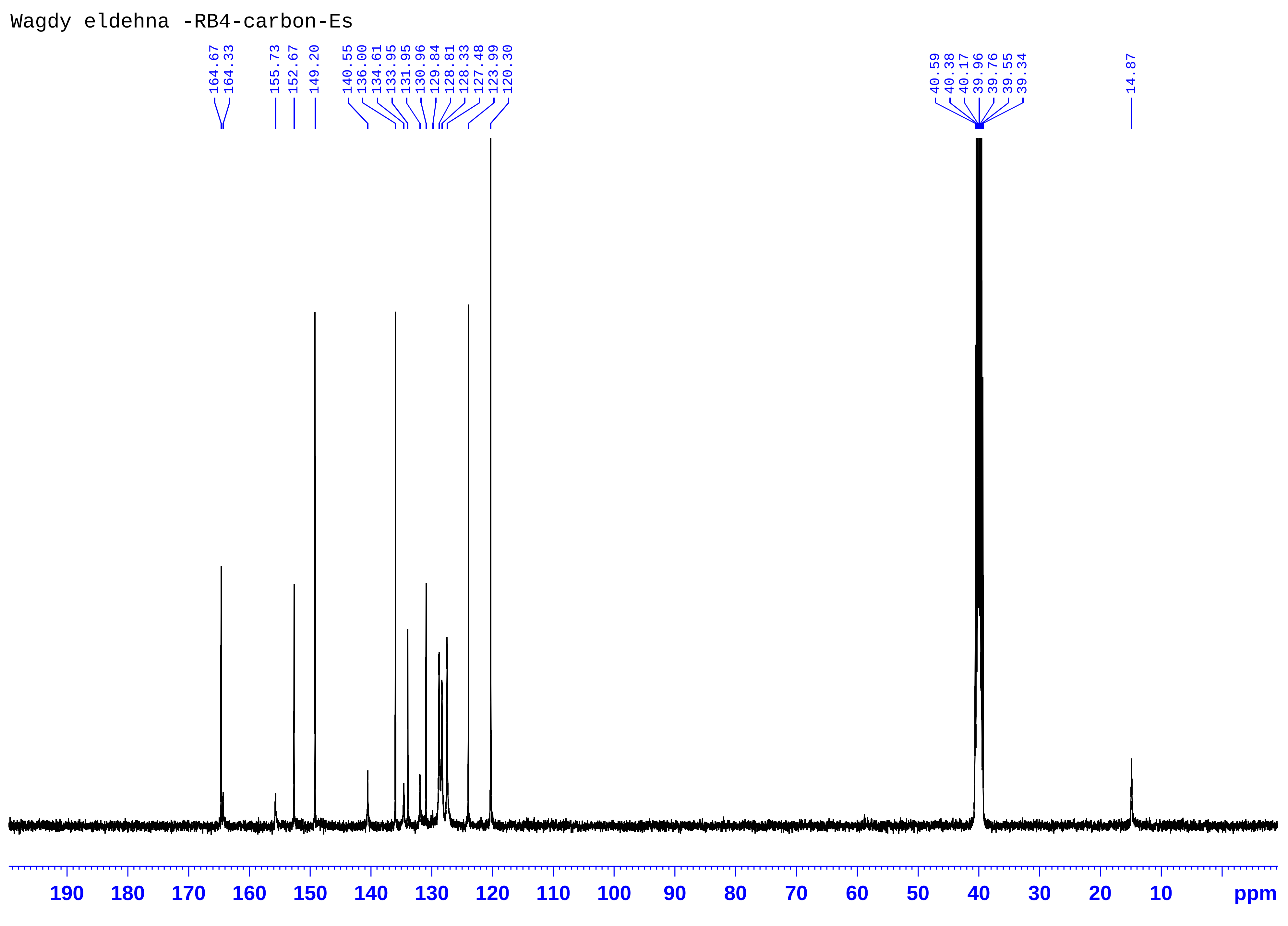
Wagdy eldehna -RB4-carbon-Es
164.67
164.33
155.73
152.67
149.20
140.55
136.00
134.61
133.95
131.95
130.96
129.84
128.81
128.33
127.48
123.99
120.30
40.59
40.38
40.17
39.96
39.76
39.55
39.34
14.87
190
180
170
160
150
140
130
120
110
100
90
80
70
60
50
40
30
20
10
ppm

Wagdy eldehna -RB4-carbon-Es

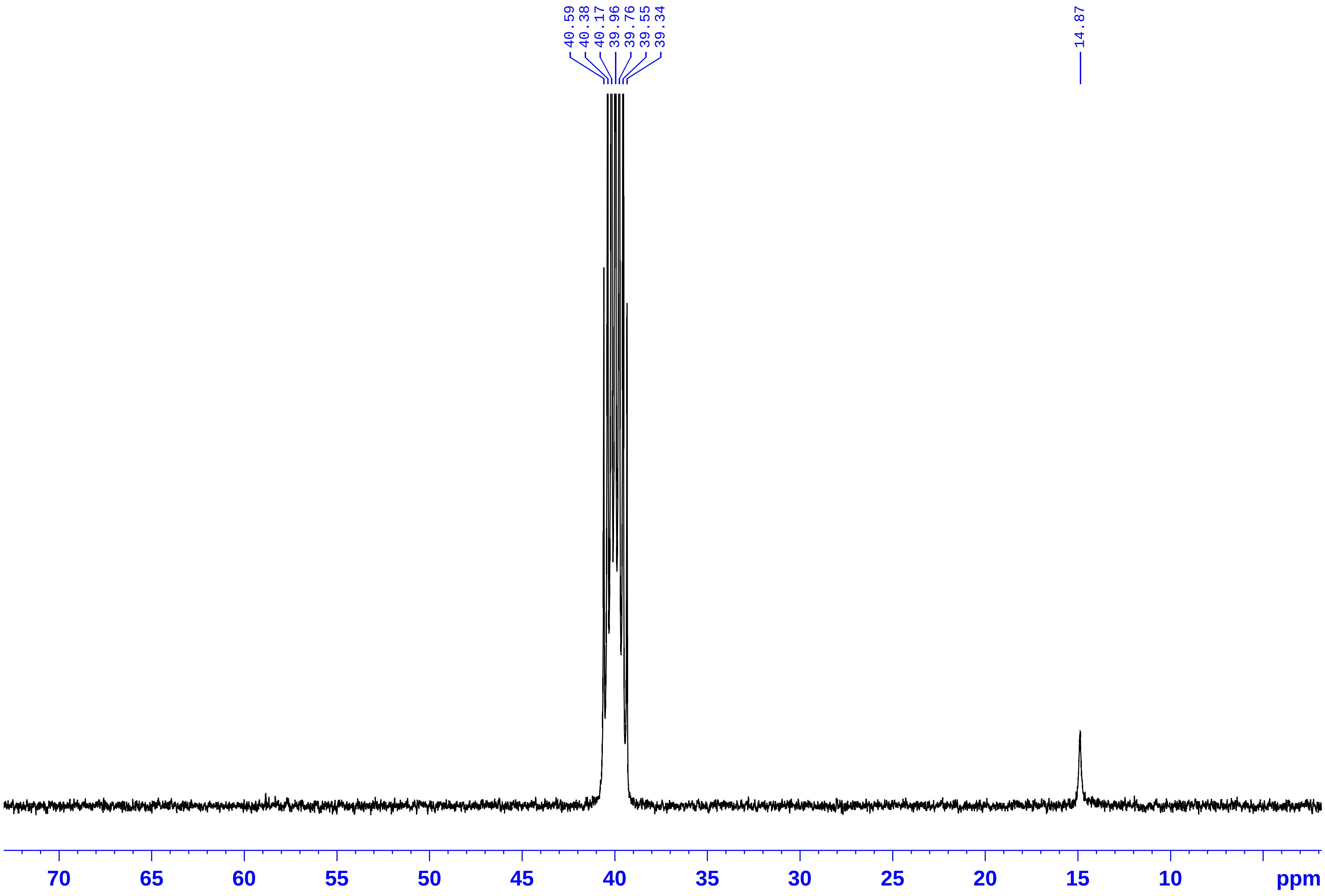

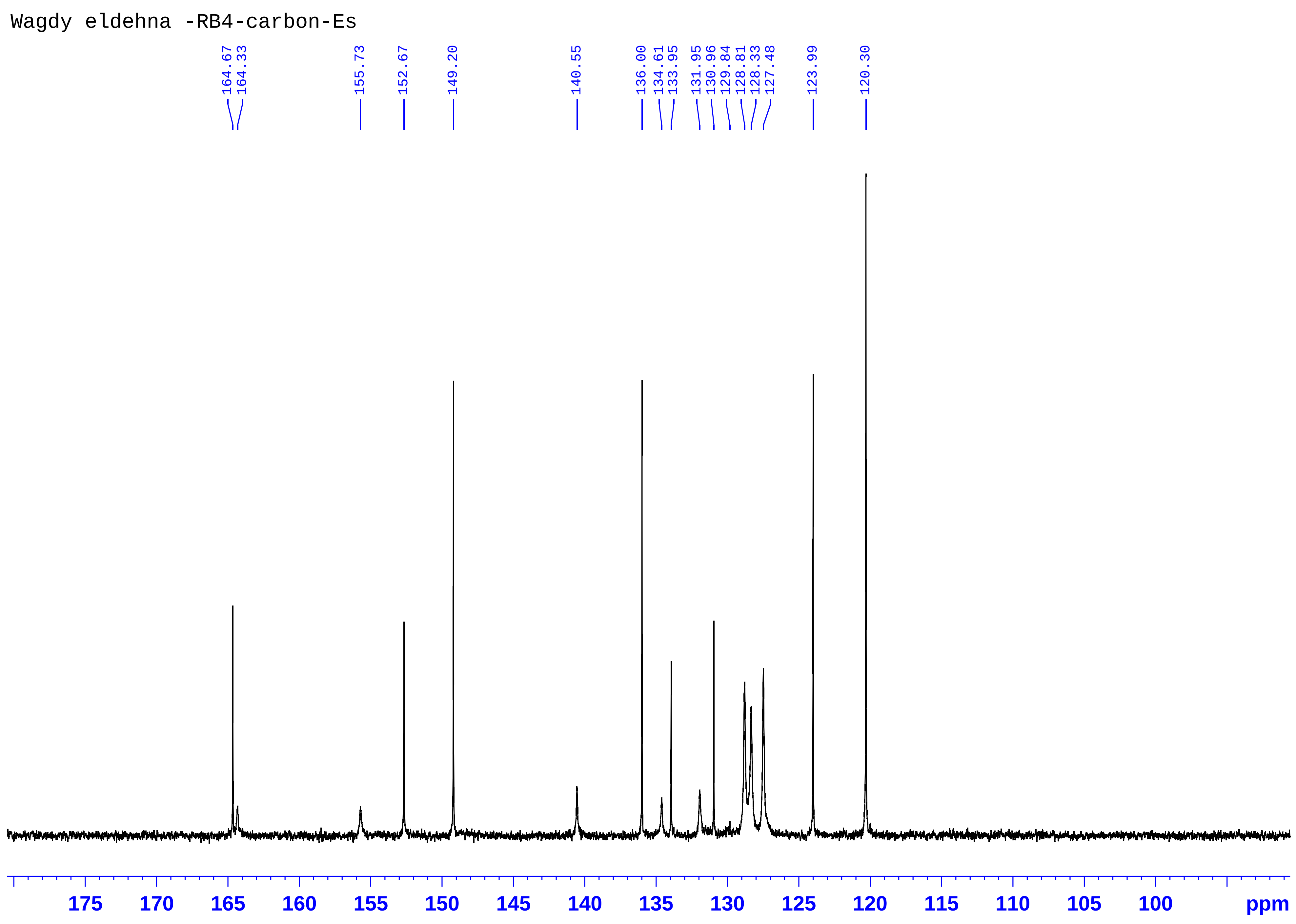
Wagdy eldehna -RB4-carbon-Es
164.67
164.33
155.73
152.67
149.20
140.55
136.00
134.61
133.95
131.95
130.96
129.84
128.81
128.33
127.48
123.99
120.30
175
170
165
160
155
150
145
140
135
130
125
120
115
110
105
100
ppm

Wagdy El Dahna-RB 4-DMSO-Hnmr-A

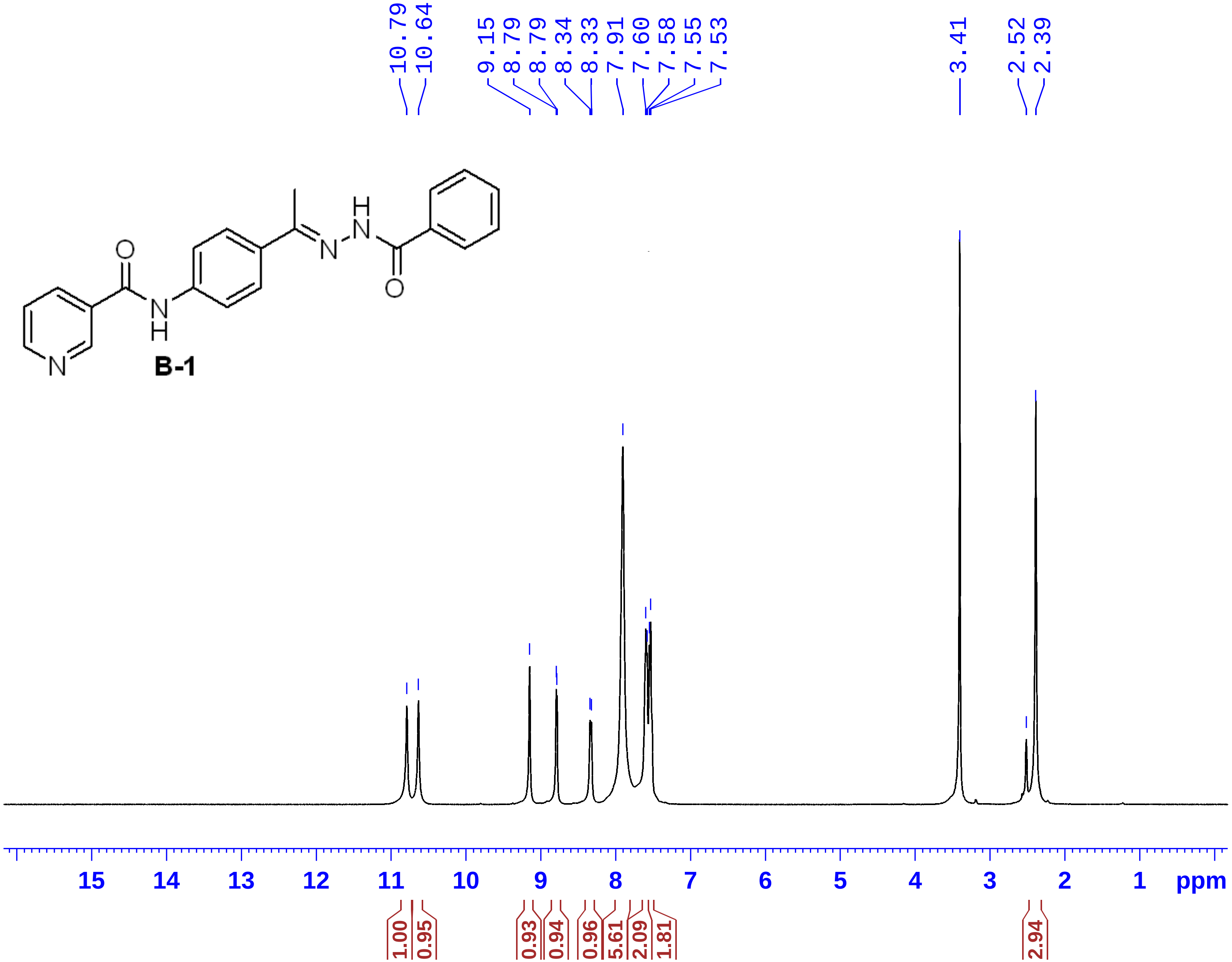


B-1

Current Data Parameters

| | |
|---|---|
| NAME | Wagdy El Dahna-RB |
| EXPNO | 10 |
| PROCNO | 1 |

F2 - Acquisition Parameter

| | |
|---|---|
| Date_ | 20210610 |
| Time | 11.19 h |
| INSTRUM | spect |
| PROBHD | Z108618_0945 ( |
| PULPROG | zg30 |
| TD | 65536 |
| SOLVENT | DMSO |
| NS | 16 |
| DS | 2 |
| SWH | 8012.820 Hz |
| FIDRES | 0.244532 Hz |
| AQ | 4.0894465 se |
| RG | 88.92 |
| DW | 62.400 us |
| DE | 6.50 us |
| TE | 294.5 K |
| D1 | 1.00000000 se |
| TD0 | 1 |
| SFO1 | 400.2024712 MH |
| NUC1 | 1H |
| P1 | 13.50 us |
| PLW1 | 13.00000000 W |

F2 - Processing parameters

| | |
|---|---|
| SI | 65536 |
| SF | 400.2000000 MH |
| WDW | EM |
| SSB | 0 |
| LB | 0.30 Hz |
| GB | 0 |
| PC | 1.00 |

Wagdy El Dahna-RB 4-DMSO-Hnmr-A

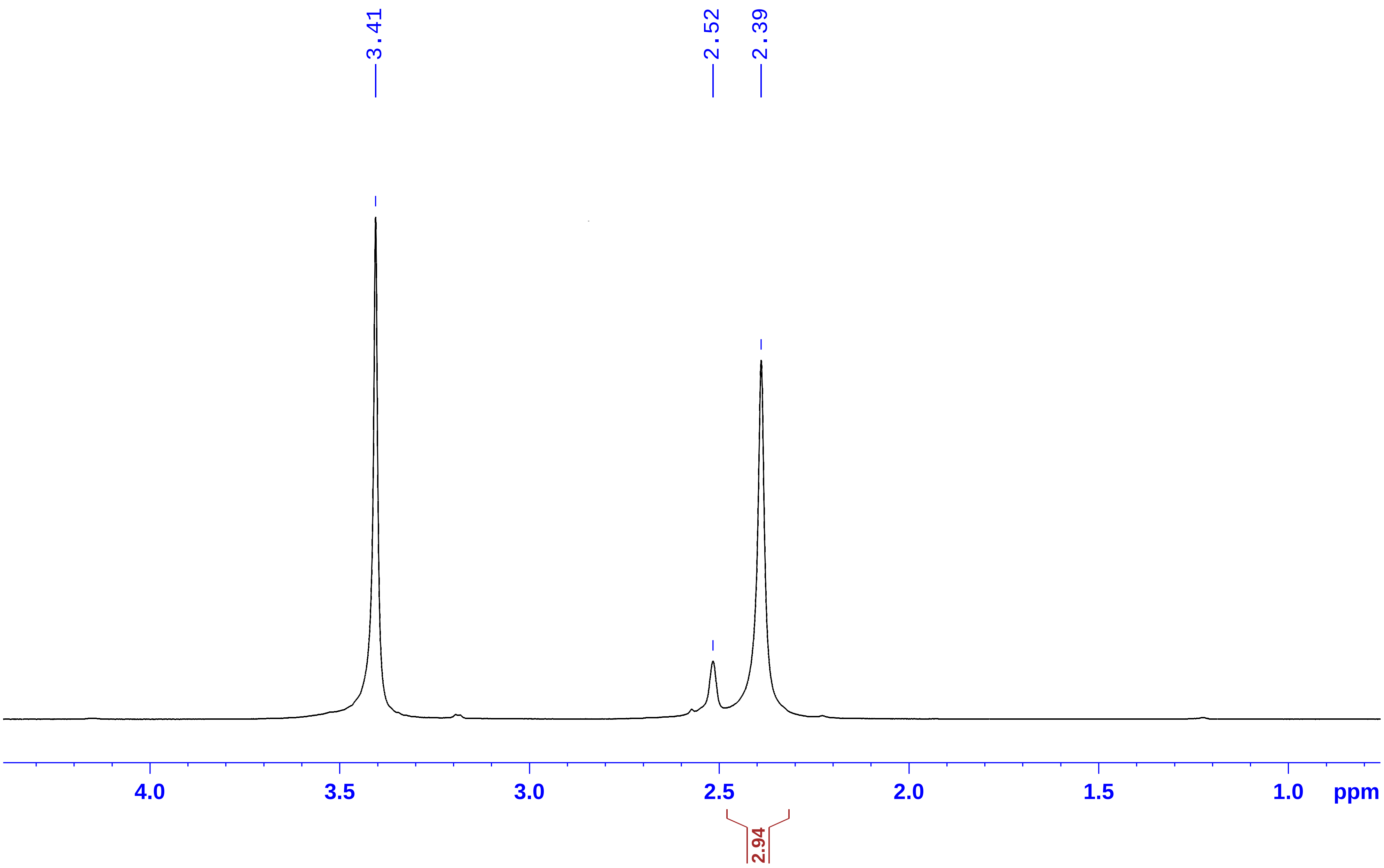

Wagdy El Dahna-RB 4-DMSO-Hnmr-A

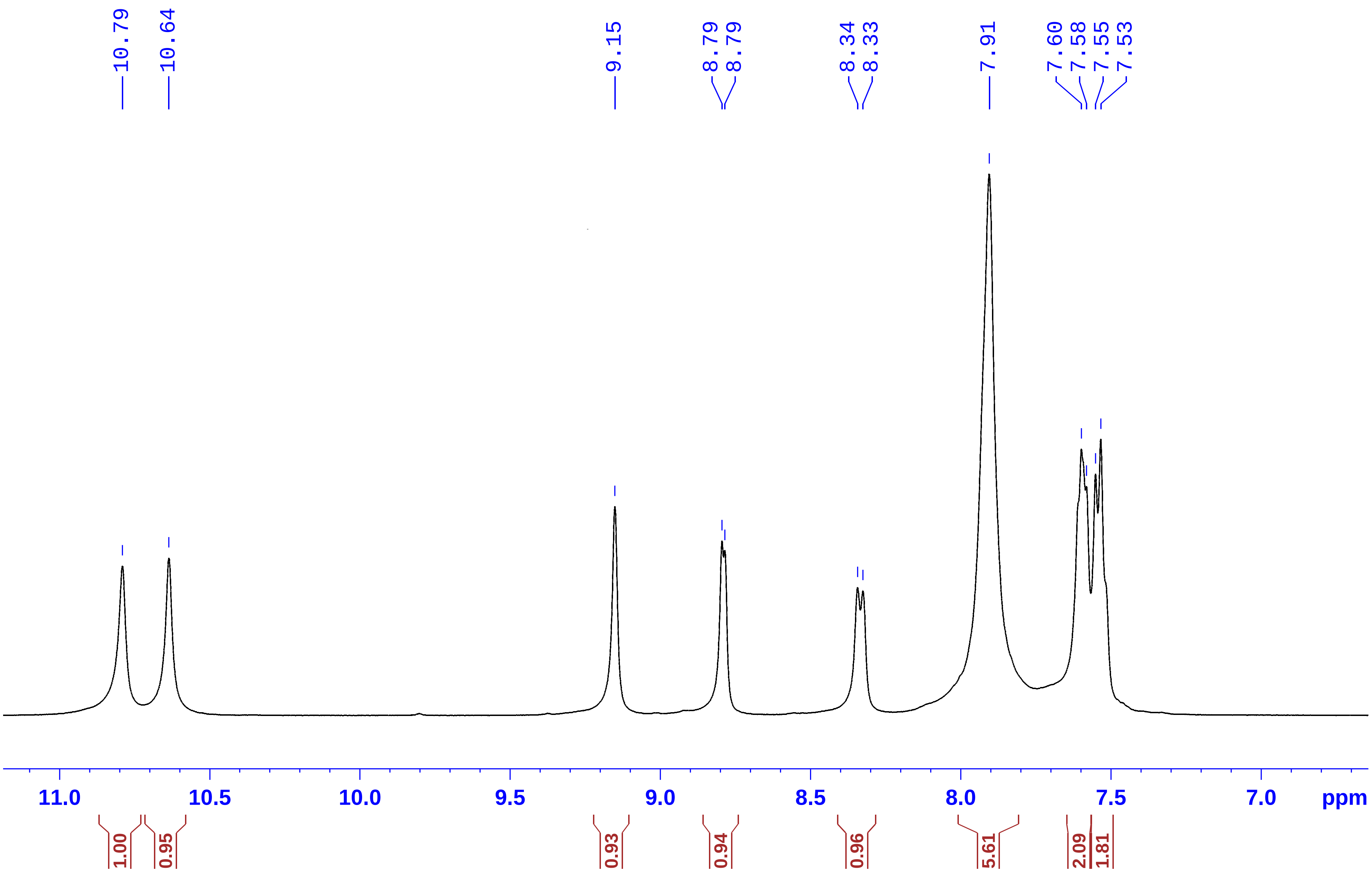

Wagdy eldehna -RB5-carbon-Es

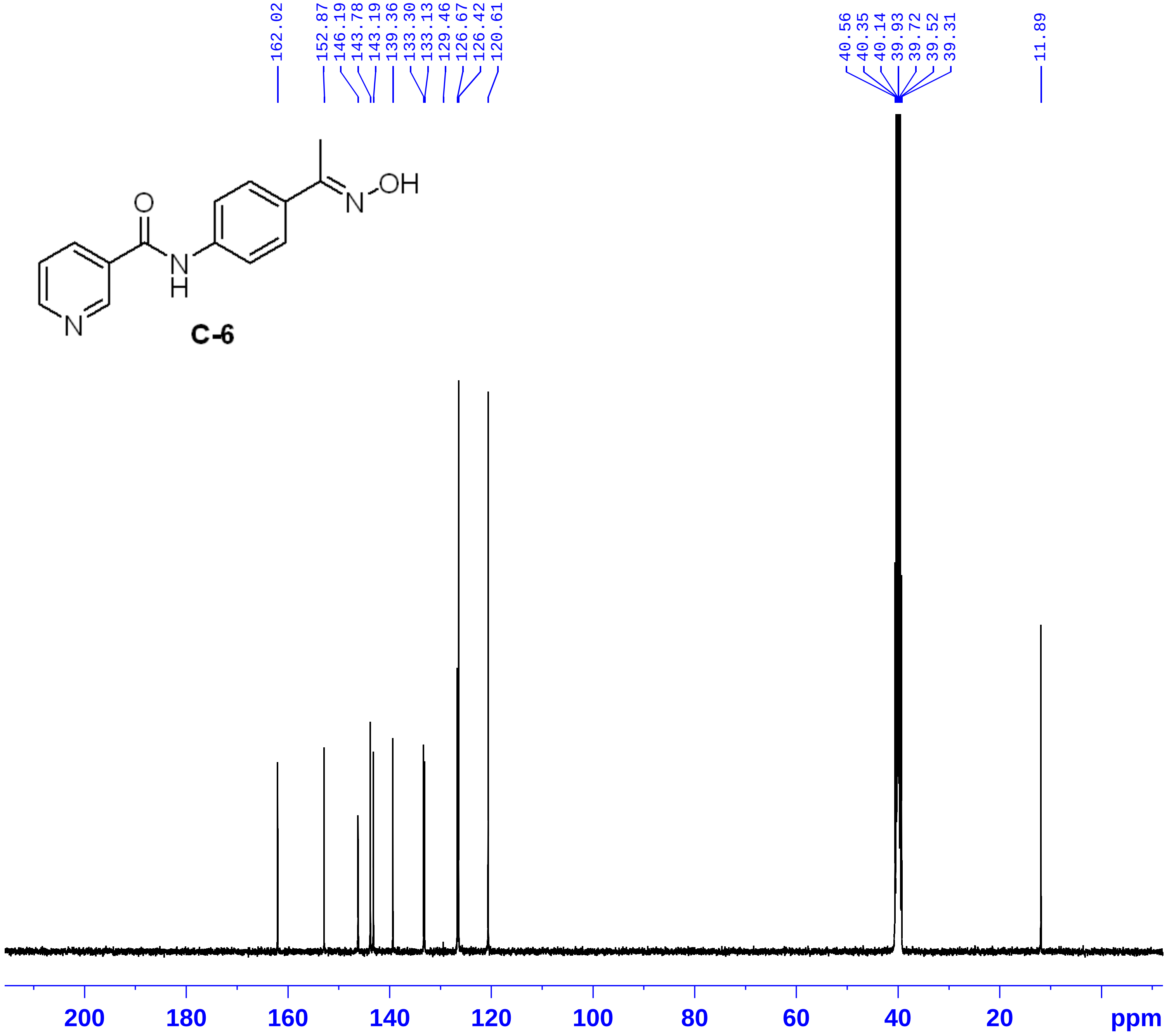


**C-6**

BRUKER

Faculty of Pharmacy

Current Data Parameters

| | |
|---|---|
| NAME | Wagdy eldehna -RB5-carbon-Es |
| EXPNO | 10 |
| PROCNO | 1 |

F2 - Acquisition Parameters

| | |
|---|---|
| Date_ | 20210628 |
| Time | 6.44 h |
| INSTRUM | spect |
| PROBHD | Z108618_0945 ( |
| PULPROG | zgpg30 |
| TD | 65536 |
| SOLVENT | DMSO |
| NS | 2200 |
| DS | 4 |
| SWH | 24038.461 Hz |
| FIDRES | 0.733596 Hz |
| AQ | 1.3631488 sec |
| RG | 197.77 |
| DW | 20.800 usec |
| DE | 6.50 usec |
| TE | 297.4 K |
| D1 | 2.00000000 sec |
| D11 | 0.03000000 sec |
| TD0 | 1 |
| SFO1 | 100.6404331 MHz |
| NUC1 | 13C |
| P1 | 10.00 usec |
| PLW1 | 47.00000000 W |
| SFO2 | 400.2016008 MHz |
| NUC2 | 1H |
| CPDPRG[2 | waltz16 |
| PCPD2 | 90.00 usec |
| PLW2 | 13.00000000 W |
| PLW12 | 0.29249999 W |
| PLW13 | 0.14713000 W |

F2 - Processing parameters

| | |
|---|---|
| SI | 32768 |
| SF | 100.6303700 MHz |
| WDW | EM |
| SSB | 0 |
| LB | 1.00 Hz |
| GB | 0 |
| PC | 1.40 |

Wagdy eldehna -RB5-carbon-Es

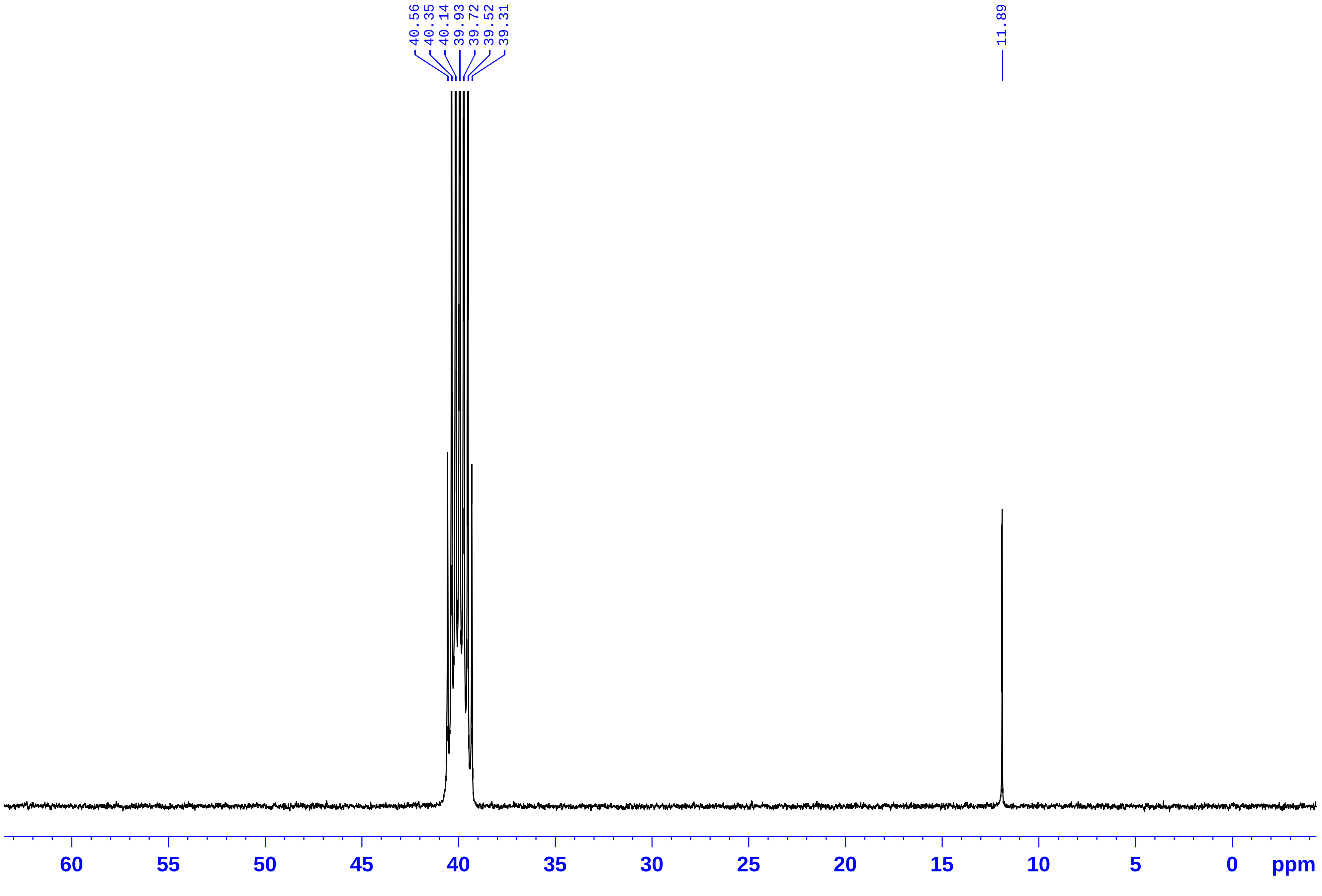

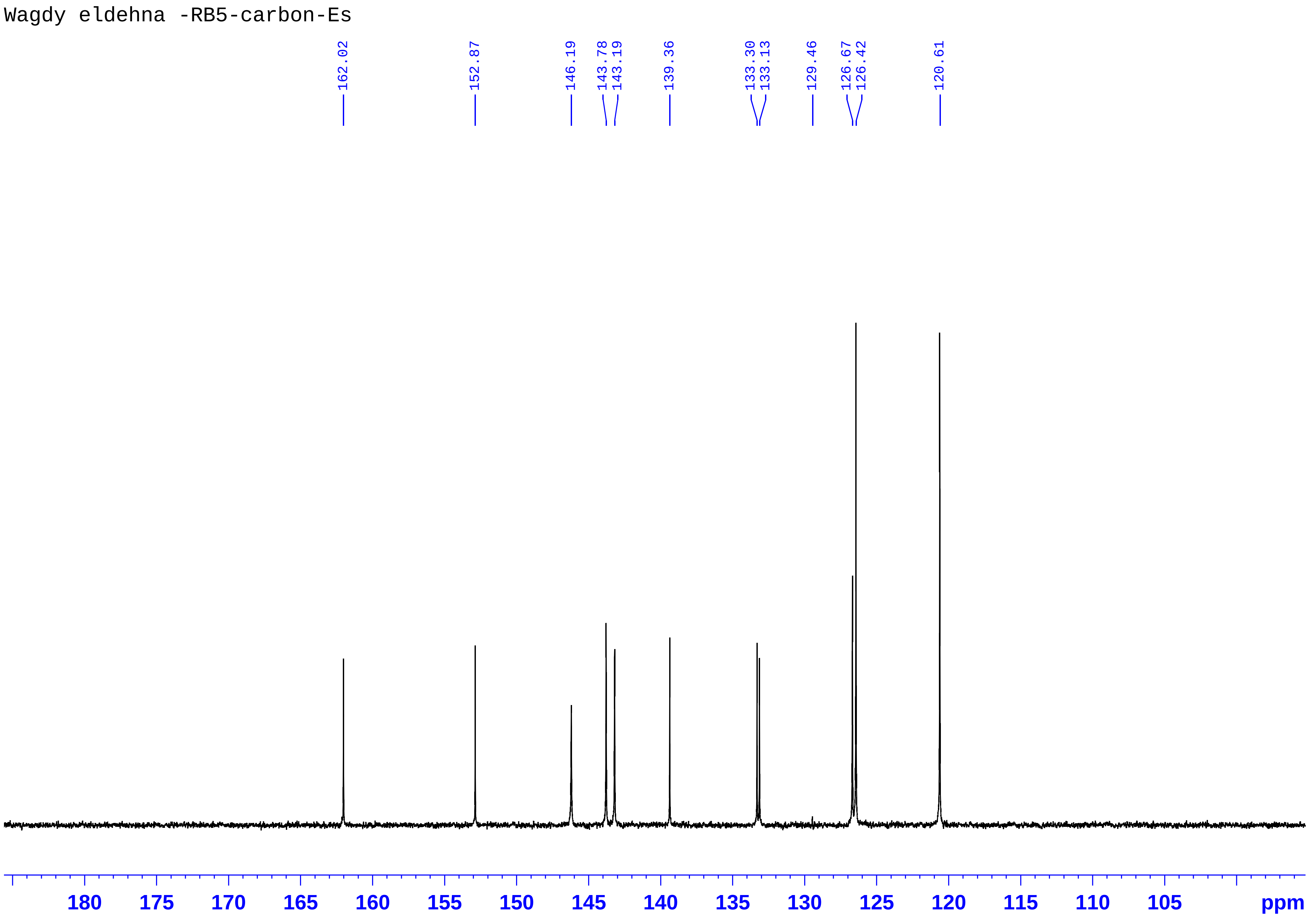
Wagdy eldehna -RB5-carbon-Es
162.02
152.87
146.19
143.78
143.19
139.36
133.30
133.13
129.46
126.67
126.42
120.61
180
175
170
165
160
155
150
145
140
135
130
125
120
115
110
105
ppm

Wagdy El Dahna-RB 5-DMSO-Hnmr-A

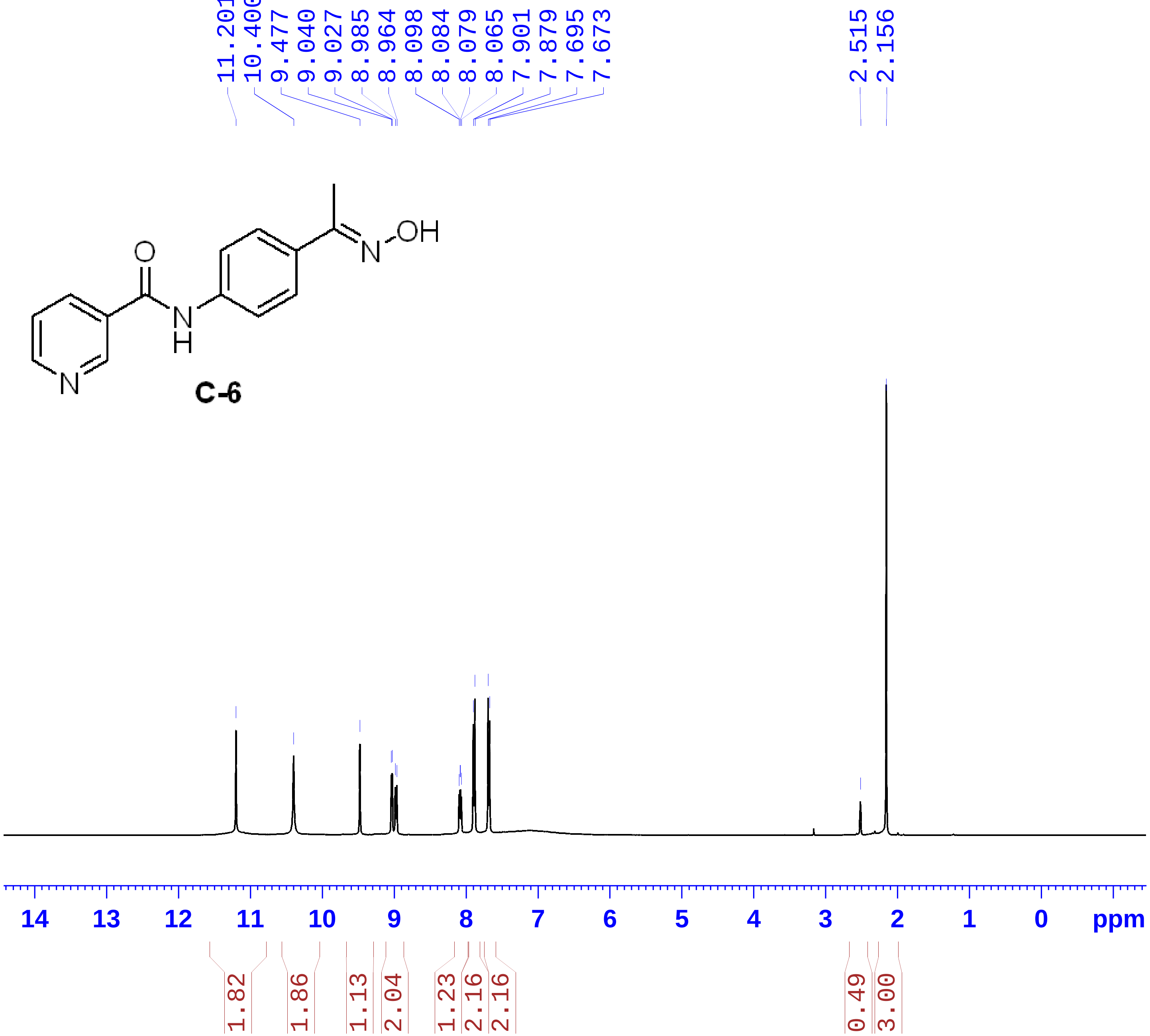


C-6

Faculty of Pharmacy

Current Data Parameters
NAME Wagdy El Dahna-RB 5-DMSO-H
EXPNO 10
PROCNO 1

F2 - Acquisition Parameters
Date_ 20210610
Time 11.43 h
INSTRUM spect
PROBHD Z108618_0945 (
PULPROG zg30
TD 65536
SOLVENT DMSO
NS 16
DS 2
SWH 8012.820 Hz
FIDRES 0.244532 Hz
AQ 4.0894465 sec
RG 99.3
DW 62.400 usec
DE 6.50 usec
TE 294.3 K
D1 1.00000000 sec
TD0 1
SFO1 400.2024712 MHz
NUC1 1H
P1 13.50 usec
PLW1 13.00000000 W

F2 - Processing parameters
SI 65536
SF 400.2000000 MHz
WDW EM
SSB 0
LB 0.30 Hz
GB 0
PC 1.00

Wagdy El Dahna-RB 5-DMSO-Hnmr-A

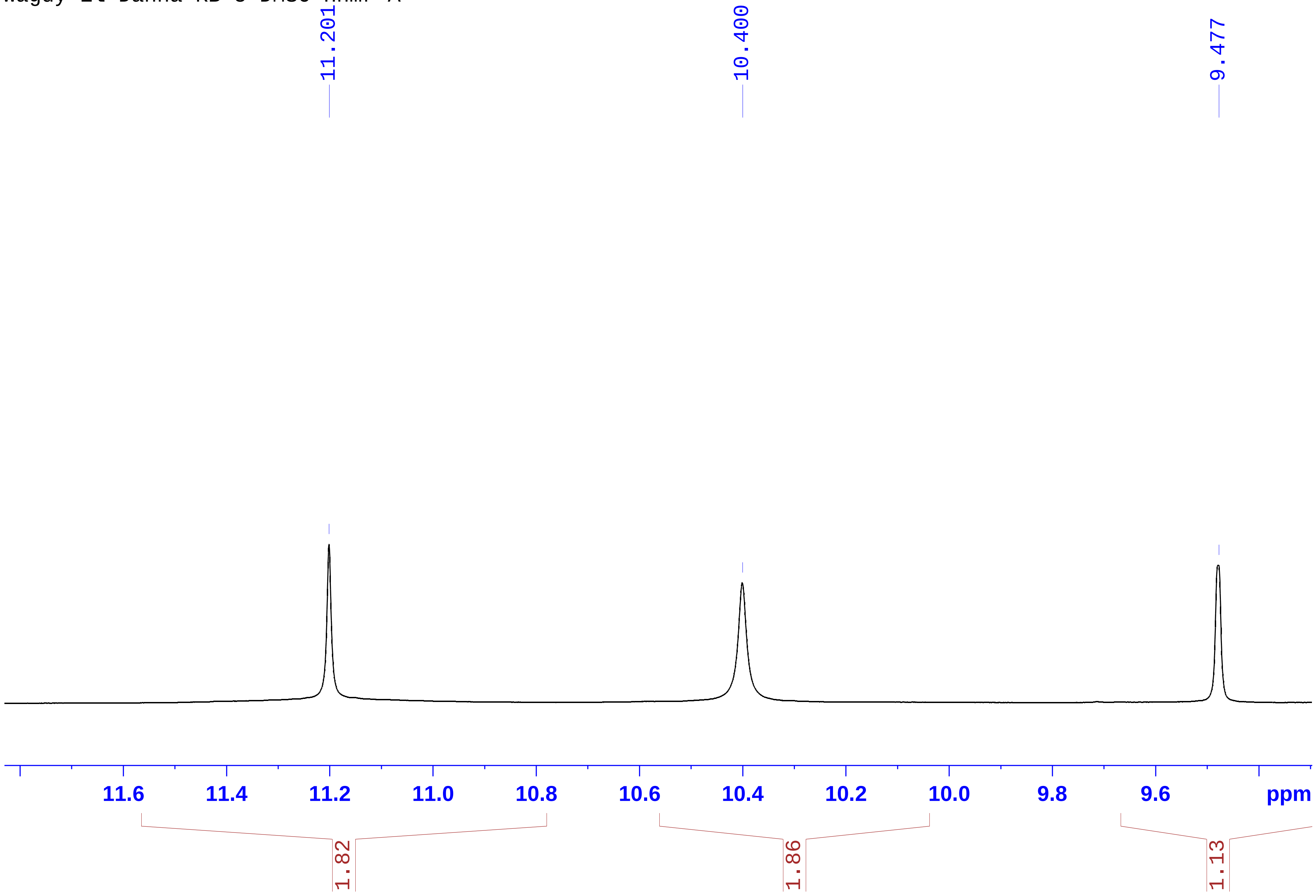

Wagdy El Dahna-RB 5-DMSO-Hnmr-A

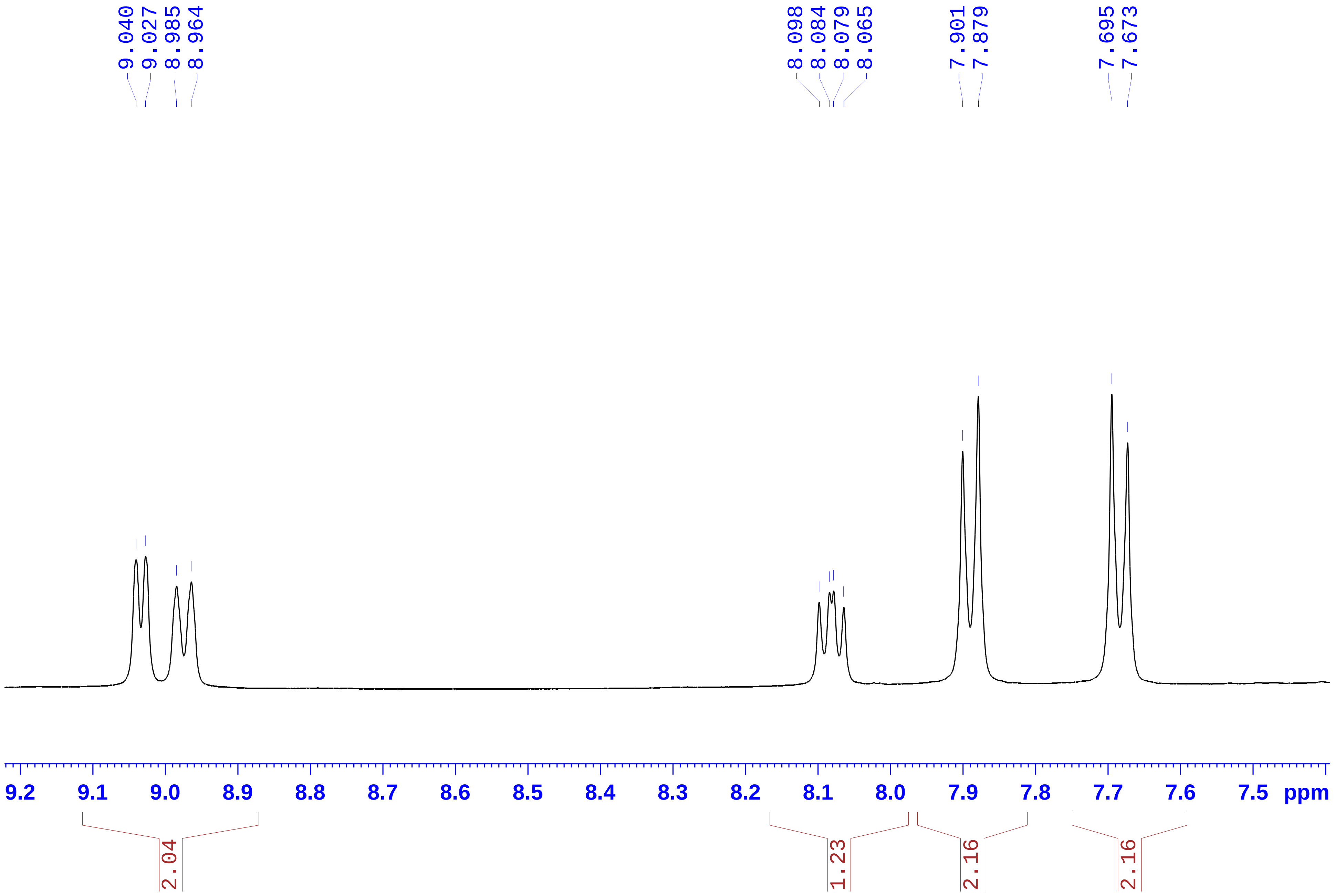

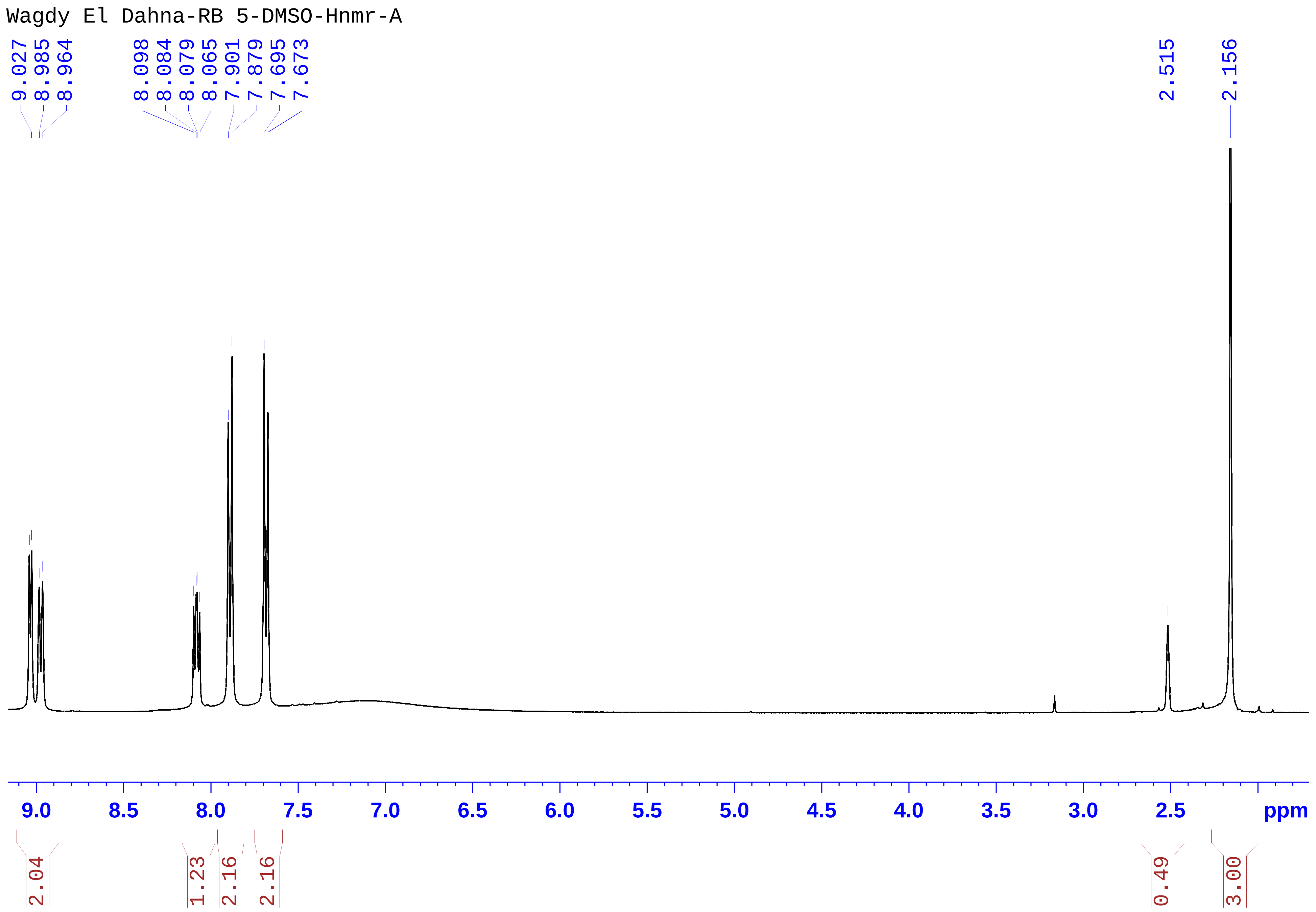
Wagdy El Dahna-RB 5-DMSO-Hnmr-A
9.027
8.985
8.964
8.098
8.084
8.079
8.065
7.901
7.879
7.695
7.673
2.515
2.156
9.0
8.5
8.0
7.5
7.0
6.5
6.0
5.5
5.0
4.5
4.0
3.5
3.0
2.5
ppm
2.04
1.23
2.16
2.16
0.49
3.00

Wagdy eldehna -RB8-carbon-Es

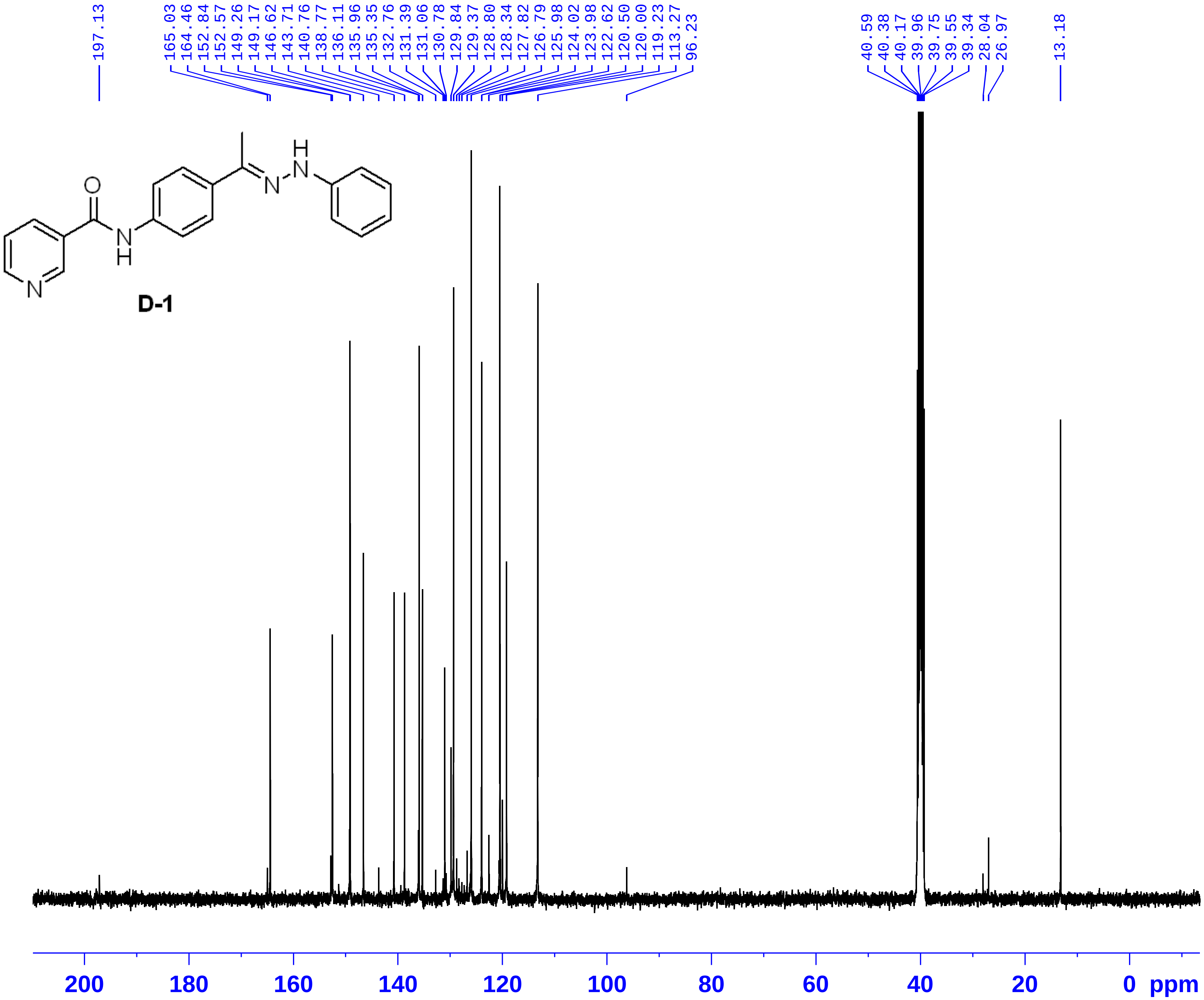


Current Data Parameters
NAME Wagdy eldehna -RB8-carbon-Es
EXPNO 10
PROCNO 1

F2 - Acquisition Parameters
Date_ 20210627
Time 22.07 h
INSTRUM spect
PROBHD Z108618_0945 (
PULPROG zgpg30
TD 65536
SOLVENT DMSO
NS 2200
DS 4
SWH 24038.461 Hz
FIDRES 0.733596 Hz
AQ 1.3631488 sec
RG 197.77
DW 20.800 usec
DE 6.50 usec
TE 297.3 K
D1 2.00000000 sec
D11 0.03000000 sec
TD0 1
SFO1 100.6404331 MHz
NUC1 13C
P1 10.00 usec
PLW1 47.00000000 W
SFO2 400.2016008 MHz
NUC2 1H
CPDPRG[2 waltz16
PCPD2 90.00 usec
PLW2 13.00000000 W
PLW12 0.29249999 W
PLW13 0.14713000 W

F2 - Processing parameters
SI 32768
SF 100.6303700 MHz
WDW EM
SSB 0
LB 1.00 Hz
GB 0
PC 1.40

Wagdy eldehna -RB8-carbon-Es

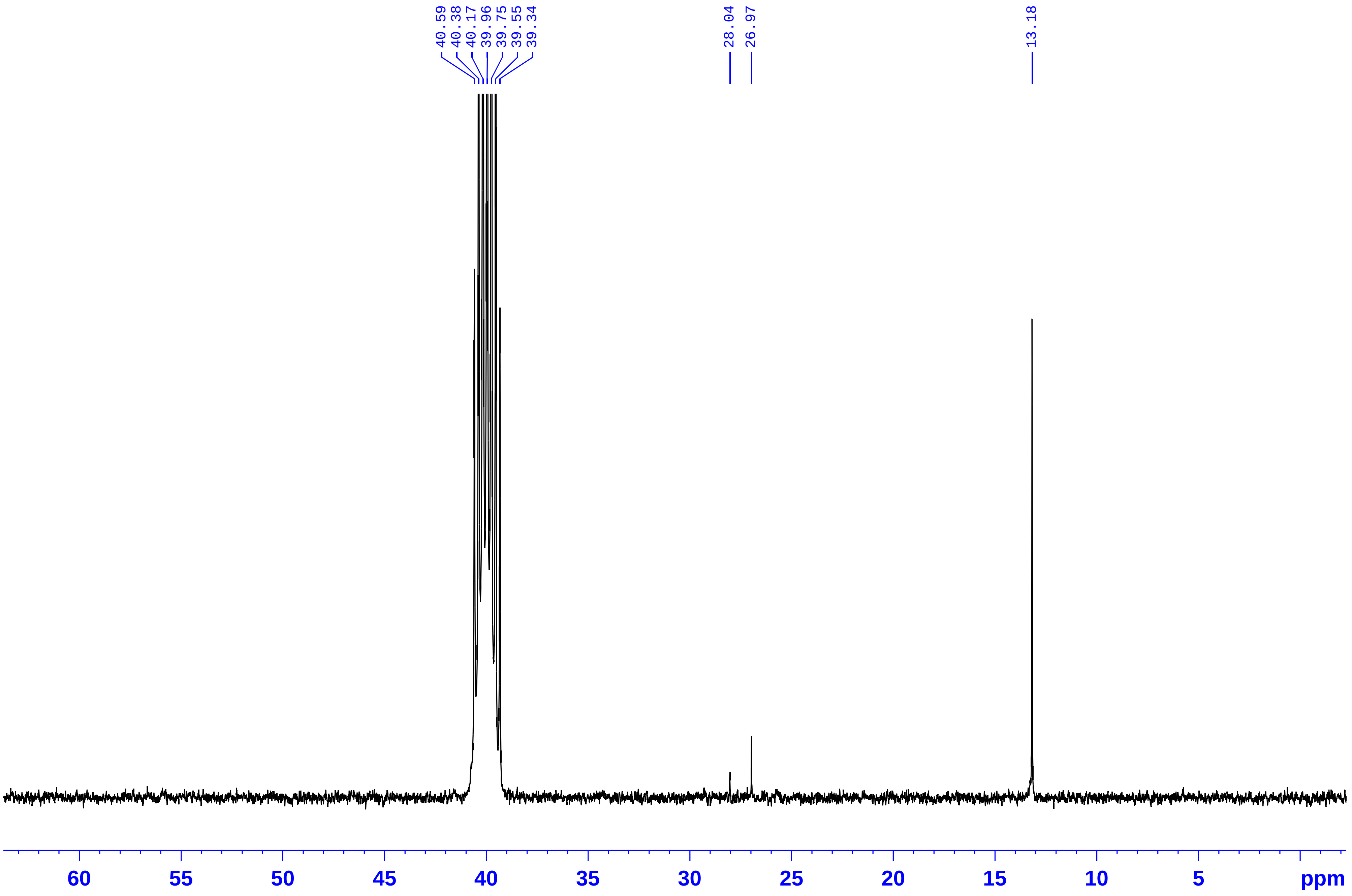

Wagdy eldehna -RB8-carbon-Es

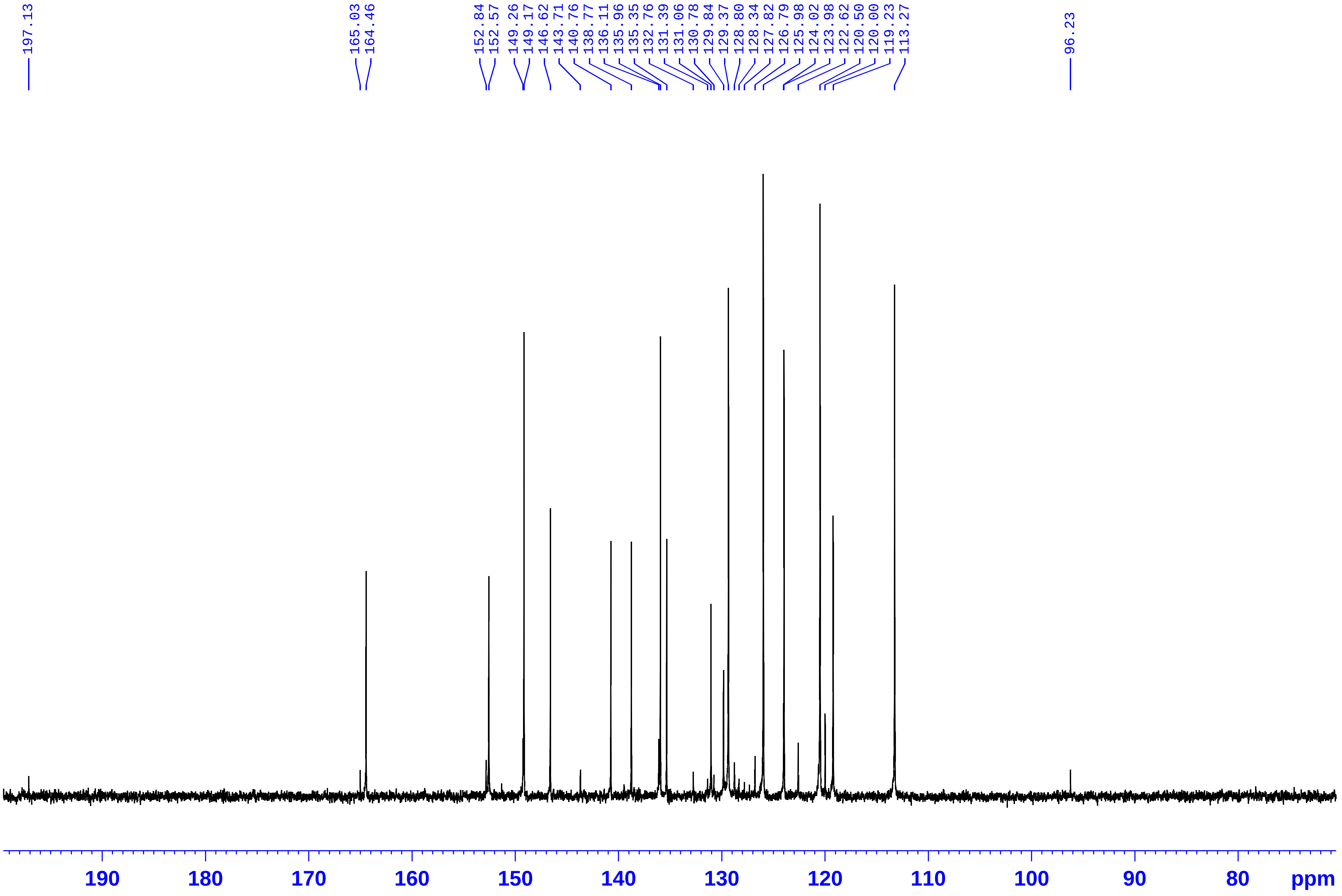

Wagdy El Dahna-RB 8-DMSO-Hnmr-A

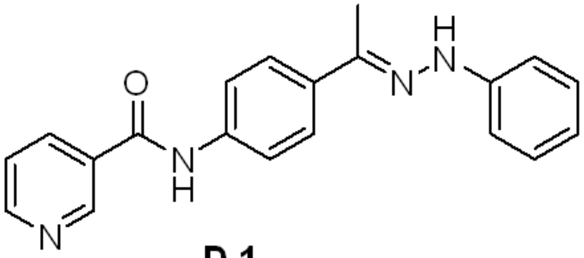

**D-1**

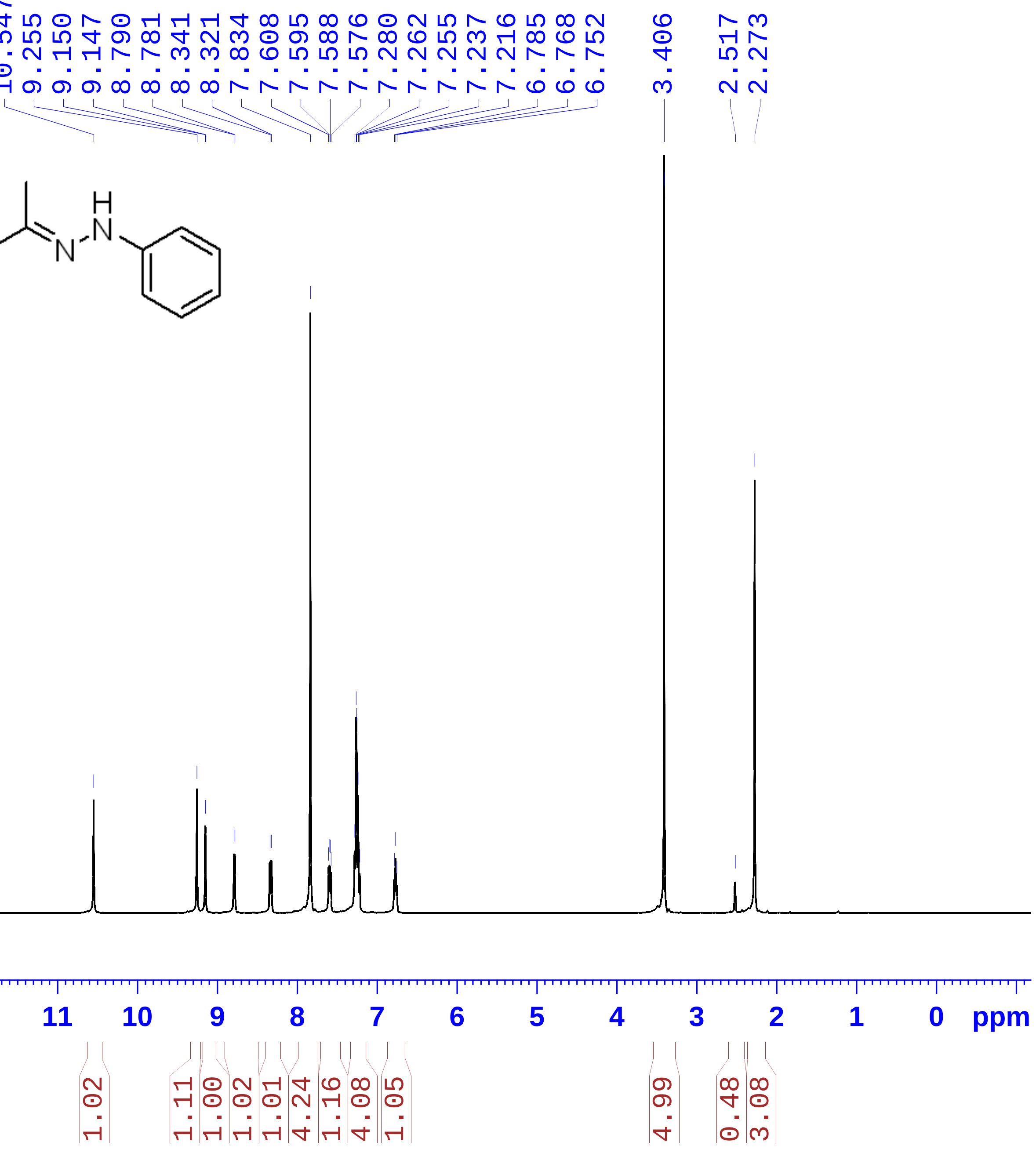


Current Data Parameters

| | |
|---|---|
| NAME | Wagdy El Dahna-RB 8-DMSO-H |
| EXPNO | 10 |
| PROCNO | 1 |

F2 - Acquisition Parameters

| | |
|---|---|
| Date_ | 20210610 |
| Time | 11.24 h |
| INSTRUM | spect |
| PROBHD | Z108618_0945 ( |
| PULPROG | zg30 |
| TD | 65536 |
| SOLVENT | DMSO |
| NS | 16 |
| DS | 2 |
| SWH | 8012.820 Hz |
| FIDRES | 0.244532 Hz |
| AQ | 4.0894465 sec |
| RG | 99.3 |
| DW | 62.400 usec |
| DE | 6.50 usec |
| TE | 294.4 K |
| D1 | 1.00000000 sec |
| TD0 | 1 |
| SFO1 | 400.2024712 MHz |
| NUC1 | 1H |
| P1 | 13.50 usec |
| PLW1 | 13.00000000 W |

F2 - Processing parameters

| | |
|---|---|
| SI | 65536 |
| SF | 400.2000000 MHz |
| WDW | EM |
| SSB | 0 |
| LB | 0.30 Hz |
| GB | 0 |
| PC | 1.00 |

Wagdy El Dahna-RB 8-DMSO-Hnmr-A

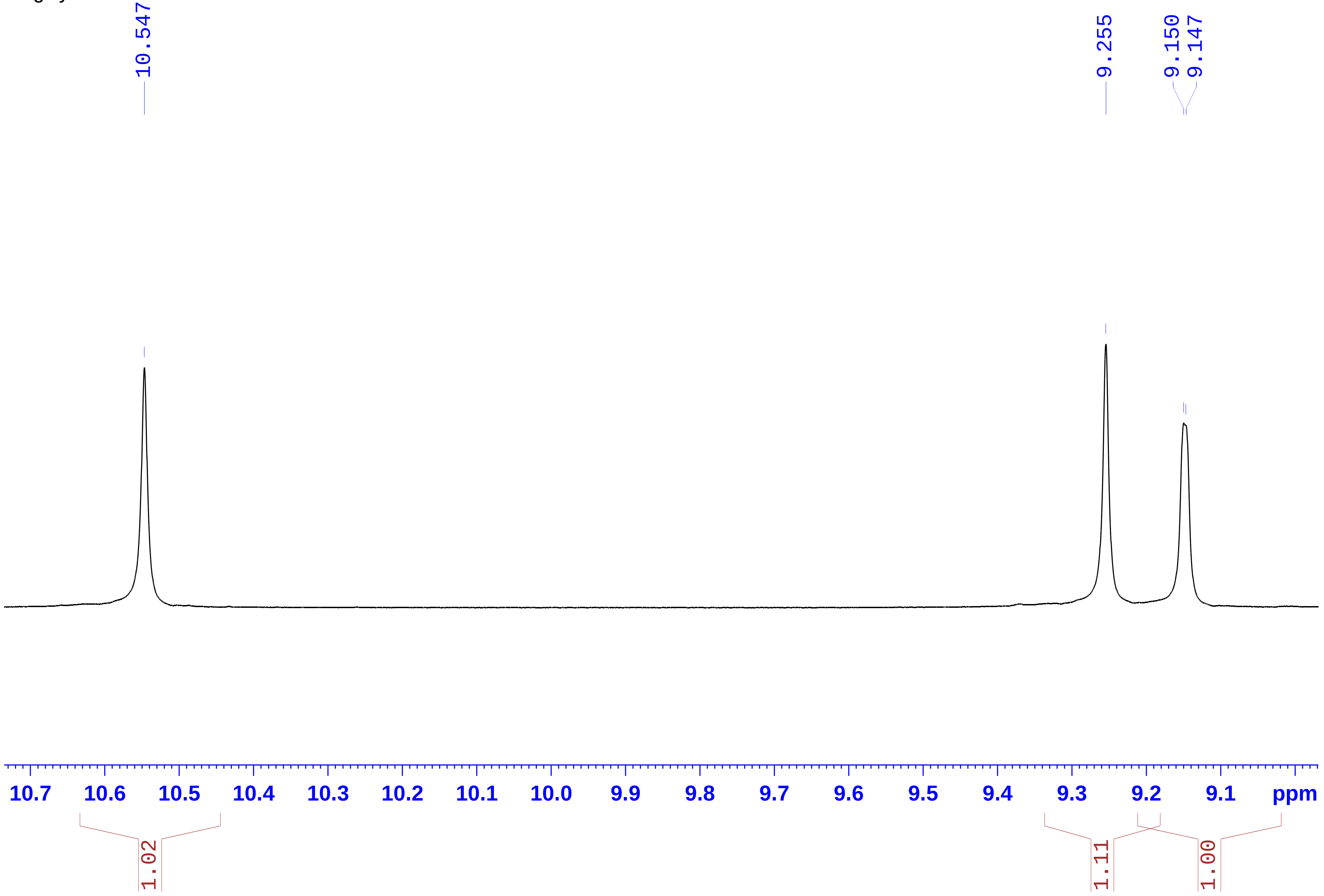

Wagdy El Dahna-RB 8-DMSO-Hnmr-A

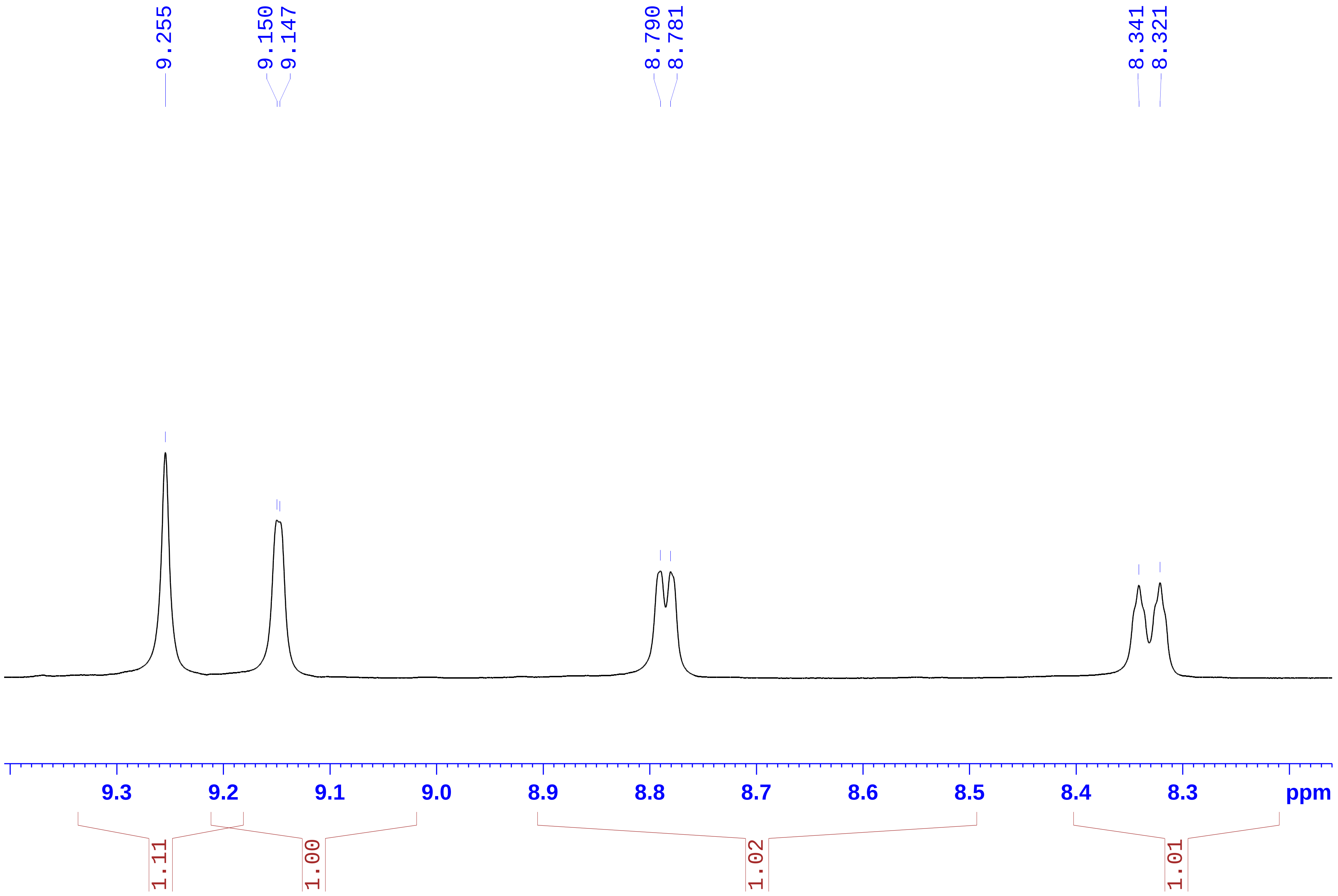

Wagdy El Dahna-RB 8-DMSO-Hnmr-A

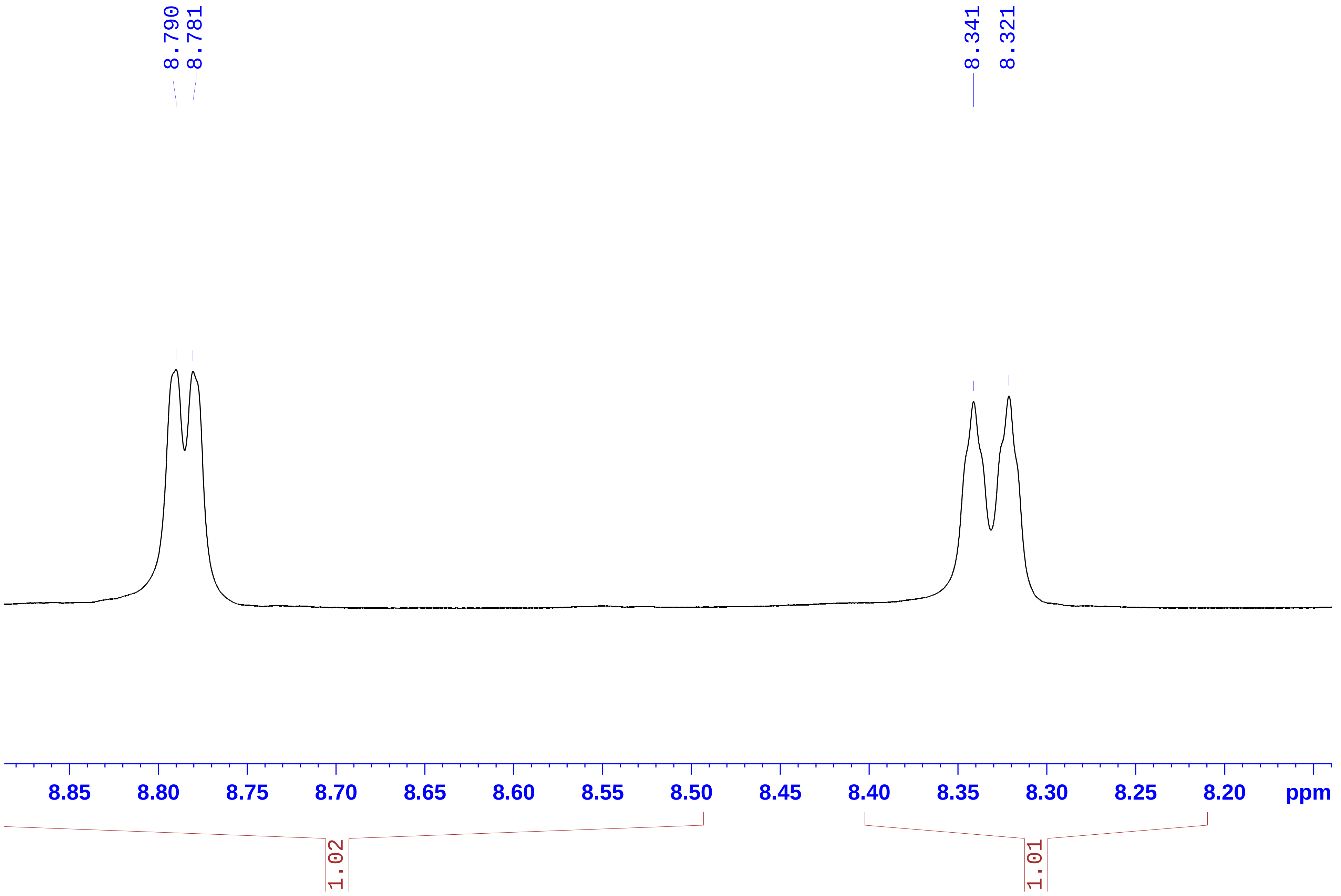

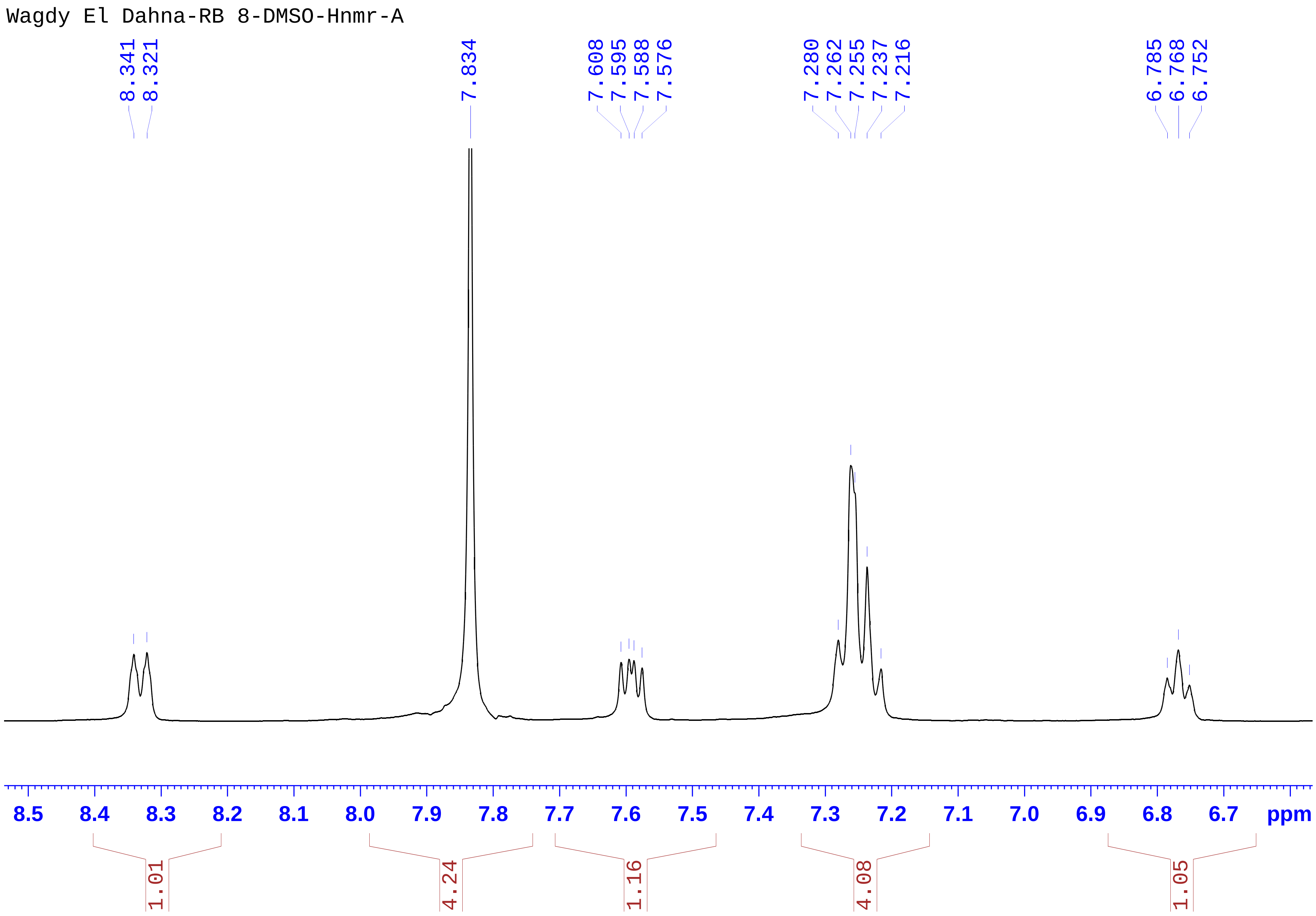

Wagdy El Dahna-RB 8-DMSO-Hnmr-A
8.341
8.321
7.834
7.608
7.595
7.588
7.576
7.280
7.262
7.255
7.237
7.216
6.785
6.768
6.752
8.5 8.4 8.3 8.2 8.1 8.0 7.9 7.8 7.7 7.6 7.5 7.4 7.3 7.2 7.1 7.0 6.9 6.8 6.7 ppm
1.01
4.24
1.16
4.08
1.05

Wagdy El Dahna-RB 8-DMSO-Hnmr-A

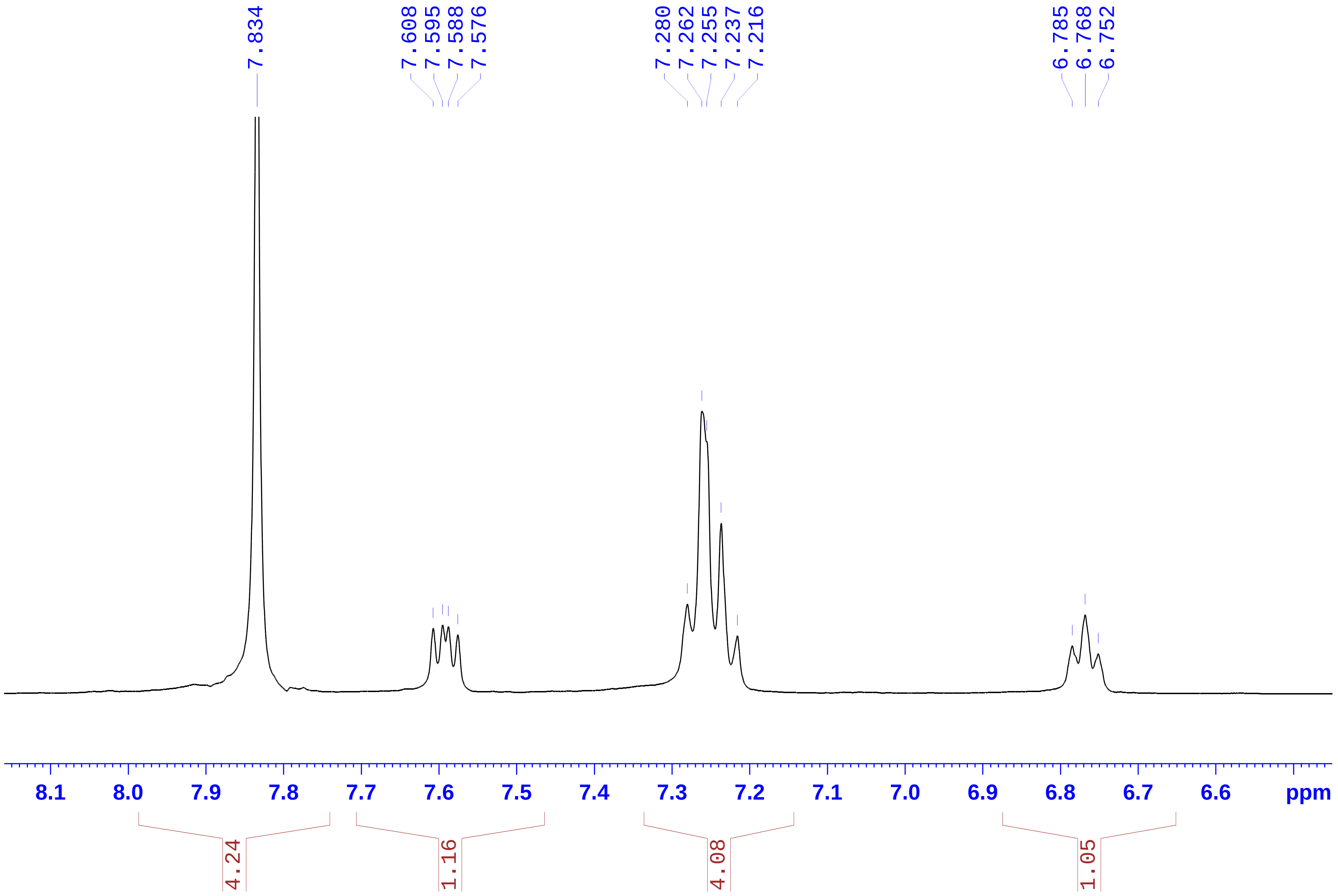

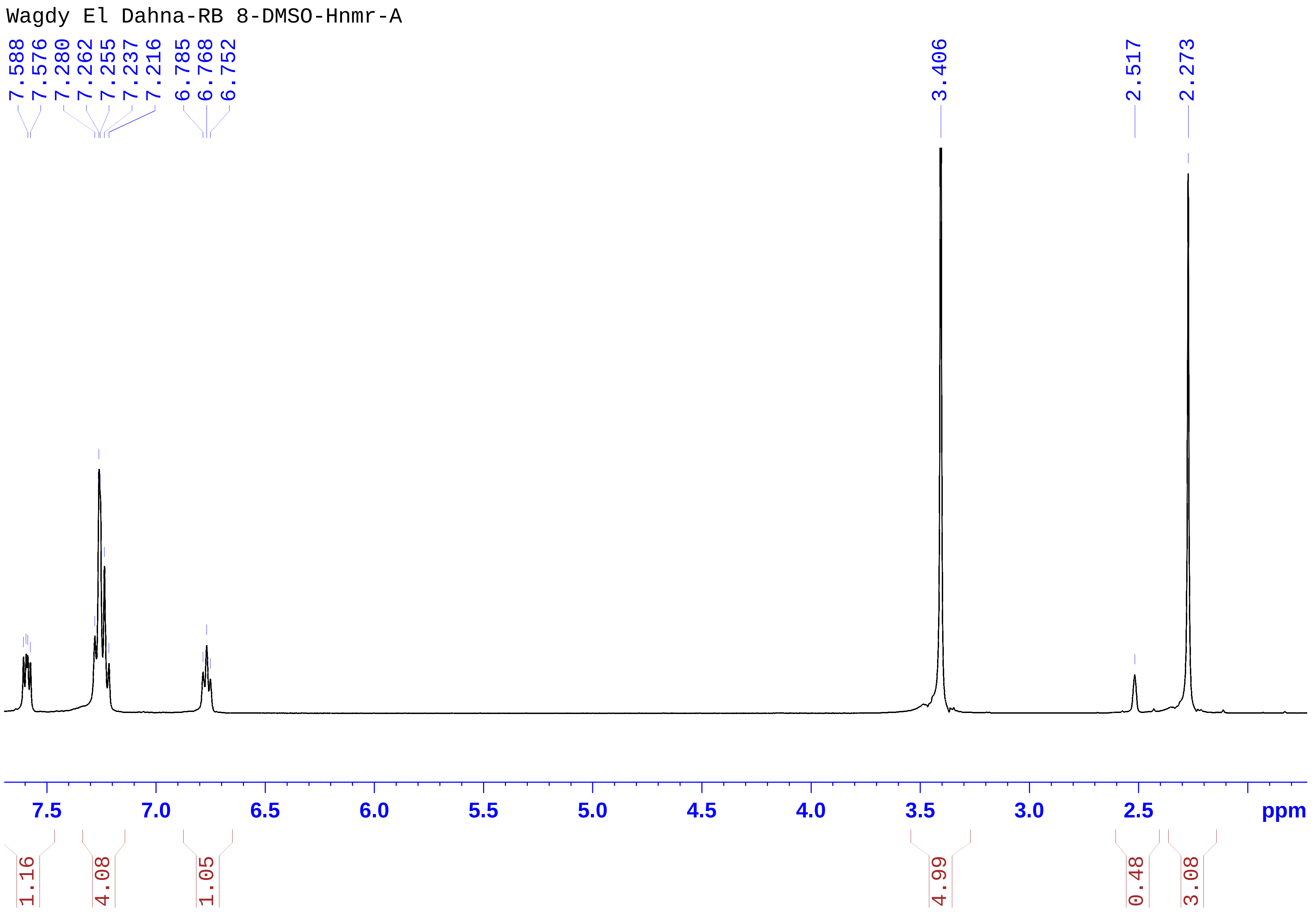
Wagdy El Dahna-RB 8-DMSO-Hnmr-A
7.588
7.576
7.280
7.262
7.255
7.237
7.216
6.785
6.768
6.752
3.406
2.517
2.273
7.5
7.0
6.5
6.0
5.5
5.0
4.5
4.0
3.5
3.0
2.5
ppm
1.16
4.08
1.05
4.99
0.48
3.08

## A-1 TOPKAT_Ames_Mutagenicity

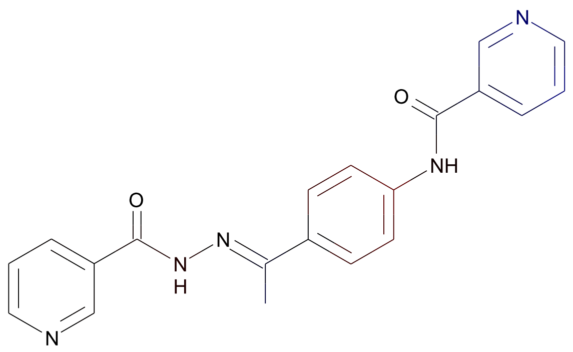


$C_{20}H_{17}N_5O_2$

Molecular Weight: 359.38128

ALogP: 1.147

Rotatable Bonds: 5

Acceptors: 5

Donors: 2

## Structural Similar Compounds

| Name | 77-46-3 | 79455-30-4 | PENICILLIN G POTASSIUM |
|---|---|---|---|
| Structure | 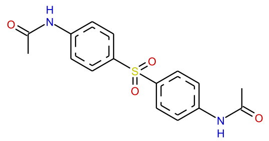 | 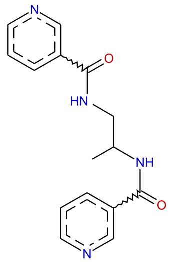 | 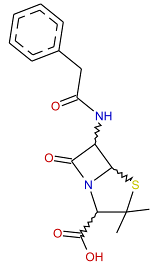 |
| Actual Endpoint | Non-Mutagen | Non-Mutagen | Non-Mutagen |
| Predicted Endpoint | Non-Mutagen | Non-Mutagen | Non-Mutagen |
| Distance | 0.549 | 0.570 | 0.581 |
| Reference | Kazius et. al., J. Med. Chem. (2005) 48, 312-320 | Kazius et. al., J. Med. Chem. (2005) 48, 312-320 | EMIC |

## Model Prediction

Prediction: Non-Mutagen

Probability: 0.716

Enrichment: 1.28

Bayesian Score: -1.11

Mahalanobis Distance: 7.98

Mahalanobis Distance p-value: 0.993

Prediction: Positive if the Bayesian score is above the estimated best cutoff value from minimizing the false positive and false negative rate.
Probability: The esimated probability that the sample is in the positive category. This assumes that the Bayesian score follows a normal distribution and is different from the prediction using a cutoff.
Enrichment: An estimate of enrichment, that is, the increased likelihood (versus random) of this sample being in the category.
Bayesian Score: The standard Laplacian-modified Bayesian score.
Mahalanobis Distance: The Mahalanobis distance (MD) is the distance to the center of the training data. The larger the MD, the less trustworthy the prediction.
Mahalanobis Distance p-value: The p-value gives the fraction of training data with an MD greater than or equal to the one for the given sample, assuming normally distributed data. The smaller the p-value, the less trustworthy the prediciton. For highly non-normal X properties (e.g., fingerprints), the MD p-value is wildly inaccurate.

## Model Applicability

Unknown features are fingerprint features in the query molecule, but not found or appearing too infrequently in the training set.

1. All properties and OPS components are within expected ranges.

## Feature Contribution

| Top features for positive contribution | | | | |
|---|---|---|---|---|
| **Fingerprint** | **Bit/Smiles** | **Feature Structure** | **Score** | **Mutagen in training set** |
| SCFP_12 | 555539852 | 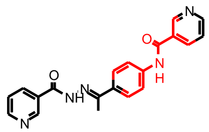 [*]:[cH]:[c](:[cH]:[*])C(=O)N[c]1:[cH]:[cH]:[*]:[cH]:[cH]:1 | 0.447 | 22 out of 24 |

| | | | | |
|---|---|---|---|---|
| SCFP_12 | 818445224 | 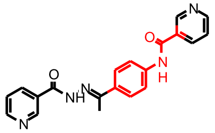 [*][c]1:[cH]:[cH]:[c](NC(=O)[c](:[*]):[*]):[cH]:[cH]:1 | 0.434 | 12 out of 13 |
| SCFP_12 | 2096901122 | 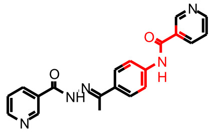 [*]:[cH]:[c](NC(=O)[c](:[*]):[*]):[cH]:[*] | 0.429 | 33 out of 37 |
| **Top Features for negative contribution** | | | | |
| **Fingerprint** | **Bit/Smiles** | **Feature Structure** | **Score** | **Mutagen in training set** |
| SCFP_12 | 1165971455 | 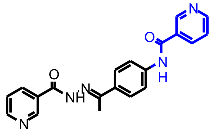 [*]NC(=O)[c]1:[cH]:[cH]:[cH]:n:[cH]:1 | -0.762 | 0 out of 2 |
| SCFP_12 | 903335088 | 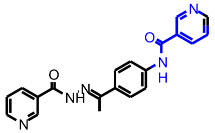 [*]NC(=O)[c]1:[cH]:[cH]:[*]:n:[cH]:1 | -0.762 | 0 out of 2 |
| SCFP_12 | -331724199 | 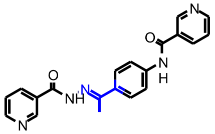 [*]\N=C(/C)\[c](:[*]):[*] | -0.762 | 0 out of 2 |

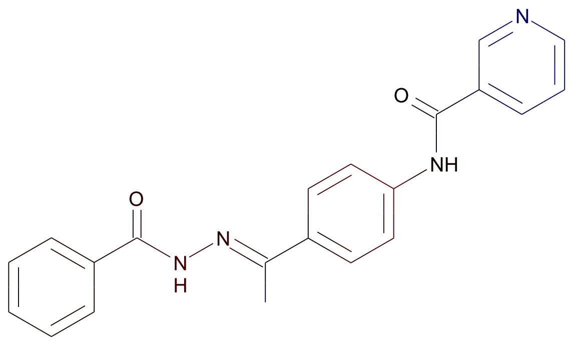

$C_{21}H_{18}N_4O_2$
Molecular Weight: 358.39322
ALogP: 2.297
Rotatable Bonds: 5
Acceptors: 4
Donors: 2

## Model Prediction

Prediction: Non-Mutagen
Probability: 0.724
Enrichment: 1.3
Bayesian Score: -0.747
Mahalanobis Distance: 7.86
Mahalanobis Distance p-value: 0.996

Prediction: Positive if the Bayesian score is above the estimated best cutoff value from minimizing the false positive and false negative rate.
Probability: The esimated probability that the sample is in the positive category. This assumes that the Bayesian score follows a normal distribution and is different from the prediction using a cutoff.
Enrichment: An estimate of enrichment, that is, the increased likelihood (versus random) of this sample being in the category.
Bayesian Score: The standard Laplacian-modified Bayesian score.
Mahalanobis Distance: The Mahalanobis distance (MD) is the distance to the center of the training data. The larger the MD, the less trustworthy the prediction.
Mahalanobis Distance p-value: The p-value gives the fraction of training data with an MD greater than or equal to the one for the given sample, assuming normally distributed data. The smaller the p-value, the less trustworthy the prediciton. For highly non-normal X properties (e.g., fingerprints), the MD p-value is wildly inaccurate.

## Structural Similar Compounds

| Name | 34433-31-3 | 89459-43-8 | 89784-39-4 |
|---|---|---|---|
| Structure | 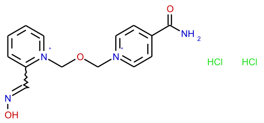 | 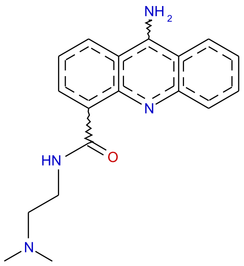 | 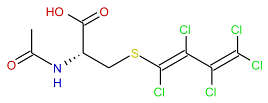 |
| Actual Endpoint | Non-Mutagen | Mutagen | Mutagen |
| Predicted Endpoint | Non-Mutagen | Mutagen | Mutagen |
| Distance | 0.499 | 0.548 | 0.562 |
| Reference | Kazius et. al., J. Med. Chem. (2005) 48, 312-320 | Kazius et. al., J. Med. Chem. (2005) 48, 312-320 | Kazius et. al., J. Med. Chem. (2005) 48, 312-320 |

## Model Applicability

Unknown features are fingerprint features in the query molecule, but not found or appearing too infrequently in the training set.

1. All properties and OPS components are within expected ranges.

## Feature Contribution

| Top features for positive contribution | | | | |
|---|---|---|---|---|
| **Fingerprint** | **Bit/Smiles** | **Feature Structure** | **Score** | **Mutagen in training set** |
| SCFP_12 | 555539852 | 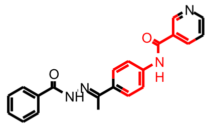 [*]:[cH]:[c](:[cH]:[*])C(=O)N[c]1:[cH]:[cH]:[*]:[cH]:[cH]:1 | 0.447 | 22 out of 24 |

| | | | | |
|---|---|---|---|---|
| SCFP_12 | 818445224 | 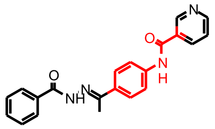  [*][c]1:[cH]:[cH]:[c] (NC(=O)[c](:[*]):[*] ):[cH]:[cH]:1 | 0.434 | 12 out of 13 |
| SCFP_12 | 2096901122 | 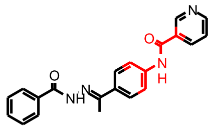  [*]:[cH]:[c](NC(=O)[c ](:[*]):[*]):[cH]:[* ] | 0.429 | 33 out of 37 |

## Top Features for negative contribution

| Fingerprint | Bit/Smiles | Feature Structure | Score | Mutagen in training set |
|---|---|---|---|---|
| SCFP_12 | -331724199 | 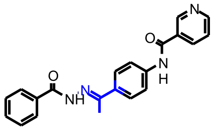  [*]\N=C(/C)\[c](:[*]) :[*] | -0.762 | 0 out of 2 |
| SCFP_12 | 1165971455 | 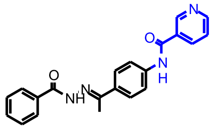  [*]NC(=O)[c]1:[cH]:[c H]:[cH]:n:[cH]:1 | -0.762 | 0 out of 2 |
| SCFP_12 | 903335088 | 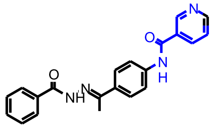  [*]NC(=O)[c]1:[cH]:[c H]:[*]:n:[cH]:1 | -0.762 | 0 out of 2 |

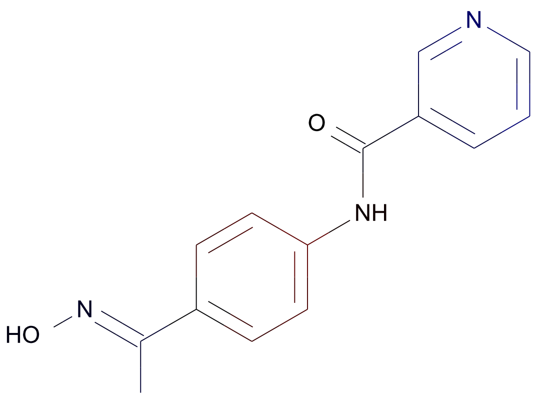


$C_{14}H_{13}N_3O_2$

Molecular Weight: 255.27191

ALogP: 1.271

Rotatable Bonds: 3

Acceptors: 4

Donors: 2

## Model Prediction

Prediction: Non-Mutagen

Probability: 0.718

Enrichment: 1.29

Bayesian Score: -1.02

Mahalanobis Distance: 7.73

Mahalanobis Distance p-value: 0.998

Prediction: Positive if the Bayesian score is above the estimated best cutoff value from minimizing the false positive and false negative rate.
Probability: The esimated probability that the sample is in the positive category. This assumes that the Bayesian score follows a normal distribution and is different from the prediction using a cutoff.
Enrichment: An estimate of enrichment, that is, the increased likelihood (versus random) of this sample being in the category.
Bayesian Score: The standard Laplacian-modified Bayesian score.
Mahalanobis Distance: The Mahalanobis distance (MD) is the distance to the center of the training data. The larger the MD, the less trustworthy the prediction.
Mahalanobis Distance p-value: The p-value gives the fraction of training data with an MD greater than or equal to the one for the given sample, assuming normally distributed data. The smaller the p-value, the less trustworthy the prediciton. For highly non-normal X properties (e.g., fingerprints), the MD p-value is wildly inaccurate.

## Structural Similar Compounds

| Name | Cycloheximide | 2687-41-4 | 19077-98-6 |
|---|---|---|---|
| Structure | 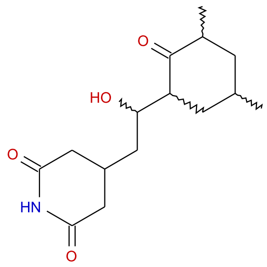 | 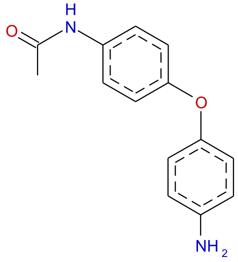 | 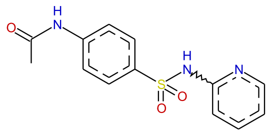 |
| Actual Endpoint | Non-Mutagen | Mutagen | Non-Mutagen |
| Predicted Endpoint | Non-Mutagen | Mutagen | Non-Mutagen |
| Distance | 0.518 | 0.518 | 0.526 |
| Reference | Environ. Mol. Mut. 19(21):1992 | Kazius et. al., J. Med. Chem. (2005) 48, 312-320 | Kazius et. al., J. Med. Chem. (2005) 48, 312-320 |

## Model Applicability

Unknown features are fingerprint features in the query molecule, but not found or appearing too infrequently in the training set.

1. All properties and OPS components are within expected ranges.

## Feature Contribution

### Top features for positive contribution

| Fingerprint | Bit/Smiles | Feature Structure | Score | Mutagen in training set |
|---|---|---|---|---|
| SCFP_12 | 555539852 | 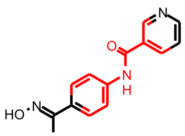 [*]:[cH]:[c](:[cH]:[*])C(=O)N[c]1:[cH]:[cH]:[*]:[cH]:[cH]:1 | 0.447 | 22 out of 24 |

| | | | | |
|---|---|---|---|---|
| SCFP_12 | 818445224 | 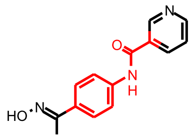 [*][c]1:[cH]:[cH]:[c](NC(=O)[c](:[*]):[*]):[cH]:[cH]:1 | 0.434 | 12 out of 13 |
| SCFP_12 | 2096901122 | 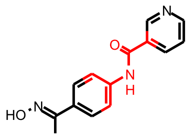 [*]:[cH]:[c](NC(=O)[c](:[*]):[*]):[cH]:[*] | 0.429 | 33 out of 37 |
| **Top Features for negative contribution** | | | | |
| **Fingerprint** | **Bit/Smiles** | **Feature Structure** | **Score** | **Mutagen in training set** |
| SCFP_12 | 1165971455 | 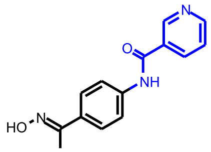 [*]NC(=O)[c]1:[cH]:[cH]:[cH]:n:[cH]:1 | -0.762 | 0 out of 2 |
| SCFP_12 | -331724199 | 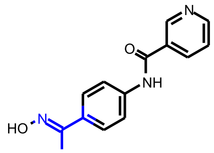 [*]\N=C(/C)\[c](:[*]):[*] | -0.762 | 0 out of 2 |
| SCFP_12 | 903335088 | 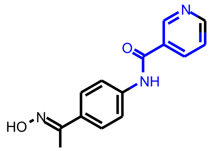 [*]NC(=O)[c]1:[cH]:[cH]:[*]:n:[cH]:1 | -0.762 | 0 out of 2 |

# D-1 TOPKAT_Ames_Mutagenicity

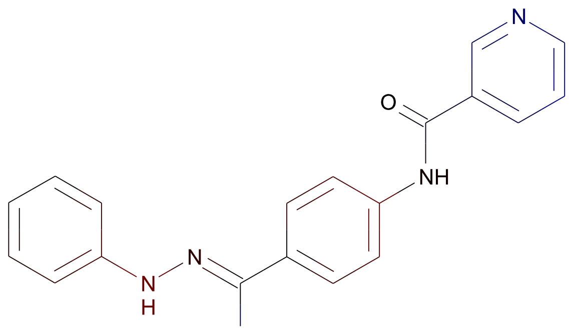

$C_{20}H_{18}N_4O$
Molecular Weight: 330.38312
ALogP: 3.063
Rotatable Bonds: 5
Acceptors: 4
Donors: 2

## Model Prediction

Prediction: Mutagen
Probability: 0.75
Enrichment: 1.34
Bayesian Score: 0.586
Mahalanobis Distance: 8.09
Mahalanobis Distance p-value: 0.989

Prediction: Positive if the Bayesian score is above the estimated best cutoff value from minimizing the false positive and false negative rate.
Probability: The esimated probability that the sample is in the positive category.  This assumes that the Bayesian score follows a normal distribution and is different from the prediction using a cutoff.
Enrichment: An estimate of enrichment, that is, the increased likelihood (versus random) of this sample being in the category.
Bayesian Score: The standard Laplacian-modified Bayesian score.
Mahalanobis Distance: The Mahalanobis distance (MD) is the distance to the center of the training data. The larger the MD, the less trustworthy the prediction.
Mahalanobis Distance p-value:  The p-value gives the fraction of training data with an MD greater than or equal to the one for the given sample, assuming normally distributed data. The smaller the p-value, the less trustworthy the prediciton. For highly non-normal X properties (e.g., fingerprints), the MD p-value is wildly inaccurate.

## Structural Similar Compounds

| Name | 4394-00-7 | Phenmedipham | 112022-13-6 |
|---|---|---|---|
| Structure | 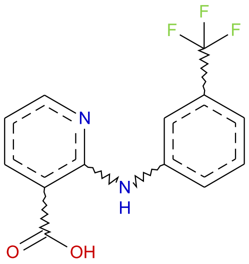 | 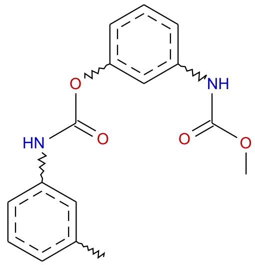 | 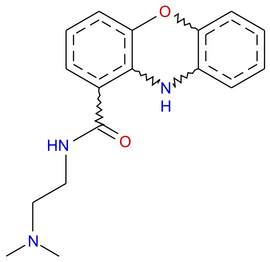 |
| Actual Endpoint | Non-Mutagen | Mutagen | Mutagen |
| Predicted Endpoint | Non-Mutagen | Mutagen | Mutagen |
| Distance | 0.528 | 0.535 | 0.538 |
| Reference | Kazius et. al., J. Med. Chem. (2005) 48, 312-320 | Environ. Mol. Mut. 19(21):1992 | Kazius et. al., J. Med. Chem. (2005) 48, 312-320 |

## Model Applicability

Unknown features are fingerprint features in the query molecule, but not found or appearing too infrequently in the training set.

1. All properties and OPS components are within expected ranges.

## Feature Contribution

**Top features for positive contribution**

| Fingerprint | Bit/Smiles | Feature Structure | Score | Mutagen in training set |
|---|---|---|---|---|
| SCFP_12 | 555539852 | 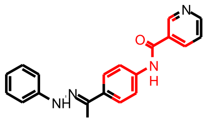 [*]:[cH]:[c](:[cH]:[* ])C(=O)N[c]1:[cH]:[c H]:[*]:[cH]:[cH]:1 | 0.447 | 22 out of 24 |

| | | | | |
|---|---|---|---|---|
| SCFP_12 | 818445224 | 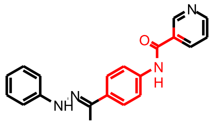 [*][c]1:[cH]:[cH]:[c](NC(=O)[c](:[*]):[*]):[cH]:[cH]:1 | 0.434 | 12 out of 13 |
| SCFP_12 | 124026986 | 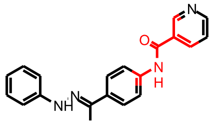 [*]:[cH]:[c](:[cH]:[*])C(=O)N[c](:[*]):[*] | 0.429 | 33 out of 37 |
| **Top Features for negative contribution** | | | | |
| **Fingerprint** | **Bit/Smiles** | **Feature Structure** | **Score** | **Mutagen in training set** |
| SCFP_12 | 1165971455 | 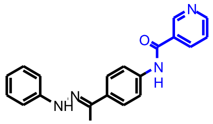 [*]NC(=O)[c]1:[cH]:[cH]:[cH]:n:[cH]:1 | -0.762 | 0 out of 2 |
| SCFP_12 | -331724199 | 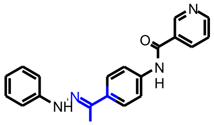 [*]\N=C(/C)\[c](:[*]):[*] | -0.762 | 0 out of 2 |
| SCFP_12 | 903335088 | 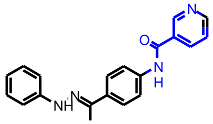 [*]NC(=O)[c]1:[cH]:[cH]:[*]:n:[cH]:1 | -0.762 | 0 out of 2 |

# Sorafenib

# TOPKAT_Ames_Mutagenicity

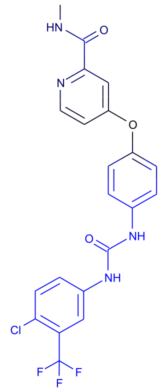

$C_{21}H_{16}ClF_3N_4O_3$

Molecular Weight: 464.82494

ALogP: 4.175

Rotatable Bonds: 6

Acceptors: 4

Donors: 3

## Structural Similar Compounds

| Name | GLYBURIDE | 38914-96-4 | 93957-54-1 |
|---|---|---|---|
| Structure | 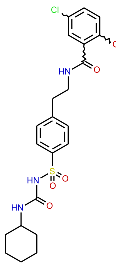 | 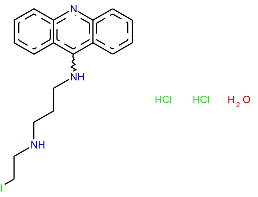 | 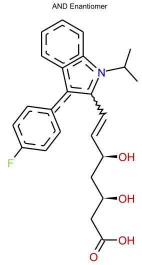 |
| Actual Endpoint | Non-Mutagen | Mutagen | Non-Mutagen |
| Predicted Endpoint | Non-Mutagen | Mutagen | Non-Mutagen |
| Distance | 0.590 | 0.592 | 0.600 |
| Reference | PDR 1994 | Kazius et. al., J. Med. Chem. (2005) 48, 312-320 | US Environmental Protection Agency at http://www.epa.gov/NCCT/dsstox/sdf_isscan_external.html |

## Model Prediction

Prediction: Non-Mutagen

Probability: 0.0531

Enrichment: 0.0951

Bayesian Score: -19.7

Mahalanobis Distance: 13.1

Mahalanobis Distance p-value: 2.73e-006

Prediction: Positive if the Bayesian score is above the estimated best cutoff value from minimizing the false positive and false negative rate.
Probability: The esimated probability that the sample is in the positive category. This assumes that the Bayesian score follows a normal distribution and is different from the prediction using a cutoff.
Enrichment: An estimate of enrichment, that is, the increased likelihood (versus random) of this sample being in the category.
Bayesian Score: The standard Laplacian-modified Bayesian score.
Mahalanobis Distance: The Mahalanobis distance (MD) is the distance to the center of the training data. The larger the MD, the less trustworthy the prediction.
Mahalanobis Distance p-value: The p-value gives the fraction of training data with an MD greater than or equal to the one for the given sample, assuming normally distributed data. The smaller the p-value, the less trustworthy the prediciton. For highly non-normal X properties (e.g., fingerprints), the MD p-value is wildly inaccurate.

## Model Applicability

Unknown features are fingerprint features in the query molecule, but not found or appearing too infrequently in the training set.

1. All properties and OPS components are within expected ranges.

## Feature Contribution

| Top features for positive contribution | | | | |
|---|---|---|---|---|
| **Fingerprint** | **Bit/Smiles** | **Feature Structure** | **Score** | **Mutagen in training set** |
| SCFP_12 | -347281112 | 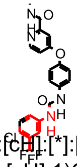 [*]N[c]1:[cH]:[*]:[c]([*]):[c](:[cH]:1)C([*])([*])[*] | 0.337 | 18 out of 22 |

| | | | | |
|---|---|---|---|---|
| SCFP_12 | 1208843554 | 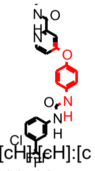 [*]N[c]1:[cH]:[cH]:[c](O[c](:[*]):[*]):[cH]:[cH]:1 | 0.337 | 6 out of 7 |
| SCFP_12 | -1943080297 | 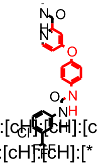 [*]N[c]1:[cH]:[cH]:[c](O[c]2:[cH]:[cH]:[*]:[c]([*]):[cH]:2):[cH]:[cH]:1 | 0.304 | 5 out of 6 |
| **Top Features for negative contribution** | | | | |
| **Fingerprint** | **Bit/Smiles** | **Feature Structure** | **Score** | **Mutagen in training set** |
| SCFP_12 | 816802409 | 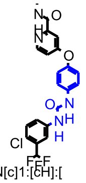 [*]NC(=O)N[c]1:[cH]:[cH]:[c]([*]):[cH]:[cH]:1 | -1.82 | 0 out of 9 |
| SCFP_12 | -1903175541 | 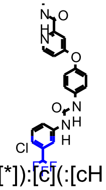 [*][c](:[*]):[c](:[cH]:[*])C(F)(F)F | -1.51 | 3 out of 30 |
| SCFP_12 | -300280774 | 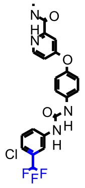 [*]:[c](:[*])C(F)(F)F | -1.51 | 3 out of 30 |

# TOPKAT_Mouse_Female_FDA_None_vs_Carcinogen

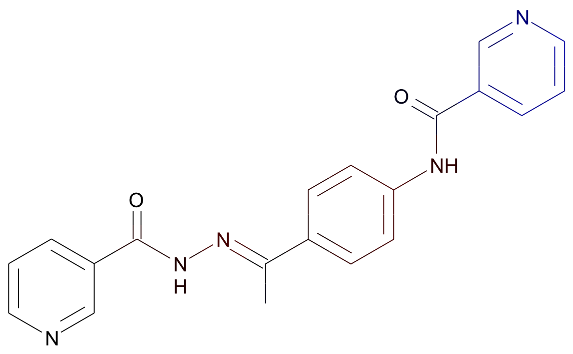

$C_{20}H_{17}N_5O_2$

Molecular Weight: 359.38128

ALogP: 1.147

Rotatable Bonds: 5

Acceptors: 5

Donors: 2

## Structural Similar Compounds

| Name | Acetohexamide | Alizapride | Penicillin |
|---|---|---|---|
| Structure | 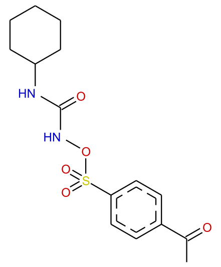 | 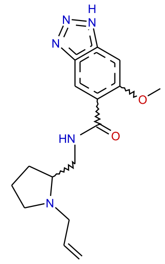 | 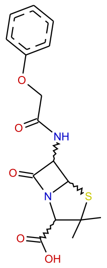 |
| Actual Endpoint | Non-Carcinogen | Carcinogen | Non-Carcinogen |
| Predicted Endpoint | Non-Carcinogen | Carcinogen | Non-Carcinogen |
| Distance | 0.578 | 0.581 | 0.603 |
| Reference | US FDA (Centre for Drug Eval.& Res./Off. Testing & Res.) Sept. 1997 | US FDA (Centre for Drug Eval.& Res./Off. Testing & Res.) Sept. 1997 | US FDA (Centre for Drug Eval.& Res./Off. Testing & Res.) Sept. 1997 |

## Model Prediction

Prediction: Non-Carcinogen

Probability: 0.242

Enrichment: 0.755

Bayesian Score: -1.2

Mahalanobis Distance: 11

Mahalanobis Distance p-value: 0.0949

Prediction: Positive if the Bayesian score is above the estimated best cutoff value from minimizing the false positive and false negative rate.
Probability: The esimated probability that the sample is in the positive category. This assumes that the Bayesian score follows a normal distribution and is different from the prediction using a cutoff.
Enrichment: An estimate of enrichment, that is, the increased likelihood (versus random) of this sample being in the category.
Bayesian Score: The standard Laplacian-modified Bayesian score.
Mahalanobis Distance: The Mahalanobis distance (MD) is the distance to the center of the training data. The larger the MD, the less trustworthy the prediction.
Mahalanobis Distance p-value: The p-value gives the fraction of training data with an MD greater than or equal to the one for the given sample, assuming normally distributed data. The smaller the p-value, the less trustworthy the prediciton. For highly non-normal X properties (e.g., fingerprints), the MD p-value is wildly inaccurate.

## Model Applicability

Unknown features are fingerprint features in the query molecule, but not found or appearing too infrequently in the training set.

1. All properties and OPS components are within expected ranges.
2. Unknown ECFP_2 feature: 128986386: [*]\N=C(/C)\[c](:[*]):[*]
3. Unknown ECFP_2 feature: 560380707: [*]NN=C([*])[*]

## Feature Contribution

**Top features for positive contribution**

| Fingerprint | Bit/Smiles | Feature Structure | Score | Carcinogen in training set |
|---|---|---|---|---|
| ECFP_6 | -1087070950 | 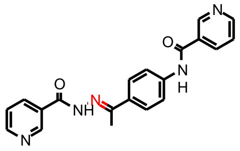 [*]N=[*] | 0.724 | 10 out of 14 |

| | | | | |
|---|---|---|---|---|
| ECFP_6 | 544048674 | 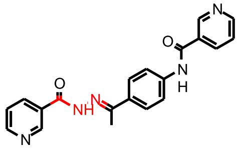 [*]C(=[*])NN=[*] | 0.617 | 2 out of 2 |
| ECFP_6 | 738938915 | 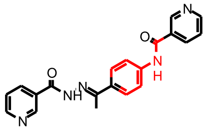 [*]C(=[*])N[c]1:[cH]:[cH]:[*]:[cH]:[cH]:1 | 0.617 | 2 out of 2 |
| **Top Features for negative contribution** | | | | |
| **Fingerprint** | **Bit/Smiles** | **Feature Structure** | **Score** | **Carcinogen in training set** |
| ECFP_6 | 2013347047 | 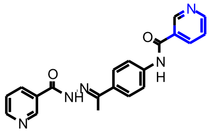 [*][c]1:[*]:n:[cH]:[cH]:[cH]:1 | -0.805 | 0 out of 4 |
| ECFP_6 | 764951226 | 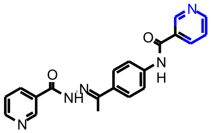 [*]1:[cH]:[cH]:[cH]:n:[cH]:1 | -0.482 | 0 out of 2 |
| ECFP_6 | -1818873508 | 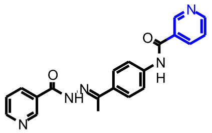 [*][c]1:[cH]:[cH]:[cH]:n:[cH]:1 | -0.482 | 0 out of 2 |

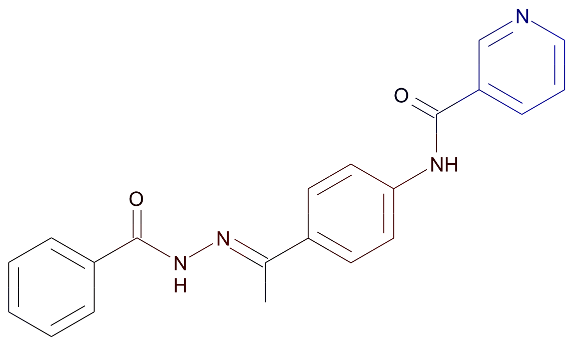

$C_{21}H_{18}N_4O_2$
Molecular Weight: 358.39322
ALogP: 2.297
Rotatable Bonds: 5
Acceptors: 4
Donors: 2

## Model Prediction

Prediction: Non-Carcinogen
Probability: 0.231
Enrichment: 0.721
Bayesian Score: -1.96
Mahalanobis Distance: 11.7
Mahalanobis Distance p-value: 0.0236

Prediction: Positive if the Bayesian score is above the estimated best cutoff value from minimizing the false positive and false negative rate.
Probability: The esimated probability that the sample is in the positive category. This assumes that the Bayesian score follows a normal distribution and is different from the prediction using a cutoff.
Enrichment: An estimate of enrichment, that is, the increased likelihood (versus random) of this sample being in the category.
Bayesian Score: The standard Laplacian-modified Bayesian score.
Mahalanobis Distance: The Mahalanobis distance (MD) is the distance to the center of the training data. The larger the MD, the less trustworthy the prediction.
Mahalanobis Distance p-value: The p-value gives the fraction of training data with an MD greater than or equal to the one for the given sample, assuming normally distributed data. The smaller the p-value, the less trustworthy the prediciton. For highly non-normal X properties (e.g., fingerprints), the MD p-value is wildly inaccurate.

## Structural Similar Compounds

| Name | Mebendazole | Alizapride | Indapamide |
|---|---|---|---|
| Structure | 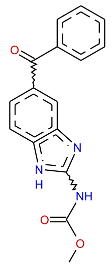 | 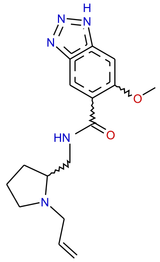 | 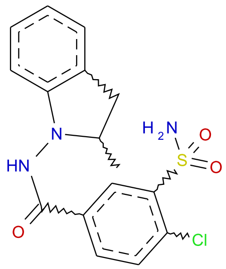 |
| Actual Endpoint | Non-Carcinogen | Carcinogen | Non-Carcinogen |
| Predicted Endpoint | Non-Carcinogen | Carcinogen | Non-Carcinogen |
| Distance | 0.570 | 0.601 | 0.601 |
| Reference | US FDA (Centre for Drug Eval.& Res./Off. Testing & Res.) Sept. 1997 | US FDA (Centre for Drug Eval.& Res./Off. Testing & Res.) Sept. 1997 | US FDA (Centre for Drug Eval.& Res./Off. Testing & Res.) Sept. 1997 |

## Model Applicability

Unknown features are fingerprint features in the query molecule, but not found or appearing too infrequently in the training set.

1. All properties and OPS components are within expected ranges.
2. Unknown ECFP_2 feature: 128986386: [*]\N=C(/C)\[c](:[*]):[*]
3. Unknown ECFP_2 feature: 560380707: [*]NN=C([*])[*]

## Feature Contribution

**Top features for positive contribution**

| Fingerprint | Bit/Smiles | Feature Structure | Score | Carcinogen in training set |
|---|---|---|---|---|
| ECFP_6 | -1087070950 | 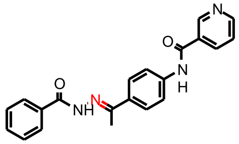 [*]N=[*] | 0.724 | 10 out of 14 |

| | | | | |
|---|---|---|---|---|
| ECFP_6 | 544048674 | 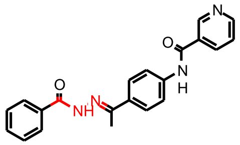 [*]C(=[*])NN=[*] | 0.617 | 2 out of 2 |
| ECFP_6 | 738938915 | 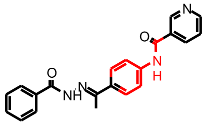 [*]C(=[*])N[c]1:[cH]:[cH]:[*]:[cH]:[cH]:1 | 0.617 | 2 out of 2 |

**Top Features for negative contribution**

| Fingerprint | Bit/Smiles | Feature Structure | Score | Carcinogen in training set |
|---|---|---|---|---|
| ECFP_6 | 2013347047 | 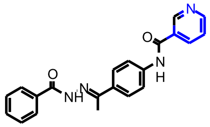 [*][c]1:[*]:n:[cH]:[cH]:[cH]:1 | -0.805 | 0 out of 4 |
| ECFP_6 | 764951226 | 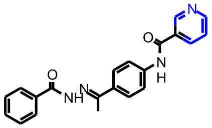 [*]1:[cH]:[cH]:[cH]:n:[cH]:1 | -0.482 | 0 out of 2 |
| ECFP_6 | -1818873508 | 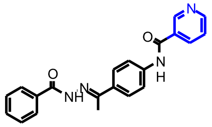 [*][c]1:[cH]:[cH]:[cH]:n:[cH]:1 | -0.482 | 0 out of 2 |

# TOPKAT_Mouse_Female_FDA_None_vs_Carcinogen

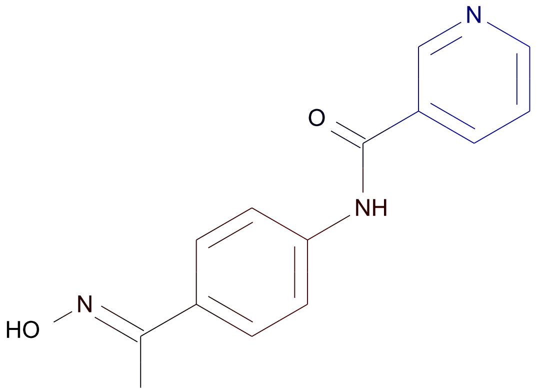


$C_{14}H_{13}N_3O_2$

Molecular Weight: 255.27191

ALogP: 1.271

Rotatable Bonds: 3

Acceptors: 4

Donors: 2

## Model Prediction

Prediction: Non-Carcinogen

Probability: 0.233

Enrichment: 0.726

Bayesian Score: -1.83

Mahalanobis Distance: 10.9

Mahalanobis Distance p-value: 0.121

Prediction: Positive if the Bayesian score is above the estimated best cutoff value from minimizing the false positive and false negative rate.
Probability: The esimated probability that the sample is in the positive category. This assumes that the Bayesian score follows a normal distribution and is different from the prediction using a cutoff.
Enrichment: An estimate of enrichment, that is, the increased likelihood (versus random) of this sample being in the category.
Bayesian Score: The standard Laplacian-modified Bayesian score.
Mahalanobis Distance: The Mahalanobis distance (MD) is the distance to the center of the training data. The larger the MD, the less trustworthy the prediction.
Mahalanobis Distance p-value: The p-value gives the fraction of training data with an MD greater than or equal to the one for the given sample, assuming normally distributed data. The smaller the p-value, the less trustworthy the prediciton. For highly non-normal X properties (e.g., fingerprints), the MD p-value is wildly inaccurate.

## Structural Similar Compounds

| Name | Tolazamide | Phenobarbital | Chlorpropamide |
|---|---|---|---|
| Structure | 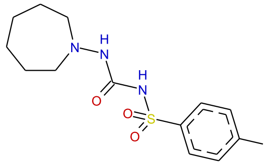 | 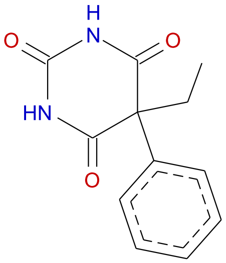 | 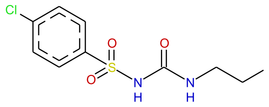 |
| Actual Endpoint | Non-Carcinogen | Carcinogen | Non-Carcinogen |
| Predicted Endpoint | Non-Carcinogen | Carcinogen | Non-Carcinogen |
| Distance | 0.576 | 0.586 | 0.591 |
| Reference | US FDA (Centre for Drug Eval.& Res./Off. Testing & Res.) Sept. 1997 | US FDA (Centre for Drug Eval.& Res./Off. Testing & Res.) Sept. 1997 | US FDA (Centre for Drug Eval.& Res./Off. Testing & Res.) Sept. 1997 |

## Model Applicability

Unknown features are fingerprint features in the query molecule, but not found or appearing too infrequently in the training set.

1. All properties and OPS components are within expected ranges.
2. Unknown ECFP_2 feature: 128986386: [*]\N=C(/C)\[c](:[*]):[*]
3. Unknown ECFP_2 feature: 559672449: [*]C(=NO)[*]
4. Unknown ECFP_2 feature: 2025254696: [*]=NO

## Feature Contribution

| Top features for positive contribution | | | | |
|---|---|---|---|---|
| **Fingerprint** | **Bit/Smiles** | **Feature Structure** | **Score** | **Carcinogen in training set** |
| ECFP_6 | -1087070950 | 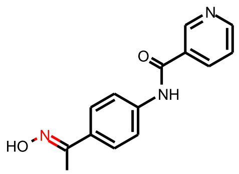 [*]N=[*] | 0.724 | 10 out of 14 |

| | | | | |
|---|---|---|---|---|
| ECFP_6 | 738938915 | 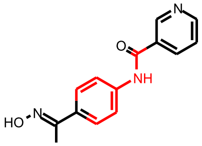 [*]C(=[*])N[c]1:[cH]:[cH]:[*]:[cH]:[cH]:1 | 0.617 | 2 out of 2 |
| ECFP_6 | -177077903 | 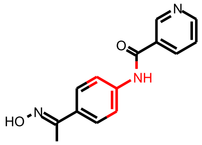 [*]N[c](:[cH]:[*]):[cH]:[*] | 0.279 | 4 out of 9 |
| **Top Features for negative contribution** | | | | |
| **Fingerprint** | **Bit/Smiles** | **Feature Structure** | **Score** | **Carcinogen in training set** |
| ECFP_6 | 2013347047 | 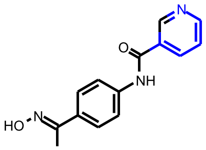 [*][c]1:[*]:n:[cH]:[cH]:[cH]:1 | -0.805 | 0 out of 4 |
| ECFP_6 | -1818873508 | 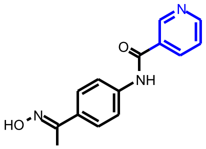 [*][c]1:[cH]:[cH]:[cH]:n:[cH]:1 | -0.482 | 0 out of 2 |
| ECFP_6 | 764951226 | 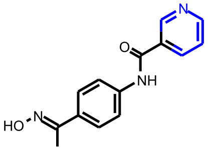 [*]1:[cH]:[cH]:[cH]:n:[cH]:1 | -0.482 | 0 out of 2 |

# TOPKAT_Mouse_Female_FDA_None_vs_Carcinogen

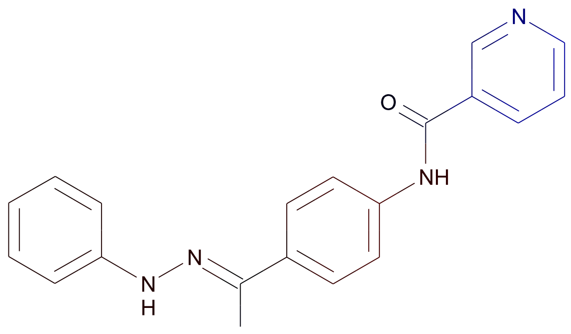

$C_{20}H_{18}N_4O$
Molecular Weight: 330.38312
ALogP: 3.063
Rotatable Bonds: 5
Acceptors: 4
Donors: 2

## Model Prediction

Prediction: Non-Carcinogen
Probability: 0.234
Enrichment: 0.729
Bayesian Score: -1.77
Mahalanobis Distance: 11.7
Mahalanobis Distance p-value: 0.0253

Prediction: Positive if the Bayesian score is above the estimated best cutoff value from minimizing the false positive and false negative rate.
Probability: The esimated probability that the sample is in the positive category. This assumes that the Bayesian score follows a normal distribution and is different from the prediction using a cutoff.
Enrichment: An estimate of enrichment, that is, the increased likelihood (versus random) of this sample being in the category.
Bayesian Score: The standard Laplacian-modified Bayesian score.
Mahalanobis Distance: The Mahalanobis distance (MD) is the distance to the center of the training data. The larger the MD, the less trustworthy the prediction.
Mahalanobis Distance p-value: The p-value gives the fraction of training data with an MD greater than or equal to the one for the given sample, assuming normally distributed data. The smaller the p-value, the less trustworthy the prediciton. For highly non-normal X properties (e.g., fingerprints), the MD p-value is wildly inaccurate.

## Structural Similar Compounds

| Name | Mebendazole | Bunolol | Etodolac |
|---|---|---|---|
| Structure | 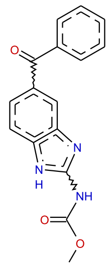 | 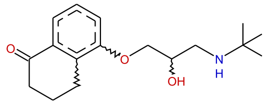 | 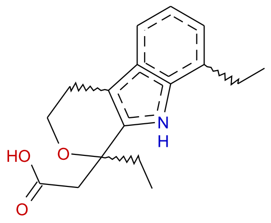 |
| Actual Endpoint | Non-Carcinogen | Carcinogen | Non-Carcinogen |
| Predicted Endpoint | Non-Carcinogen | Carcinogen | Non-Carcinogen |
| Distance | 0.559 | 0.582 | 0.606 |
| Reference | US FDA (Centre for Drug Eval.& Res./Off. Testing & Res.) Sept. 1997 | US FDA (Centre for Drug Eval.& Res./Off. Testing & Res.) Sept. 1997 | US FDA (Centre for Drug Eval.& Res./Off. Testing & Res.) Sept. 1997 |

## Model Applicability

Unknown features are fingerprint features in the query molecule, but not found or appearing too infrequently in the training set.

1. All properties and OPS components are within expected ranges.
2. Unknown ECFP_2 feature: 128986386: [*]\N=C(/C)\[c](:[*]):[*]
3. Unknown ECFP_2 feature: 560380707: [*]NN=C([*])[*]
4. Unknown ECFP_2 feature: -1236714312: [*]=NN[c](:[*]):[*]

## Feature Contribution

| Top features for positive contribution | | | | |
|---|---|---|---|---|
| **Fingerprint** | **Bit/Smiles** | **Feature Structure** | **Score** | **Carcinogen in training set** |
| ECFP_6 | -1087070950 | 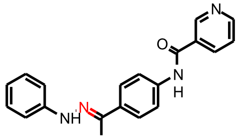 [*]N=[*] | 0.724 | 10 out of 14 |

| | | | | |
|---|---|---|---|---|
| ECFP_6 | 738938915 | 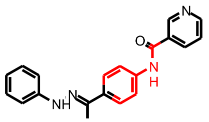 [*]C(=[*])N[c]1:[cH]:[cH]:[*]:[cH]:[cH]:1 | 0.617 | 2 out of 2 |
| ECFP_6 | 547788473 | 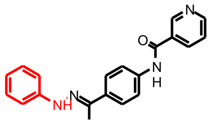 [*]N[c]1:[cH]:[cH]:[cH]:[cH]:[cH]:1 | 0.424 | 1 out of 1 |

## Top Features for negative contribution

| Fingerprint | Bit/Smiles | Feature Structure | Score | Carcinogen in training set |
|---|---|---|---|---|
| ECFP_6 | 2013347047 | 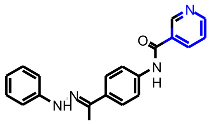 [*][c]1:[*]:n:[cH]:[cH]:[cH]:1 | -0.805 | 0 out of 4 |
| ECFP_6 | -1818873508 | 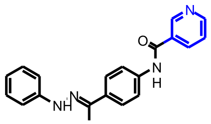 [*][c]1:[cH]:[cH]:[cH]:n:[cH]:1 | -0.482 | 0 out of 2 |
| ECFP_6 | 764951226 | 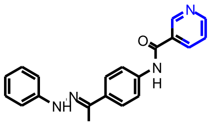 [*]1:[cH]:[cH]:[cH]:n:[cH]:1 | -0.482 | 0 out of 2 |

# Sorafenib

# TOPKAT_Mouse_Female_FDA_None_vs_Carcinogen

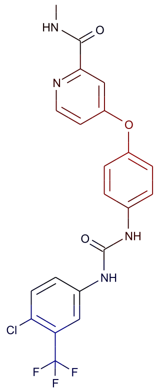


$C_{21}H_{16}ClF_3N_4O_3$
Molecular Weight: 464.82494
ALogP: 4.175
Rotatable Bonds: 6
Acceptors: 4
Donors: 3

## Model Prediction

Prediction: Carcinogen
Probability: 0.257
Enrichment: 0.801
Bayesian Score: -0.321
Mahalanobis Distance: 14.9
Mahalanobis Distance p-value: 4.21e-007

Prediction: Positive if the Bayesian score is above the estimated best cutoff value from minimizing the false positive and false negative rate.
Probability: The esimated probability that the sample is in the positive category. This assumes that the Bayesian score follows a normal distribution and is different from the prediction using a cutoff.
Enrichment: An estimate of enrichment, that is, the increased likelihood (versus random) of this sample being in the category.
Bayesian Score: The standard Laplacian-modified Bayesian score.
Mahalanobis Distance: The Mahalanobis distance (MD) is the distance to the center of the training data. The larger the MD, the less trustworthy the prediction.
Mahalanobis Distance p-value: The p-value gives the fraction of training data with an MD greater than or equal to the one for the given sample, assuming normally distributed data. The smaller the p-value, the less trustworthy the prediciton. For highly non-normal X properties (e.g., fingerprints), the MD p-value is wildly inaccurate.

## Structural Similar Compounds

| Name | Glimepride | Glyburide | Fluvastatin |
|---|---|---|---|
| Structure | 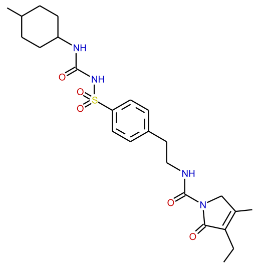 | 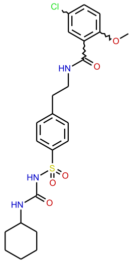 | 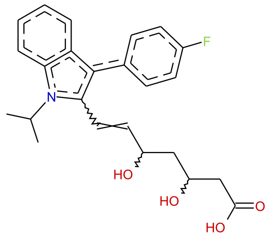 |
| Actual Endpoint | Carcinogen | Non-Carcinogen | Non-Carcinogen |
| Predicted Endpoint | Carcinogen | Non-Carcinogen | Non-Carcinogen |
| Distance | 0.605 | 0.615 | 0.625 |
| Reference | US FDA (Centre for Drug Eval.& Res./Off. Testing & Res.) Sept. 1997 | US FDA (Centre for Drug Eval.& Res./Off. Testing & Res.) Sept. 1997 | US FDA (Centre for Drug Eval.& Res./Off. Testing & Res.) Sept. 1997 |

## Model Applicability

Unknown features are fingerprint features in the query molecule, but not found or appearing too infrequently in the training set.

1. OPS PC20 out of range. Value: -3.3309. Training min, max, SD, explained variance: -3.1862, 4.4571, 1.28, 0.0167.

## Feature Contribution

**Top features for positive contribution**

| Fingerprint | Bit/Smiles | Feature Structure | Score | Carcinogen in training set |
|---|---|---|---|---|
| ECFP_6 | 738938915 | 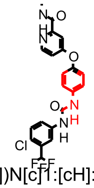 [*]C(=[*])N[c]1:[cH]:[cH]:[*]:[cH]:[cH]:1 | 0.617 | 2 out of 2 |
